# Supplementary material for: The Initial Response of a Eukaryotic Replisome to DNA Damage
Source: Mol Cell. 2018 Jun 21;70(6):1067–1080.e12. doi: 10.1016/j.molcel.2018.04.022 (PMC6024075; doi:10.1016/j.molcel.2018.04.022)
Supplement: Document S1. Figures S1–S7 and Table S1 [file mmc1.pdf]

Molecular Cell, Volume *70*

Supplemental Information

The Initial Response of a Eukaryotic Replisome to DNA Damage

Martin R.G. Taylor and Joseph T.P. Yeeles

Figure S1

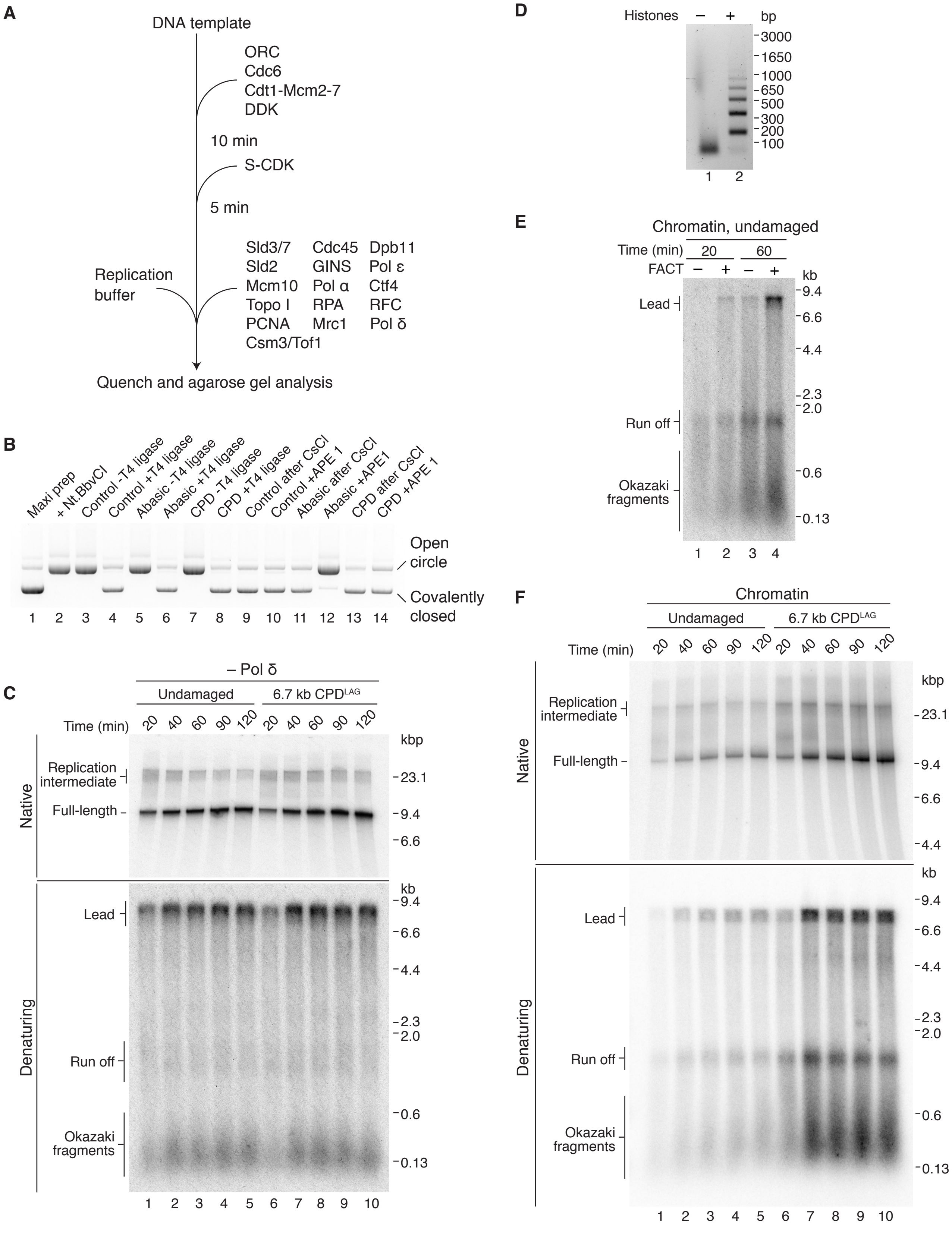
A
DNA template
ORC
Cdc6
Cdt1-Mcm2-7
DDK
10 min
S-CDK
5 min
Replication buffer
Sld3/7 Cdc45 Dpb11
Sld2 GINS Pol ε
Mcm10 Pol α Ctf4
Topo I RPA RFC
PCNA Mrc1 Pol δ
Csm3/Tof1
Quench and agarose gel analysis
B
Maxi prep
+ Nt.BbvCI
Control -T4 ligase
Control +T4 ligase
Abasic -T4 ligase
Abasic +T4 ligase
CPD -T4 ligase
CPD +T4 ligase
Control after CsCl
Control +APE 1
Abasic after CsCl
Abasic +APE1
CPD after CsCl
CPD +APE 1
Open circle
Covalently closed
1 2 3 4 5 6 7 8 9 10 11 12 13 14
C
– Pol δ
Undamaged
6.7 kb CPD^LAG
Time (min) 20 40 60 90 120 20 40 60 90 120
Native
Replication intermediate
Full-length
kbp
23.1
9.4
6.6
Denaturing
Lead
Run off
Okazaki fragments
kb
9.4
6.6
4.4
2.3
2.0
0.6
0.13
1 2 3 4 5 6 7 8 9 10
D
Histones – +
bp
3000
1650
1000
650
500
300
200
100
1 2
E
Chromatin, undamaged
Time (min) 20 60
FACT – + – +
kb
Lead
9.4
6.6
4.4
2.3
2.0
Run off
0.6
Okazaki fragments
0.13
1 2 3 4
F
Chromatin
Undamaged
6.7 kb CPD^LAG
Time (min) 20 40 60 90 120 20 40 60 90 120
Native
Replication intermediate
Full-length
kbp
23.1
9.4
6.6
4.4
Denaturing
kb
Lead
9.4
6.6
4.4
2.3
2.0
Run off
Okazaki fragments
0.6
0.13
1 2 3 4 5 6 7 8 9 10

Figure S2

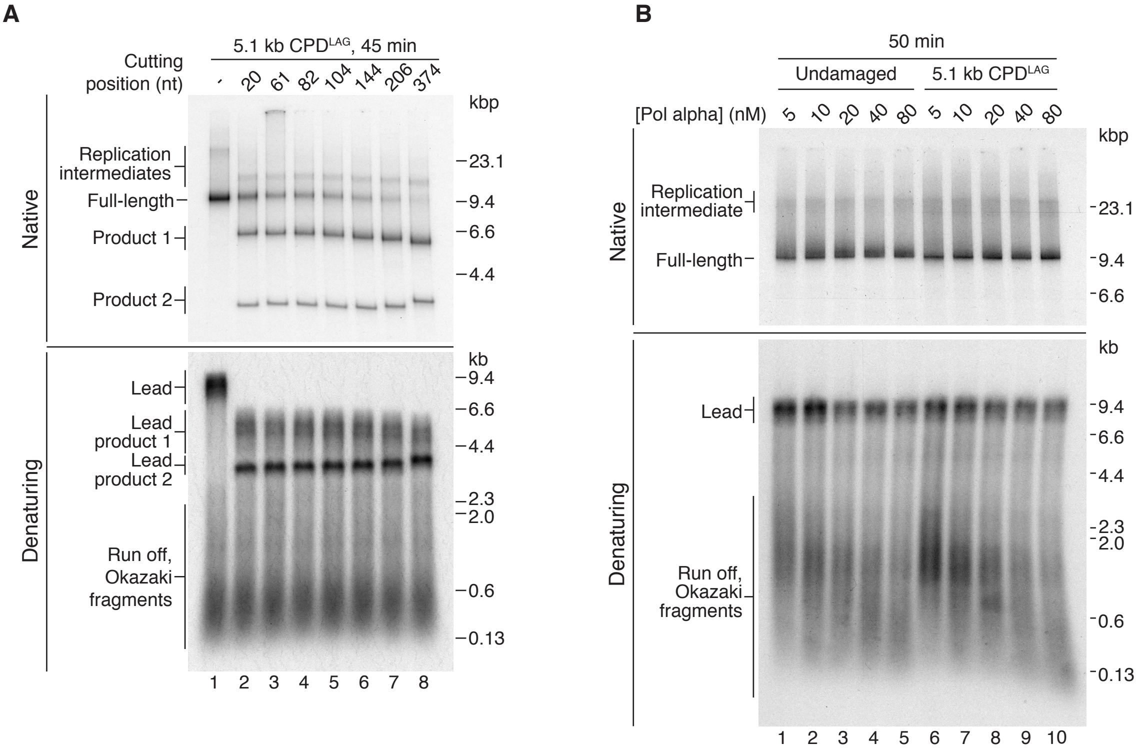
A
5.1 kb CPD^LAG, 45 min
Cutting position (nt)
-
20
61
82
104
144
206
374
kbp
Native
Replication intermediates
Full-length
Product 1
Product 2
23.1
9.4
6.6
4.4
kb
Denaturing
Lead
Lead product 1
Lead product 2
Run off, Okazaki fragments
9.4
6.6
4.4
2.3
2.0
0.6
0.13
1 2 3 4 5 6 7 8
B
50 min
Undamaged
5.1 kb CPD^LAG
[Pol alpha] (nM)
5 10 20 40 80 5 10 20 40 80
kbp
Native
Replication intermediate
Full-length
23.1
9.4
6.6
kb
Denaturing
Lead
Run off, Okazaki fragments
9.4
6.6
4.4
2.3
2.0
0.6
0.13
1 2 3 4 5 6 7 8 9 10

Figure S3

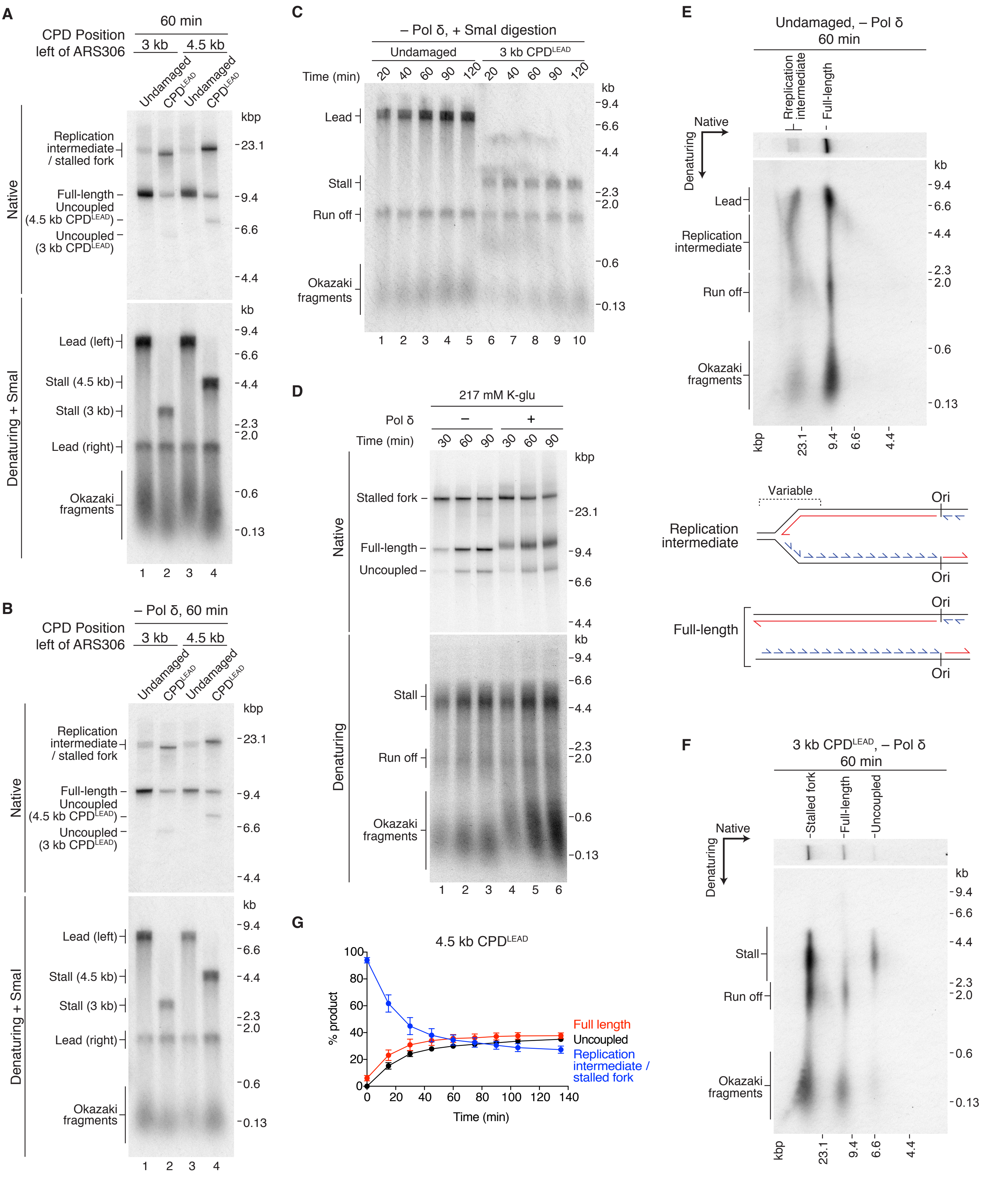
A
CPD Position left of ARS306
60 min
3 kb
4.5 kb
Undamaged
CPDLEAD
Undamaged
CPDLEAD
Native
Replication intermediate / stalled fork
Full-length
Uncoupled (4.5 kb CPDLEAD)
Uncoupled (3 kb CPDLEAD)
kbp
23.1
9.4
6.6
4.4
Denaturing + SmaI
Lead (left)
Stall (4.5 kb)
Stall (3 kb)
Lead (right)
Okazaki fragments
kb
9.4
6.6
4.4
2.3
2.0
0.6
0.13
1 2 3 4
B
CPD Position left of ARS306
– Pol δ, 60 min
3 kb
4.5 kb
C
– Pol δ, + SmaI digestion
Undamaged
3 kb CPDLEAD
Time (min)
20 40 60 90 120 20 40 60 90 120
Lead
Stall
Run off
Okazaki fragments
1 2 3 4 5 6 7 8 9 10
D
217 mM K-glu
Pol δ
–
+
Time (min)
30 60 90 30 60 90
Stalled fork
Full-length
Uncoupled
Denaturing
Stall
Run off
1 2 3 4 5 6
E
Undamaged, – Pol δ
60 min
Replication intermediate
Full-length
Native
Denaturing
Lead
Run off
kbp
Variable
Ori
Replication intermediate
Full-length
F
3 kb CPDLEAD, – Pol δ
60 min
Stalled fork
Full-length
Uncoupled
Stall
Run off
G
4.5 kb CPDLEAD
% product
100
80
60
40
20
0
Time (min)
0 20 40 60 80 100 120 140
Full length
Uncoupled
Replication intermediate / stalled fork

Figure S4

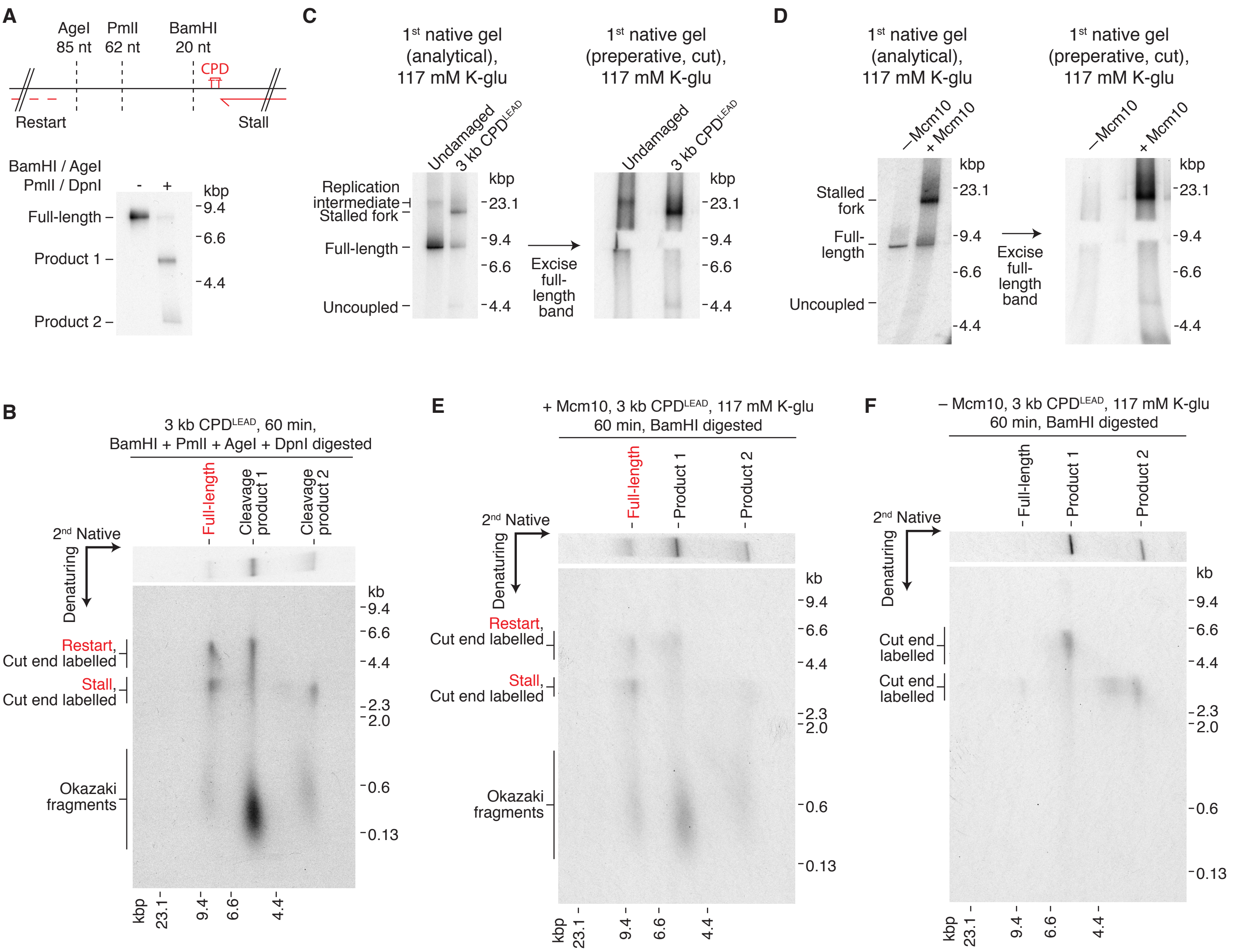

A
AgeI 85 nt
PmlI 62 nt
BamHI 20 nt
CPD
Restart
Stall
BamHI / AgeI
PmlI / DpnI
Full-length
Product 1
Product 2
kbp
9.4
6.6
4.4
C
1st native gel (analytical), 117 mM K-glu
1st native gel (preperative, cut), 117 mM K-glu
Undamaged
3 kb CPDLEAD
Replication intermediate
Stalled fork
Full-length
Uncoupled
Excise full-length band
23.1
D
–Mcm10
+ Mcm10
Stalled fork
Full-length
B
3 kb CPDLEAD, 60 min, BamHI + PmlI + AgeI + DpnI digested
Full-length
Cleavage product 1
Cleavage product 2
2nd Native
Denaturing
Restart, Cut end labelled
Stall, Cut end labelled
Okazaki fragments
kb
2.3
2.0
0.6
0.13
E
+ Mcm10, 3 kb CPDLEAD, 117 mM K-glu 60 min, BamHI digested
Product 1
Product 2
F
– Mcm10, 3 kb CPDLEAD, 117 mM K-glu 60 min, BamHI digested
Cut end labelled

Figure S5

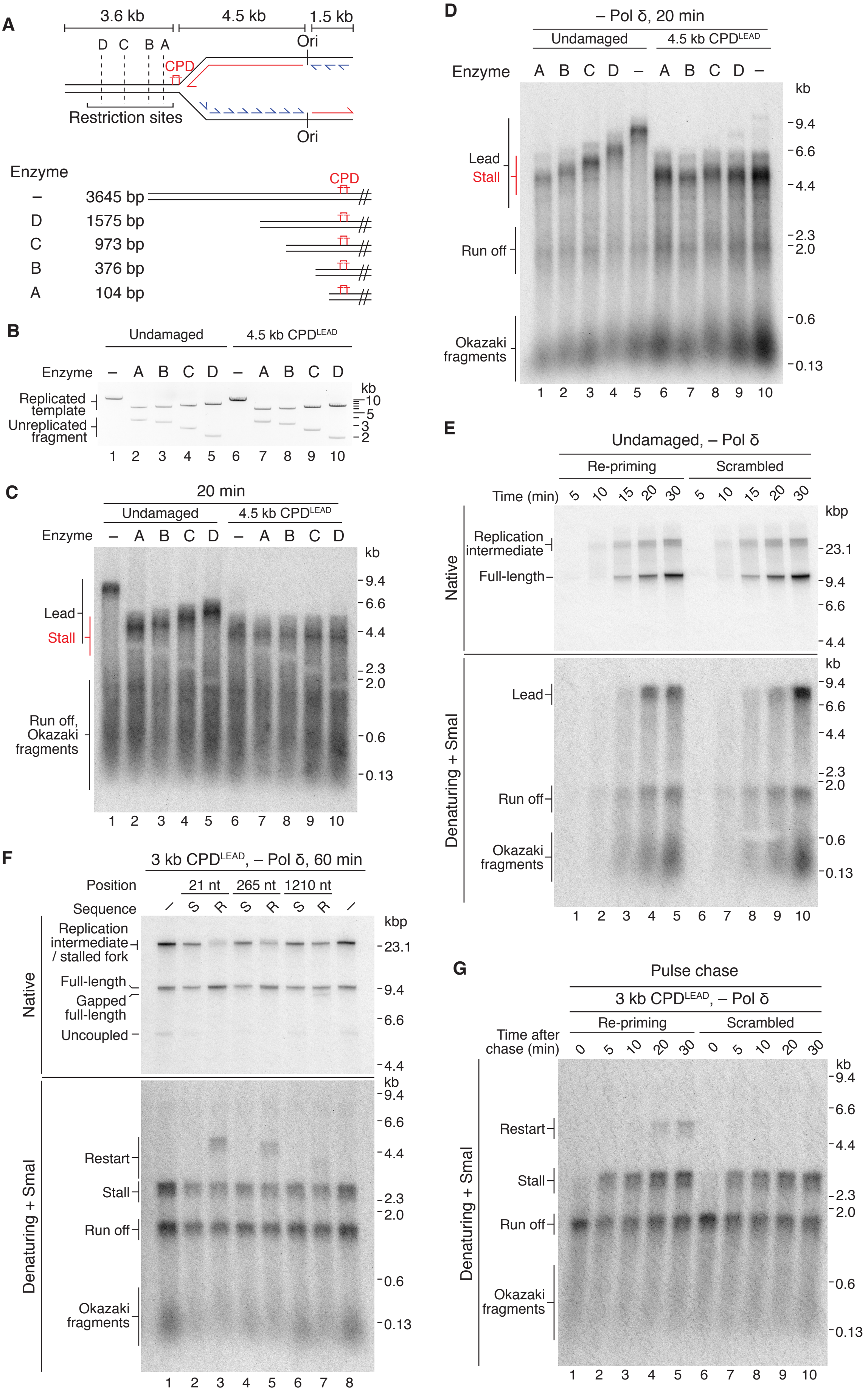
A
3.6 kb
4.5 kb
1.5 kb
D C B A
Ori
CPD
Restriction sites
Ori
Enzyme
– 3645 bp
D 1575 bp
C 973 bp
B 376 bp
A 104 bp
CPD
B
Undamaged
4.5 kb CPD LEAD
Enzyme – A B C D – A B C D
Replicated template
Unreplicated fragment
kb 10 5 3 2
1 2 3 4 5 6 7 8 9 10
C
20 min
Undamaged
4.5 kb CPD LEAD
Enzyme – A B C D – A B C D
Lead
Stall
Run off, Okazaki fragments
kb 9.4 6.6 4.4 2.3 2.0 0.6 0.13
1 2 3 4 5 6 7 8 9 10
D
– Pol δ, 20 min
Undamaged
4.5 kb CPD LEAD
Enzyme A B C D – A B C D –
Lead
Stall
Run off
Okazaki fragments
kb 9.4 6.6 4.4 2.3 2.0 0.6 0.13
1 2 3 4 5 6 7 8 9 10
E
Undamaged, – Pol δ
Re-priming
Scrambled
Time (min) 5 10 15 20 30 5 10 15 20 30
Native
Replication intermediate
Full-length
kbp 23.1 9.4 6.6 4.4
Denaturing + SmaI
Lead
Run off
Okazaki fragments
kb 9.4 6.6 4.4 2.3 2.0 0.6 0.13
1 2 3 4 5 6 7 8 9 10
F
3 kb CPD LEAD, – Pol δ, 60 min
Position 21 nt 265 nt 1210 nt
Sequence / S R S R S R /
Native
Replication intermediate / stalled fork
Full-length
Gapped full-length
Uncoupled
kbp 23.1 9.4 6.6 4.4
Denaturing + SmaI
Restart
Stall
Run off
Okazaki fragments
kb 9.4 6.6 4.4 2.3 2.0 0.6 0.13
1 2 3 4 5 6 7 8
G
Pulse chase
3 kb CPD LEAD, – Pol δ
Re-priming
Scrambled
Time after chase (min) 0 5 10 20 30 0 5 10 20 30
Denaturing + SmaI
Restart
Stall
Run off
Okazaki fragments
kb 9.4 6.6 4.4 2.3 2.0 0.6 0.13
1 2 3 4 5 6 7 8 9 10

Figure S6

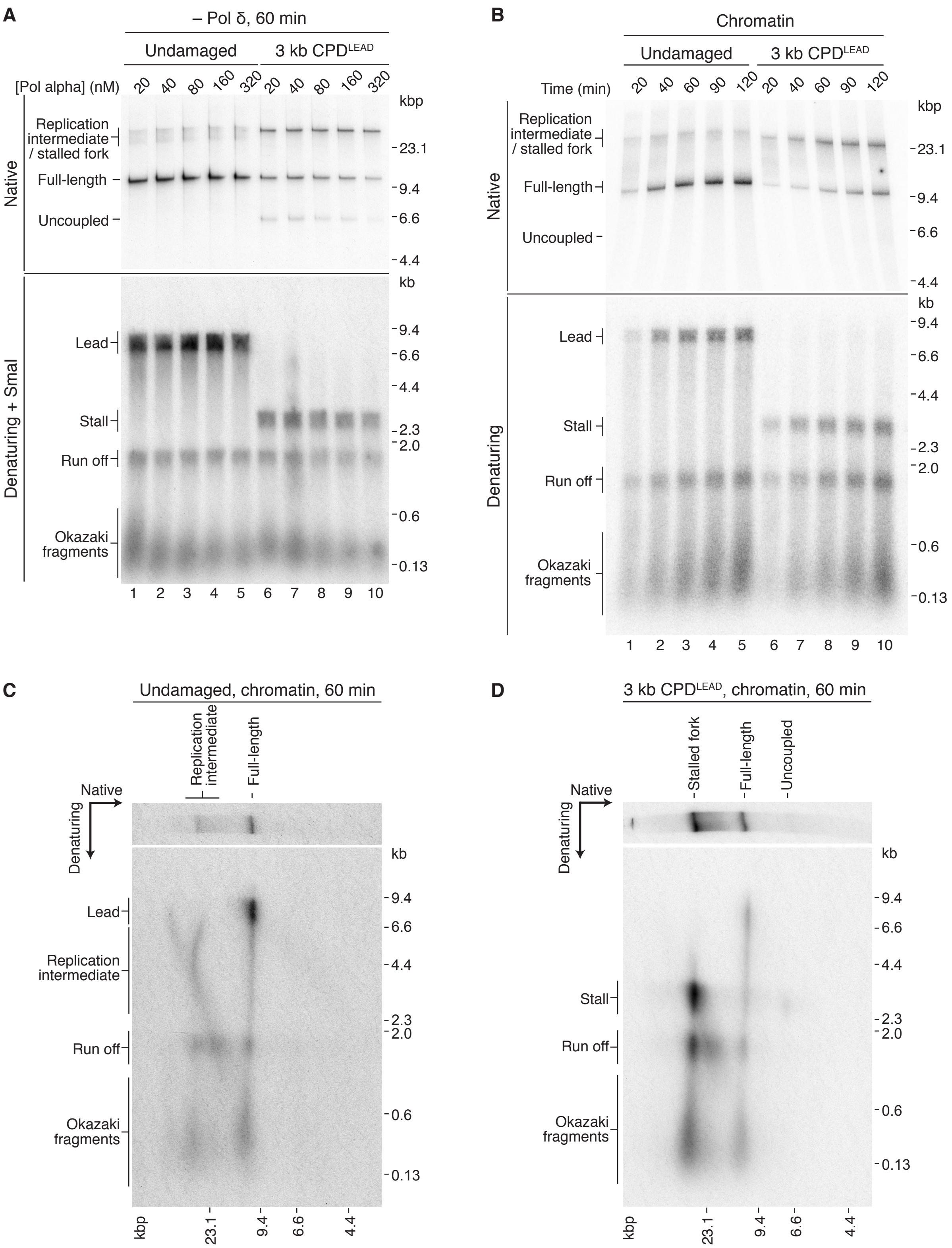

A
– Pol δ, 60 min
Undamaged
3 kb CPD^LEAD
[Pol alpha] (nM)
20 40 80 160 320 20 40 80 160 320
kbp
Native
Replication intermediate / stalled fork
Full-length
Uncoupled
23.1
9.4
6.6
4.4
kb
Denaturing + SmaI
Lead
Stall
Run off
Okazaki fragments
9.4
6.6
4.4
2.3
2.0
0.6
0.13
1 2 3 4 5 6 7 8 9 10
B
Chromatin
Undamaged
3 kb CPD^LEAD
Time (min)
20 40 60 90 120 20 40 60 90 120
kbp
Native
Replication intermediate / stalled fork
Full-length
Uncoupled
23.1
9.4
6.6
4.4
kb
Denaturing
Lead
Stall
Run off
Okazaki fragments
9.4
6.6
4.4
2.3
2.0
0.6
0.13
1 2 3 4 5 6 7 8 9 10
C
Undamaged, chromatin, 60 min
Replication intermediate
Full-length
Native
Denaturing
kb
Lead
Replication intermediate
Run off
Okazaki fragments
9.4
6.6
4.4
2.3
2.0
0.6
0.13
kbp
23.1
9.4
6.6
4.4
D
3 kb CPD^LEAD, chromatin, 60 min
Stalled fork
Full-length
Uncoupled
Native
Denaturing
kb
Stall
Run off
Okazaki fragments
9.4
6.6
4.4
2.3
2.0
0.6
0.13
kbp
23.1
9.4
6.6
4.4

Figure S7

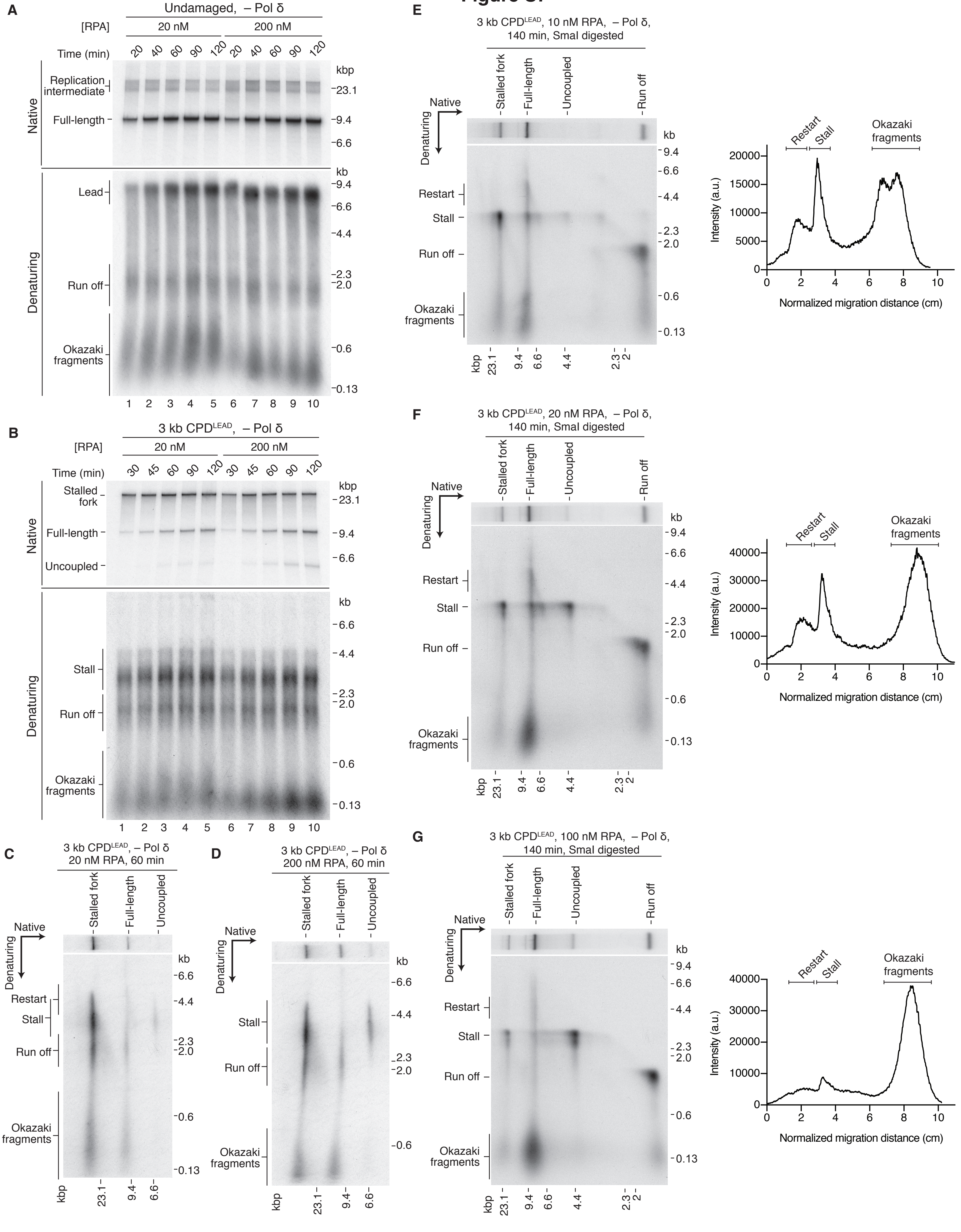

A
Undamaged, – Pol δ
[RPA]
20 nM
200 nM
Time (min)
20 40 60 90 120 20 40 60 90 120
kbp
Native
Replication intermediate
Full-length
23.1
9.4
6.6
kb
Denaturing
Lead
Run off
Okazaki fragments
9.4
6.6
4.4
2.3
2.0
0.6
0.13
1 2 3 4 5 6 7 8 9 10
B
3 kb CPD^LEAD, – Pol δ
[RPA]
20 nM
200 nM
Time (min)
30 45 60 90 120 30 45 60 90 120
Stalled fork
Full-length
Uncoupled
Stall
Run off
Okazaki fragments
1 2 3 4 5 6 7 8 9 10
C
3 kb CPD^LEAD, – Pol δ
20 nM RPA, 60 min
Stalled fork
Full-length
Uncoupled
Native
Denaturing
Restart
Stall
Run off
Okazaki fragments
kbp
23.1
9.4
6.6
D
3 kb CPD^LEAD, – Pol δ
200 nM RPA, 60 min
E
3 kb CPD^LEAD, 10 nM RPA, – Pol δ,
140 min, SmaI digested
Run off
Restart
Stall
Okazaki fragments
Intensity (a.u.)
Normalized migration distance (cm)
F
3 kb CPD^LEAD, 20 nM RPA, – Pol δ,
140 min, SmaI digested
G
3 kb CPD^LEAD, 100 nM RPA, – Pol δ,
140 min, SmaI digested

Figure S1. Related to Figure 1.
(A) Reaction scheme for standard replication reactions.
(B) Analysis of the different steps of replication template preparation. DNA products were separated through a 1% agarose gel in the presence of ethidium bromide. Lane 1: maxi prep starting material; lane 2: after nicking by Nt.BbvCI restriction endonuclease; lanes 3-8: after annealing undamaged, abasic, or CPD oligonucleotides before (lanes 3, 5, 7) and after (lanes 4, 6, 8) ligation by T4 DNA ligase; lanes 9, 11, 13: undamaged, abasic or CPD plasmids after caesium chloride gradient purification; lanes 10, 12, 14: undamaged, abasic or CPD plasmids after digestion with APE 1. BbvCI nicking and oligonucleotide ligation was performed at a site 3 kb left of ARS306 (Ori).
(C) Replication of undamaged and 6.7 kb CPD^{LAG} templates in the absence of Pol δ. In this and all subsequent experiments entirely lacking Pol δ the replication buffer contained 117 mM potassium glutamate. This potassium glutamate concentration was selected because: it supports efficient leading- and lagging-strand synthesis at rates that are comparable to those measured *in vivo* (Yeeles et al., 2017); it was not necessary to use a higher ionic strength buffer to suppress excessive Pol δ-dependent strand-displacement synthesis; we reasoned that for subsequent experiments with CPD^{LEAD} templates (Figures 3-7), the higher ionic strength (217 mM potassium glutamate) required to suppress Pol δ may not have been optimal for leading-strand re-priming.
(D) Micrococcal nuclease digest of a chromatinized undamaged template. Products were separated through a 1.5% native agarose gel and were visualized by ethidium bromide staining.
(E) FACT-dependent replication of a chromatinized undamaged template in the presence of 10 nM Pol δ.
(F) Replication of chromatinized undamaged and 6.7 kb CPD^{LAG} templates.

Figure S2. Related to Figure 2.
(A) Standard replication reaction on the 5.1 kb CPD^{LAG} template. Products were digested post-replicatively with enzymes mapping at the indicated positions from the CPD.
(B) Undigested replication reaction products for the experiment in Figure 2E.

Figure S3. Related to Figure 3.
(A and B) Replication reactions on CPD^{LEAD} templates performed in the presence (A) and absence (B) of Pol δ.
(C) Denaturing gel analysis of the replication reaction in Figure 3C following SmaI digestion.
(D) Replication reaction on the 4.5 kb CPD^{LEAD} template at 217 mM potassium glutamate showing that Pol δ does not influence uncoupled fork progression downstream of CPD^{LEAD}.

(E and F) Two-dimensional gel analysis of experiments performed on undamaged (E) and 3 kb CPD^{LEAD} (F) templates in the absence of Pol δ. Diagrams of the replication products from the undamaged template are illustrated in (E).
(G) Quantitation of pulse chase experiments on 4.5 kb CPD^{LEAD} templates as in Figure 3H, but with the respective contributions of full length and uncoupled products plotted individually (rather than summed as resolved products). Error bars represent the SEM from 4 experiments.

Figure S4. Related to Figure 4.
(A) Top: schematic of the predicted replication product of leading-strand lesion bypass by re-priming >85 nt from the CPD (3 kb CPD^{LEAD} template). The location of the BamHI, PmlI and AgeI restriction sites are illustrated. Bottom: electroeluted DNA from Figure 4B was digested with BamHI, PmlI, AgeI and DpnI and analyzed by native electrophoresis.
(B) Electroeluted DNA from Figure 4B was digested with BamHI, PmlI, AgeI and DpnI and analyzed by two-dimensional electrophoresis.
(C) Replication products from 60 min reactions containing 117 mM potassium glutamate on undamaged or 3 kb CPD^{LEAD} templates were quenched, deproteinized and digested with SmaI. Samples were then split and 3% of each was run in a native gel and processed as for standard reactions (analytical). The remaining 97% were run in separate lanes of the same native gel and the full-length bands were excised and electroeluted (preparative).
(D) Replication products from 60 min reactions containing 117 mM potassium glutamate in the presence or absence of the essential firing factor Mcm10 on 3 kb CPD^{LEAD} templates were quenched, deproteinized and digested with SmaI. The samples were then split and 2% of each was run in a native gel and processed as for standard reactions (analytical). The remaining 98% were run in separate lanes of the same gel and the full-length bands were excised and electroeluted (preparative).
(E and F) Electroeluted DNA from (D) was digested with BamHI and analyzed by two-dimensional electrophoresis. (D and F) Products generated in the absence of Mcm10 are likely due to replisome-independent template labelling catalyzed by the DNA polymerases in the reaction (Pol α, Pol δ and Pol ε).
(B and E) Full-length products (native gel) arising from leading-strand re-priming, together with their constituent stall and restart products (denaturing gel) are shown in red.

Figure S5. Related to Figure 5.
(A) Schematic illustrating the position of restriction sites for enzymes used to truncate the AhdI-linearized 4.5 kb CPD^{LEAD} templates (top), and the products of restriction enzyme digestion (bottom).
(B) Ethidium bromide stained 1% native agarose gel of digested AhdI-linearized 4.5 kb CPD^{LEAD} templates.

(C and D) Denaturing gel analysis of the experiments in Figures 5B and C respectively. Stall products from the CPD^{LEAD} templates are annotated in red.
(E) Replication reaction of an undamaged template in the presence of the 21 nt re-priming oligonucleotide or its scrambled equivalent.
(F) Comparison of replication on the 3 kb CPD^{LEAD} template with re-priming (R) oligonucleotides or scrambled equivalents (S) that anneal at different distances downstream of CPD^{LEAD} (– : no oligonucleotide). ‘Gapped full-length’ products are observed with the 1210 nt re-priming oligonucleotide. These products migrate in a distinct position below full-length products due to the size of the ssDNA region between the CPD and re-priming oligonucleotide binding site.
(G) Denaturing gel analysis of the pulse chase experiment in Figure 5F.

Figure S6. Related to Figure 6.
(A) Effect of Pol α concentration on replication of undamaged and 3 kb CPD^{LEAD} templates.
(B) Replication reactions on chromatinized undamaged and 3 kb CPD^{LEAD} templates.
(C and D) Two-dimensional gel analysis of experiments performed on chromatinized undamaged (C) and 3 kb CPD^{LEAD} (D) templates.

Figure S7. Related to Figure 6.
(A and B) Replication reactions performed on undamaged (A) and 3 kb CPD^{LEAD} templates (B) at 20 nM and 200 nM RPA.
(C and D) Two-dimensional gel analysis of experiments performed at 20 nM RPA (C) and 200 nM RPA (D) on the 3 kb CPD^{LEAD} template. Replication products were not digested.
(E-G) Two-dimensional gel analysis of experiments performed with 20 nM Pol α at 10 nM (E), 20 nM (F) and 100 nM (G) RPA on the 3 kb CPD^{LEAD} template. Replication products were digested with SmaI. Lane profiles of the denaturing gel showing the constituents of the full-length products (native) are shown next to each gel.

Table S1. Oligonucleotides used in this study. Related to the STAR Methods (Key Resources Table).

**Oligonucleotides**		
For cloning PstI/BamHI cassette (top): PB_Top: GATCCGCAGTACCAACCTCAGCACTTAAGTCCTCAGCGCAGTACCAACTGCA	This paper	N/A
For cloning PstI/BamHI cassette (bottom): PB_Bottom: GTTGGTACTGCGCTGAGGACTTAAGTGCTGAGGTTGGTACTGCG	This paper	N/A
For cloning SphI/SpeI cassette (top): SS_Top: CTAGTGCAGTACCAACCTCAGCACTTAAGTCCTCAGCGCAGTACCAAGCATG	This paper	N/A
For cloning SphI/SpeI cassette (bottom): SS_Bottom: CTTGGTACTGCGCTGAGGACTTAAGTGCTGAGGTTGGTACTGCA	This paper	N/A
AflII_undamaged: 5'-phos-TCAGCACTTAAGTCC	This paper	N/A
AflII_idSp: 5'-phos-TCAGCACT-/idSp/-AAGTCC	This paper	N/A
AflII_CPD: 5'-phos-TCAGCAC-/CPD/-AAGTCC	This paper	N/A
AflII_competitor: GTTGGTACTGCGCTGAGGACTTAAGTGCTGAGGTTGGTACTGCG	This paper	N/A
SapI_Btn_1: 5'-phos-GCTATGTGGTAGGAAGTGAG-biotin-TEG-3'	This paper	N/A
SapI_Btn_2: 5'-biotin-CTCACTTCCTACCACAT-3'	This paper	N/A
For artificial restart assays: 21 nt re-priming: GGTTGGTACTGCGG	This paper	N/A
For artificial restart assays: 21 nt scrambled: GGTGATCGTTCGGG	This paper	N/A
For artificial restart assays: 265 nt re-priming: CTGGTTTCCGCCGT	This paper	N/A
For artificial restart assays: 265 nt scrambled: GTTCGCCTTGCCGT	This paper	N/A
For artificial restart assays: 1210 nt re-priming: GGCGTCACTACCAC	This paper	N/A
For artificial restart assays: 1210 nt scrambled: GCCCAGTACTGCAC	This paper	N/A
JY180: primase assay: GAATAATGGAAGGGTTAGAACCTACCAT	This paper	N/A
