# Supplementary material for: The Initial Response of a Eukaryotic Replisome to DNA Damage
Source: Mol Cell. 2018 Jun 21;70(6):1067–1080.e12. doi: 10.1016/j.molcel.2018.04.022 (PMC6024075; doi:10.1016/j.molcel.2018.04.022)
Supplement: Document S2. Article plus Supplemental Information [file mmc2.pdf]

Article

Molecular Cell

The Initial Response of a Eukaryotic Replisome to DNA Damage

Graphical Abstract

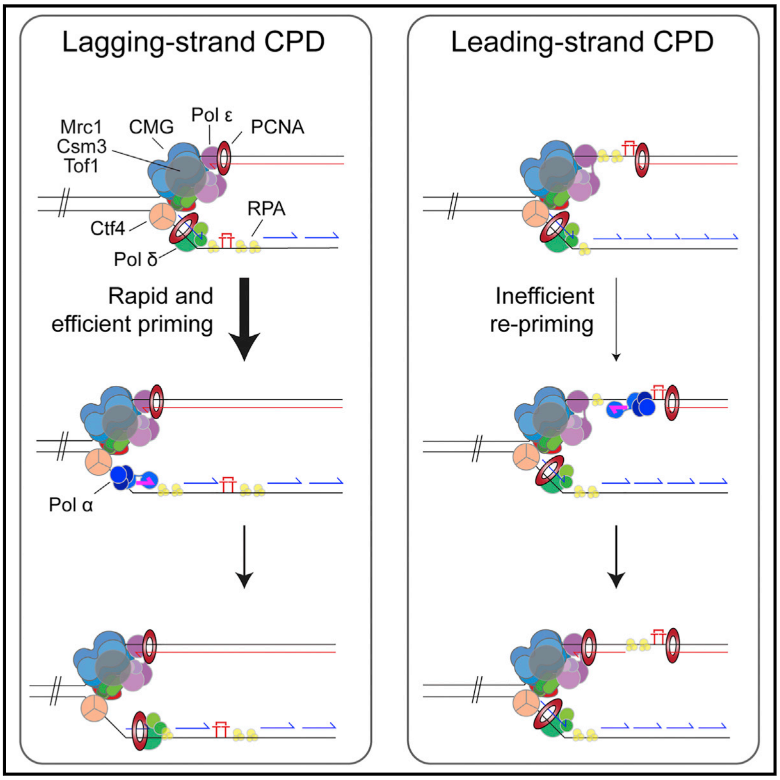

Authors

Martin R.G. Taylor, Joseph T.P. Yeeles

Correspondence

jyeeles@mrc-lmb.cam.ac.uk

In Brief

To study the earliest events following DNA polymerase stalling during replication, Taylor and Yeeles reconstituted collisions between a yeast replisome assembled with purified proteins and template DNA damage. Surprisingly, re-priming of leading-strand synthesis beyond damage is inefficient but is promoted by RPA depletion, whereas lagging-strand priming occurs robustly after damage.

Highlights

- Reconstitution of collisions between a eukaryotic replisome and DNA damage
- Leading-strand damage specifically causes fork stalling and uncoupling
- The eukaryotic replisome can re-initiate leading-strands downstream of DNA damage
- Multiple mechanistic differences exist between leading- and lagging-strand priming

Taylor & Yeeles, 2018, Molecular Cell *70*, 1067–1080
June 21, 2018 © 2018 MRC Laboratory of Molecular Biology.
Published by Elsevier Inc.
https://doi.org/10.1016/j.molcel.2018.04.022

CellPress

CellPress

The Initial Response of a Eukaryotic Replisome to DNA Damage

Martin R.G. Taylor[1] and Joseph T.P. Yeeles[1,2,*]
[1]Division of Protein and Nucleic Acid Chemistry, Medical Research Council Laboratory of Molecular Biology, Francis Crick Avenue, Cambridge CB2 0QH, UK
[2]Lead Contact
*Correspondence: jyeeles@mrc-lmb.cam.ac.uk
https://doi.org/10.1016/j.molcel.2018.04.022

SUMMARY

The replisome must overcome DNA damage to ensure complete chromosome replication. Here, we describe the earliest events in this process by reconstituting collisions between a eukaryotic replisome, assembled with purified proteins, and DNA damage. Lagging-strand lesions are bypassed without delay, leaving daughter-strand gaps roughly the size of an Okazaki fragment. In contrast, leading-strand polymerase stalling significantly impacts replication fork progression. We reveal that the core replisome itself can bypass leading-strand damage by re-priming synthesis beyond it. Surprisingly, this restart activity is rare, mainly due to inefficient leading-strand re-priming, rather than single-stranded DNA exposure or primer extension. We find several unanticipated mechanistic distinctions between leading- and lagging-strand priming that we propose control the replisome's initial response to DNA damage. Notably, leading-strand restart was specifically stimulated by RPA depletion, which can occur under conditions of replication stress. Our results have implications for pathway choice at stalled forks and priming at DNA replication origins.

INTRODUCTION

Accurate and efficient DNA replication is vital to ensure faithful and timely transmission of genetic information. This task is accomplished by a complex and highly regulated molecular machine known as the replisome. Replisomes frequently encounter a wide variety of obstacles to their progression, including template DNA damage, DNA secondary structures, and the transcription machinery (Yeeles et al., 2013; Zeman and Cimprich, 2014). Considerable progress has been made in delineating pathways that sustain replication fork progression following DNA damage, which include translesion synthesis (TLS), homologous recombination, replication fork reversal, the Fanconi anemia pathway, and the DNA replication checkpoint. However, much less is known about the initial response of the core eukaryotic replisome upon encountering a polymerase-stalling lesion. This response is likely to be of fundamental importance for maintaining genomic stability, since it will be instrumental in determining how and when the damage is ultimately bypassed.

Early studies in *Escherichia coli* found that, following UV irradiation of nucleotide excision repair (NER)-defective cells, replication continued and nascent DNA was synthesized as small fragments interspersed with single-stranded DNA (ssDNA) gaps (Iyer and Rupp, 1971; Rupp and Howard-Flanders, 1968). Based on these observations of discontinuous replication, it was proposed that synthesis could be reinitiated downstream of lesions in both template strands. The inherently discontinuous nature of lagging-strand synthesis provided an obvious means to bypass damage in this strand, and this has been observed with purified *E. coli* proteins (McInerney and O'Donnell, 2004). However, leading-strand synthesis is normally continuous on unmodified templates, and for many years, leading-strand priming outside the origin of replication was considered unlikely (Lehmann and Fuchs, 2006). Mechanistic insights into replisome-mediated leading-strand restart came when it was shown that the *E. coli* replisome has the inherent capacity to "skip" over multiple leading-strand lesions via re-priming (Yeeles and Marians, 2011, 2013).

Experiments in eukaryotic cells also found that replication was discontinuous following UV irradiation (Lehmann, 1972). More recently, direct visualization by electron microscopy of replication intermediates from NER-deficient yeast cells treated with UV revealed the presence of ssDNA gaps in both daughter strands, providing strong evidence that leading-strand damage can be bypassed by re-priming in eukaryotes (Lopes et al., 2006). Additional indirect support for leading-strand re-priming in yeast comes from observations that (1) ubiquitin-mediated DNA damage tolerance (DDT) can be delayed until G2/M without significant loss of viability (Daigaku et al., 2010; Karras and Jentsch, 2010), implying that replication forks may bypass damage in both strands to leave gapped substrates for post-replicative repair; and (2) impaired re-priming and ssDNA gap formation has been hypothesized to underlie the template-switching defects exhibited by certain DNA polymerase alpha-primase (Pol α) and Ctf4 yeast mutants (Fumasoni et al., 2015). However, while these studies have provided support for re-priming under conditions where many forks stall due to high lesion density, it remains unexplored whether these responses are distinct from those of replisomes encountering isolated lesions during an unperturbed S phase.

In apparent contradiction to models invoking efficient re-priming, there is also considerable evidence that template DNA

This is an open access article under the CC BY license (http://creativecommons.org/licenses/by/4.0/).

damage inhibits eukaryotic replication forks. For example, stalling and slowing of forks can be detected *in vivo* following treatment of cells with DNA-damaging agents (Lopes et al., 2006; Neelsen and Lopes, 2015; Tercero and Diffley, 2001). Because efficient replicative bypass of a lagging-strand obstacle has been observed in *Xenopus* cell-free extracts (Fu et al., 2011), these results collectively imply that leading-strand polymerase stalling may specifically impede replisome progression, although this has not been demonstrated directly. Consistent with this notion, long ssDNA regions of up to 3 kb have been detected at replication forks isolated from UV-irradiated yeast cells (Lopes et al., 2006). This suggests that leading-strand polymerase stalling at UV-induced lesions may drive helicase-polymerase uncoupling, with template unwinding and lagging-strand synthesis continuing beyond the damage. It is unclear why some replisomes exposed extensive ssDNA without resuming leading-strand synthesis, especially given that the yeast primase, Pol α, has been shown to catalyze robust leading-strand priming on model fork structures (Georgescu et al., 2015),

More generally, how the *in vivo* observations of both re-priming and fork stalling in yeast can be accommodated within a single model remains to be determined, in large part because a mechanistic basis for re-priming has not been established. The significant challenges of specifically and quantitatively detecting leading-strand re-priming products *in vivo*, together with the presence of competing pathways that target stalled forks, mean that cellular studies have been unable to address this issue. Furthermore, collisions between a eukaryotic replisome and site-specific DNA damage have not been reconstituted with purified proteins. Because substantial architectural and mechanistic differences exist between prokaryotic and eukaryotic replisomes, it remains to be seen whether the response of the *E. coli* replisome to template damage is generalizable. Consequently, we do not know how the core eukaryotic replisome responds to damage in either template strand. It is also not known whether leading-strand re-priming activity is inherent to the eukaryotic replisome, the mechanism(s) by which it might operate, how efficiently it occurs, or how it might be regulated. In this study, we sought to address these outstanding questions by analyzing the response of a reconstituted core eukaryotic replisome to site-specific DNA damage.

RESULTS

A System to Monitor Unidirectional Collisions between a Eukaryotic Replisome and Site-Specific DNA Damage

To monitor the outcomes of collisions between replisomes and DNA damage in either the leading- or lagging-strand templates, we adapted a system we recently described (Yeeles et al., 2015, 2017), in which a replisome is assembled with purified budding yeast proteins. Briefly, loaded MCM double hexamers are activated via a regulated pathway involving multiple "firing factors" to form two CMG (Cdc45-MCM-GINS) helicases, around which the replisome is assembled (Figure S1A). These replisomes perform complete leading- and lagging-strand synthesis at the *in vivo* rate, DNA polymerase epsilon (Pol ε) catalyzes the bulk of leading-strand synthesis in conjunction with PCNA, DNA polymerase delta (Pol δ) is required for complete lagging-strand synthesis, and Mrc1 and Csm3/Tof1 are critical for maximum replication rates (Yeeles et al., 2017).

We integrated a cyclobutane pyrimidine dimer (CPD), which is one of the primary lesions generated by UV irradiation, at a specific location in plasmid DNA (Figure S1B) and developed an assay to monitor unidirectional fork progression. Plasmid templates were linearized asymmetrically with respect to ARS306 (Ori), such that origin-dependent replication should generate two distinct replication arms (Figure 1A). Replication of undamaged templates linearized with AhdI or BamHI produced leading-strand products of the predicted sizes (Figures 1A and 1B). Long leading strands from both templates migrated as prominent but diffuse bands, whereas leading strands from the shorter replicons were less well defined, suggesting leading strands were heterogeneous in length. Post-replication cleavage with the restriction endonuclease SmaI, which maps ~100 bp from the origin (Figure 1A), removed this heterogeneity (Figure 1B). This indicated that replication was highly origin specific and that heterogeneity arose due to variability in the exact location at which leading-strand synthesis was initiated.

A Lagging-Strand Template CPD Is Efficiently Bypassed by the Replisome

We first linearized a CPD-containing plasmid with AleI to place the CPD 6.7 kb from ARS306 in the lagging-strand template (CPD^{LAG}) (Figure 1C). The replisome efficiently bypassed CPD^{LAG}, generating full-length duplex products of 9.7 kilobase pairs (kbp) with a similar efficiency to those synthesized from an undamaged template (Figure 1D, native). Inspection of a denaturing gel revealed that these products comprised Okazaki fragments and 8.4 kb leading-strands from rightward moving forks (Figure 1D, denaturing). Consistent with the data in Figure 1B, short leading strands (predicted to be 1.3 kb) from leftward-moving forks (run off) migrated as a less well-defined species that was partially obscured by Okazaki fragments. To examine the kinetics of CPD^{LAG} bypass with greater temporal resolution, we performed pulse-chase experiments. Figure 1F shows there was no significant delay in leading-strand synthesis past the 5.1 kb CPD^{LAG} (Figure 1E), as products longer than 5.1 kb were produced at equivalent time points to an undamaged template (compare lanes 3 and 9 and lanes 4 and 10). Moreover, quantitation of pulse-chase experiments (Figure 1G) illustrates that data from time points taken before (3 and 4 min) and after (5 and 6 min) the replisome had encountered CPD^{LAG} at 5.1 kb fit equally well to a linear regression, supporting the conclusion that CPD^{LAG} had little impact on leading-strand synthesis rate.

Lagging-strand synthesis has been observed in the absence of Pol δ in multiple *in vitro* studies (Devbhandari et al., 2017; Yeeles et al., 2015, 2017), with Pol α proposed to perform the bulk of synthesis under these conditions (Georgescu et al., 2015). Therefore, to assess if the identity of the polymerase encountering CPD^{LAG} influenced replisome progression, we performed a reaction in the absence of Pol δ (Figure S1C). Products from CPD^{LAG} and undamaged templates were again almost indistinguishable, indicating efficient bypass of CPD^{LAG} regardless of which polymerase encountered the lesion. In the absence of Pol δ, products migrated as more clearly defined bands in the native gel, and in the denaturing gel, run off products were better

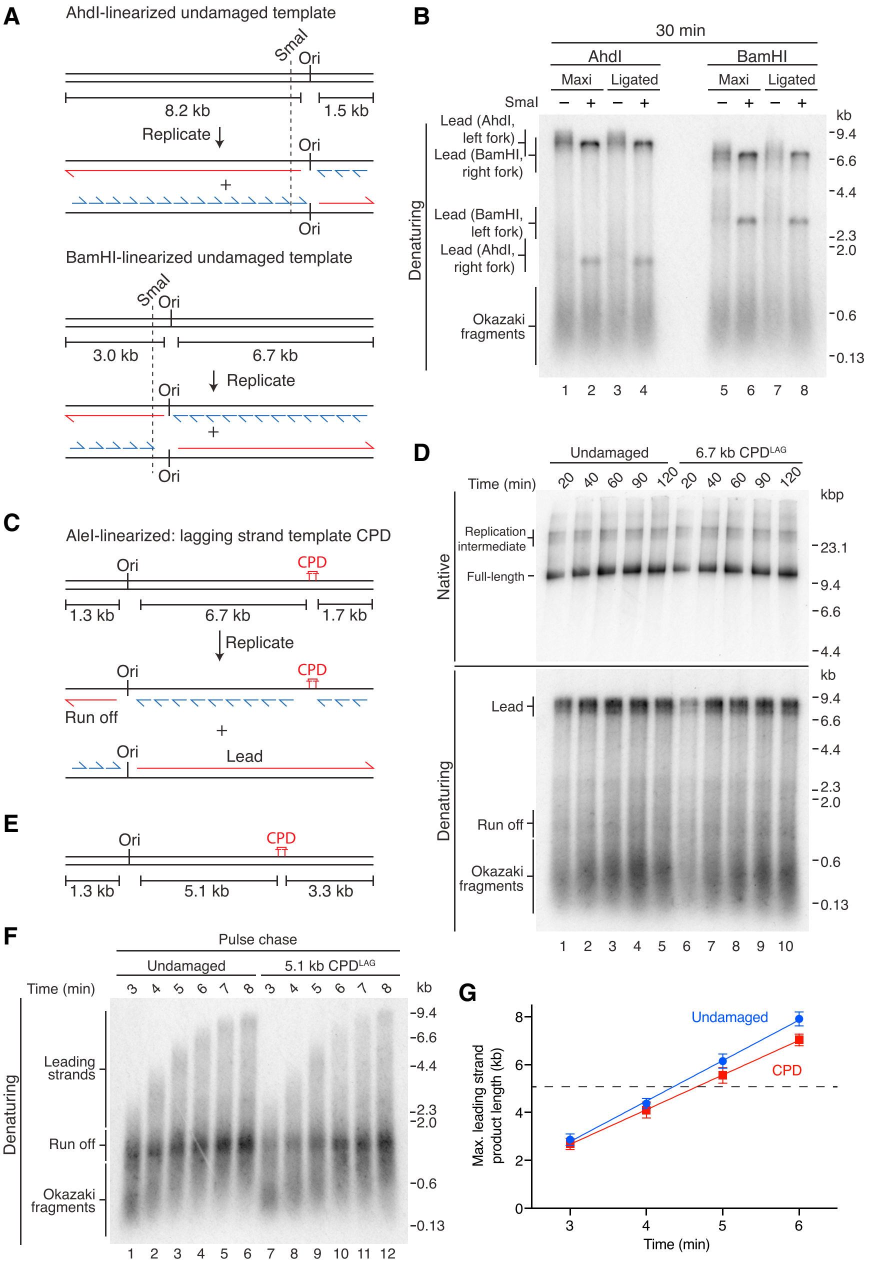

Figure 1. The Replisome Rapidly and Efficiently Bypasses a Lagging-Strand CPD

(A) Schematic of AhdI- and BamHI-linearized undamaged templates and the predicted replication products. In this and all subsequent figures; red: leading-strands; blue: lagging-strands; the position of the ARS306 origin of replication is marked, Ori. The location of the SmaI restriction site is indicated.

(B) Standard replication reactions performed on the templates illustrated in (A). Unless stated otherwise, this and all subsequent standard replication assays contained 217 mM potassium glutamate. Templates were prepared by linearizing maxiprep DNA (Maxi) or undamaged plasmids prepared using the same method used to generate CPD containing plasmids (Ligated).

(C) Schematic of the 6.7 kb CPD^{LAG} template and the predicted replication products of lagging-strand lesion bypass.

(D) Replication reaction comparing undamaged and 6.7 kb CPD^{LAG} templates.

(E) Schematic of the 5.1 kb CPD^{LAG} template.

(F) Pulse-chase experiment on undamaged and 5.1 kb CPD^{LAG} templates. The chase was added at 2 min 50 s.

(G) Quantitation of pulse-chase experiments performed as in (F). Error bars represent the SEM from four experiments. Data were fit to a linear regression. Dashed line indicates the distance from Ori to CPD^{LAG} (5.1 kb).

resolved from Okazaki fragments (compare Figures 1D [+Pol δ] and S1C [−Pol δ]). The less well-defined products in the presence of Pol δ are likely due to limited Pol δ-dependent strand-displacement synthesis (Devbhandari et al., 2017).

To determine whether CPD^{LAG} bypass occurred efficiently on chromatinized templates, we established conditions for FACT-dependent chromatin replication (Kurat et al., 2017) in our system (Figures S1D and S1E). Interestingly, both leading-strand products migrated as sharp bands in a denaturing gel, indicating that nucleosomes restricted heterogeneity in product length. Comparison of products from undamaged and CPD^{LAG} templates revealed that replisome progression past the lesion occurred efficiently in the context of chromatin (Figure S1F).

Daughter-Strand Gaps Are Generated during Bypass of CPD^{LAG}

Bypass of CPD^{LAG} should generate daughter-strand gaps due to inhibition of the lagging-strand polymerase by the lesion (Figure 1C). To test for their presence, we treated reaction products with a panel of restriction endonucleases that mapped to the region immediately upstream of CPD^{LAG} with respect to the direction of fork progression (Figure 2A). Full-length products should be resistant to cleavage if they contain a single-strand gap at the location of the restriction site. Products from an undamaged template were fully digested by all four enzymes (Figure 2B). In contrast, a proportion of the full-length products from the CPD^{LAG} template were resistant to digestion (Figure 2B, native, lanes 7–10). Because leading-strand products were fully digested (Figure 2B, denaturing) and enzyme-resistant material was composed almost exclusively of Okazaki fragments and run off (Figure 2D), we conclude that undigested full-length products represent lagging-strand daughters with ssDNA gaps. The proportion of resistant material decreased as the enzyme recognition site was moved further from CPD^{LAG}, such that ssDNA gaps rarely extended more than 374 bases from the damage (Figures 2B, 2C, and S2A). Given that lagging-strand products in these experiments

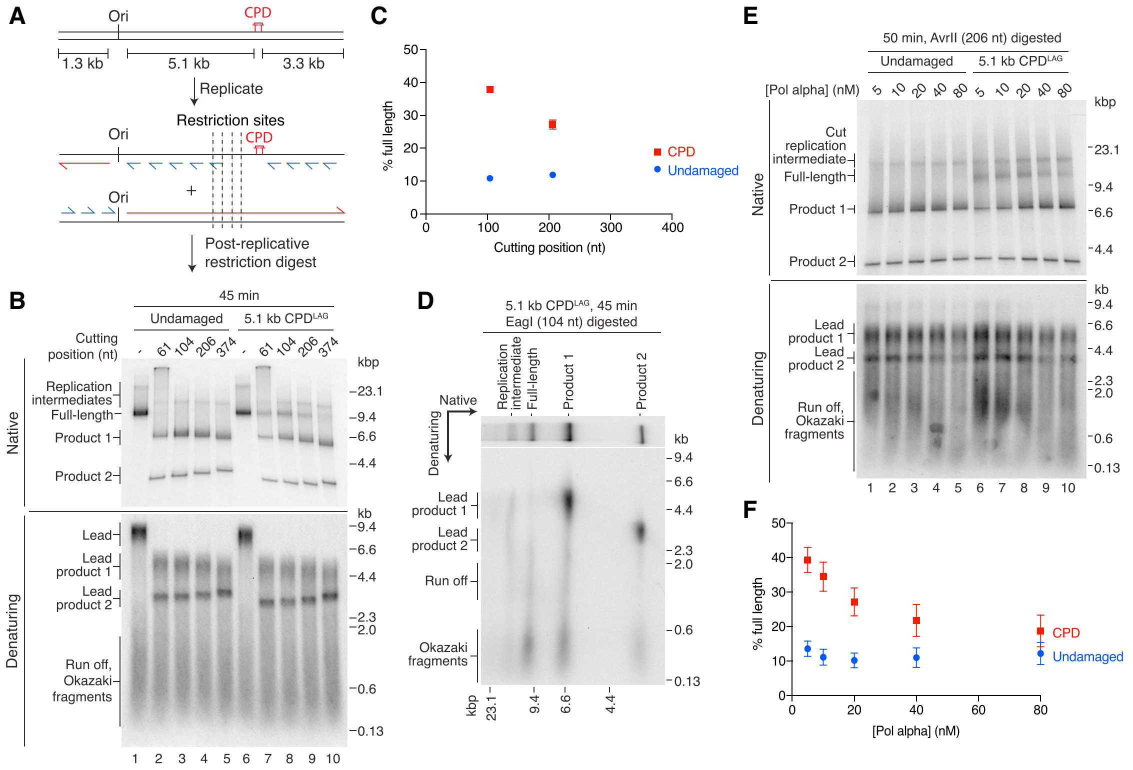

Figure 2. Daughter-Strand Gaps Are Generated during Bypass of CPD^{LAG}

(A) Schematic showing the location of restriction sites relative to the 5.1 kb CPD^{LAG}.

(B) Replication products from undamaged and 5.1 kb CPD^{LAG} templates were digested post-replicatively. The distances from enzyme recognition sequences to the TT dinucleotide that is crosslinked in CPD templates are shown (cutting position).

(C) Quantitation of full-length products from experiments performed as in (B). Error bars represent the SEM from three experiments.

(D) Two-dimensional gel of post-replicatively digested CPD^{LAG} products. Full-length products in the native gel are composed of run off products and Okazaki fragments.

(E) Effect of Pol α concentration on daughter-strand gap size. Replication products were digested post-replicatively with AvrII.

(F) Quantitation of full-length products from Pol α titrations performed as in (E). Error bars represent the SEM from three experiments.

were ∼200–600 nt in length, the data indicate that CPD^{LAG} inhibits the synthesis of a single Okazaki fragment. We therefore examined ssDNA gap size over a range of Pol α concentrations, as Pol α concentration modulates priming frequency (Yeeles et al., 2017). Accordingly, both the proportion of enzyme-resistant material and Okazaki fragment length decreased with increasing Pol α concentration (Figures 2E, 2F, and S2B), demonstrating that priming frequency controls ssDNA gap size during bypass of a lagging-strand CPD.

Template Unwinding and Lagging-Strand Synthesis Continue Downstream of CPD^{LEAD}

We next interrogated the response of a replisome to a leading-strand CPD (CPD^{LEAD}) by linearizing plasmids with AhdI (Figure 3A). We observed three distinct replication products in native gels. At early time points, most products migrated as a high-molecular-weight species (Figure 3B, native, lane 6, stalled fork), comprised of stalled leading strands (3 kb, stall), leading strands from the rightward fork (1.5 kb, run off), and Okazaki fragments (Figure 3B, denaturing, lane 6). Full-length products accumulated as the reaction progressed, as did a lower molecular species (uncoupled) (Figure 3B, native, lanes 7–10). Surprisingly, leading-strand restart products (up to 5.2 kb for a reinitiation event close to the 3 kb CPD^{LEAD}, Figure 3A), were not detectable in denaturing gels, even after heterogeneity was clarified by SmaI digestion (Figure S3A, denaturing, lane 2). We then compared replication in the presence and absence of Pol δ, reasoning that Pol δ strand-displacement synthesis activity may have obscured rare restart events. Although Pol δ omission again resulted in better-defined products, the principal reaction features were largely unaffected: full-length and uncoupled products were generated with similar efficiency and kinetics, and restart products were not readily observed (Figure S3D). Likewise, reducing the ionic strength of reactions lacking Pol δ (from 217 mM to 117 mM potassium glutamate), had minimal effect on the formation of full-length, restart, and uncoupled products (Figures 3C, S3B, and S3C), even after SmaI digestion, demonstrating that restart remained inefficient over a range of buffer conditions.

To determine the architecture of reaction products, we analyzed their nascent-strand composition by migration through a second denaturing dimension. At a 60-min time point, full-length material from an undamaged template (Figure S3E) contained complete leading strands from leftward (8.2 kb, lead) and

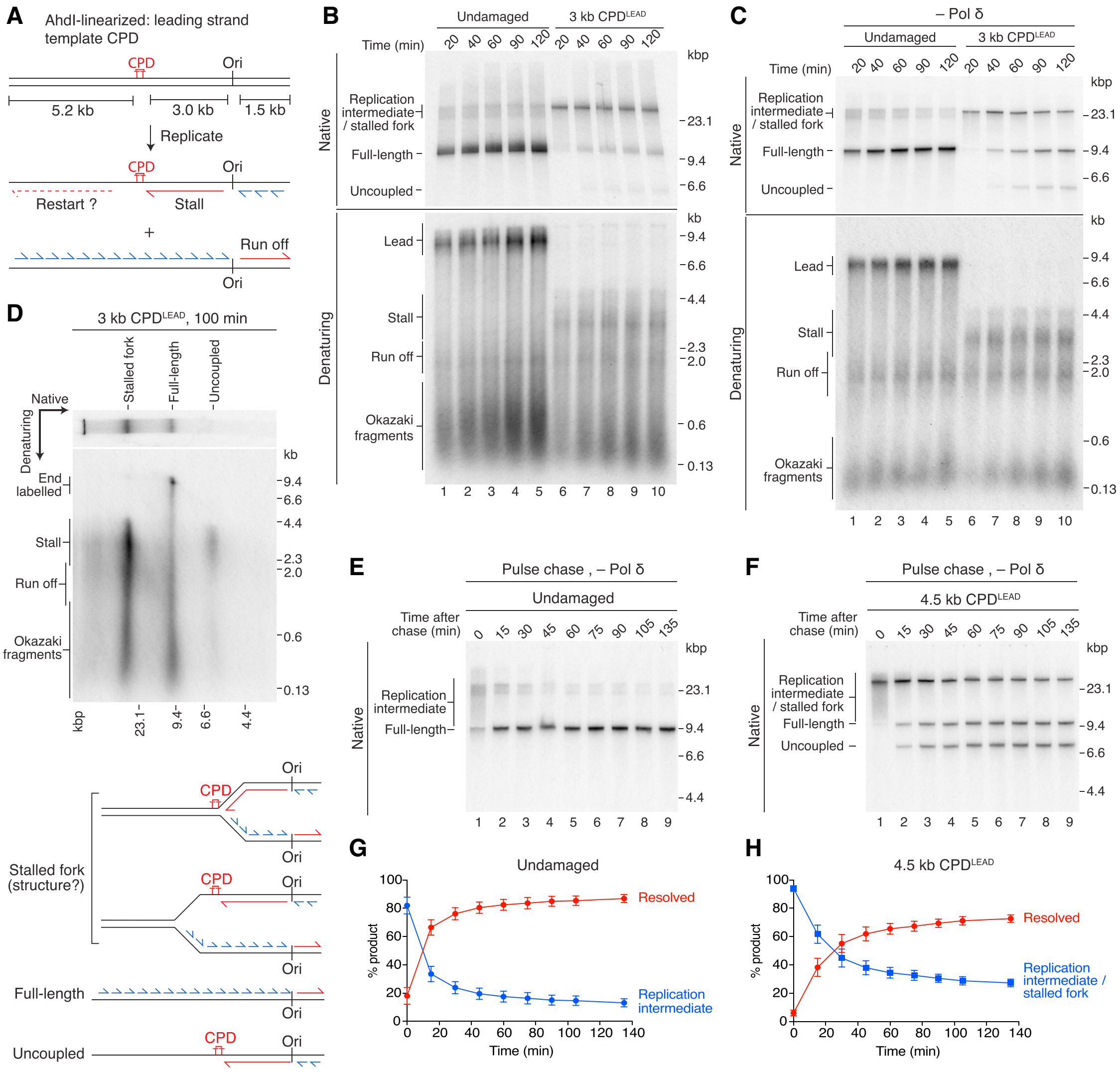

Figure 3. Response of the Replisome to a Leading-Strand CPD

(A) Schematic showing the 3 kb CPD^{LEAD} template and the predicted replication products of leading-strand lesion bypass by re-priming. In this and all subsequent figures the putative restart product is shown as a dashed red line.

(B and C) Comparison of replication products from undamaged and 3 kb CPD^{LEAD} templates in the presence (B) and absence of Pol δ (C). (C) In this and all subsequent experiments entirely lacking Pol δ, the reaction buffer contained 117 mM potassium glutamate.

(D) Two-dimensional gel of a replication reaction performed on the 3 kb CPD^{LEAD} template (top), together with a schematic of the products generated (bottom).

(E and F) Pulse-chase experiments on undamaged (E) and 4.5 kb CPD^{LEAD} templates (F). The chase was added 14 min 50 s after replication was initiated.

(G and H) Quantitation of pulse-chase experiments on undamaged (G) and 4.5 kb CPD^{LEAD} templates (H) performed as in (E) and (F). "Resolved" is the sum of full-length and uncoupled products. Error bars represent the SEM from four experiments.

rightward (1.5 kb, run off) moving forks and Okazaki fragments, showing these represented completely duplicated replicons. The higher-molecular-weight species contained run off products, Okazaki fragments, and incomplete leading strands of varying lengths, indicating that they were replication intermediates (Figure S3E, replication intermediate). For the CPD^{LEAD} template, the higher-molecular-weight species (Figures 3D and S3F, native, stalled fork) contained Okazaki fragments and leading-strands from both forks, suggesting it represents a Y-shaped stalled fork. Full-length products predominantly contained run off and Okazaki fragments (Figures 3D and S3F), however a smear of longer products up to 9.7 kb in length were also produced (end labeled). The longer products were likely the result of replisome-independent template labeling and were more abundant in the presence of Pol δ (compare Figures 3D and S3F, full length). Importantly, however, distinct stalled (3 kb) or restarted leftward leading-strands (up to 5.2 kb) were undetectable, either in the presence (Figure 3D) or absence (Figure S3F) of Pol δ, suggesting that restart was either highly inefficient or absent. Finally, the lower-molecular-weight product (Figures 3D and S3F, native, uncoupled) contained predominantly leading-strand stall products and some lagging strands (Figures 3D and S3F, denaturing), likely

from the rightward fork. We hypothesized that full-length and uncoupled products represented reciprocal daughters generated by continued CMG-dependent template unwinding and lagging-strand synthesis in the absence of leading-strand restart. Moving CPD^{LEAD} further from the origin reduced the mobility of uncoupled products (Figures S3A and S3B), consistent with an increase in molecular weight resulting from the reduced region of ssDNA.

To monitor the extent and kinetics of uncoupled fork progression, we performed pulse-chase experiments (Figures 3E–3H). Because Pol δ did not significantly influence replisome progression downstream of CPD^{LEAD}, it was omitted to avoid complications from strand displacement synthesis during long incubations in the presence of elevated deoxynucleotides (dNTPs) required for the chase. Compared to an undamaged template, replication forks were almost universally delayed for several min by CPD^{LEAD} (Figures 3E and 3F, compare lane 1). Full-length and uncoupled products accumulated synchronously and in equal proportions, confirming that they represent reciprocal daughters (Figures 3F and S3G). These products accumulated more slowly than full-length products from an undamaged template (compare Figures 3G and 3H, resolved), indicating that replisome progression was delayed by CPD^{LEAD}.

Evidence for Daughter-Strand Gaps Downstream of CPD^{LEAD}

Undetectable leading-strand restart was unexpected. If restart occurred inefficiently, then products might have been obscured by background nucleotide incorporation, such as that from replisome-independent template labeling. Re-priming will generate ssDNA gaps between the stalled nascent leading strand and the 5′ end of the putative restart product; as such, the corresponding daughter molecules should be insensitive to restriction enzymes mapping to this region (Figure 4A), as was the case for CPD^{LAG} bypass (Figure 2). We sought to exploit this insensitivity to enrich for daughter molecules formed by leading-strand restart within the full-length population (Figure 4A). To do so, SmaI-digested full-length products were isolated from a native gel by electroelution (Figure 4B), and the recovered DNA was treated with BamHI, which maps 20 nt downstream of CPD^{LEAD}, and DpnI to digest un-replicated methylated template DNA that may have been non-specifically labeled. Full-length products persisted after BamHI and DpnI digestion (Figure 4C). Crucially, when analyzed in the denaturing dimension, these enzyme-resistant products contained stalled leading strands (stall) and longer leading strands of ~5 kb (restart) (Figure 4D), suggesting they resulted from reinitiation by re-priming. Full-length products also comprised some Okazaki fragments, indicating that for reasons discussed below, some lagging-strand daughters were insensitive to BamHI. These products, but not restarted leading-strands, were almost completely depleted by two additional restriction enzymes mapping only slightly further downstream of CPD^{LEAD} (Figures S4A and S4B), confirming that lagging-strand synthesis efficiently resumed downstream of CPD^{LEAD} despite the BamHI insensitivity. Moreover, this result illustrates that leading-strand reinitiation can occur at distances of greater than 85 bases downstream of the lesion.

To examine whether the replisome could perform leading-strand restart over a range of buffer conditions, we performed equivalent experiments at lower ionic strength in the presence of Pol δ, reasoning that more extensive strand-displacement synthesis under these conditions should not adversely affect isolated leading-strand replication products. Full-length material again comprised restart products visible in the denaturing dimension (Figures 4E and S4E). A greater proportion of lagging-strand products were digested by BamHI under these conditions. The BamHI insensitivity observed at higher salt may therefore have resulted from reduced strand displacement at this position, although the mechanistic basis for these observations remains to be determined.

Isolation of full-length products from an undamaged template followed by BamHI digestion confirmed that restart products were dependent on CPD^{LEAD} (Figures 4F and S4C). We also validated that restart products were dependent on replication initiation, as full-length products containing "stall" and "restart" were not observed in the absence of the essential firing factor Mcm10 (Figures S4D–S4F). Full-length material in the absence of Mcm10 (Figure S4D) likely arose due to replisome-independent template labeling. Taken together, these results reveal that the eukaryotic replisome alone has the inherent ability to bypass a leading-strand CPD by re-priming; however, it does so inefficiently under our standard experimental conditions.

Priming Is the Main Limiting Step in Leading-Strand Restart

We next sought to understand why leading-strand restart was inefficient. To determine whether all replisomes generated a ssDNA region on the leading-strand template, we modified the length of the template downstream of CPD^{LEAD} (Figures 5A, S5A, and S5B). At an early time point (20 min), replication of templates with 104-bp and 376-bp regions downstream of CPD^{LEAD} produced products very similar to those synthesized from equivalent undamaged templates in both the presence (Figures 5B and S5C) and absence (Figures 5C and S5D) of Pol δ. The major reaction species migrated as a full-length product, indicating that CMG unwound at least 350 bp beyond CPD^{LEAD} to reach the end of these templates. Distinct uncoupled products were not observed on these templates, presumably because the CPD distal ssDNA region was too short to sufficiently alter their mobility. Lengthening the CPD distal region to 973 bp resulted in a significant fraction of replicated products from the CPD^{LEAD} template migrating as a stalled fork (Figure 5B, lane 9 and Figure 5C, lane 8). This indicated that many replisomes had slowed and/or stalled such that they had progressed less than 1,000 bp downstream of CPD^{LEAD}. When replisomes did reach the end of this template, full-length and uncoupled products were synthesized, indicating that lagging-strand synthesis had continued downstream of CPD^{LEAD} in the absence of leading-strand restart. Collectively, these data demonstrate that the majority of replisomes exposed a region of ssDNA on the leading-strand template, suggesting that continued template unwinding by CMG is not a significant limiting step in leading-strand restart.

We hypothesized that Pol α might not efficiently prime the leading-strand template beyond CPD^{LEAD}. If this were the main limiting step for restart, then it might be circumvented by co-incubating reactions with oligonucleotides complementary to the leading-strand template downstream of CPD^{LEAD}. Indeed,

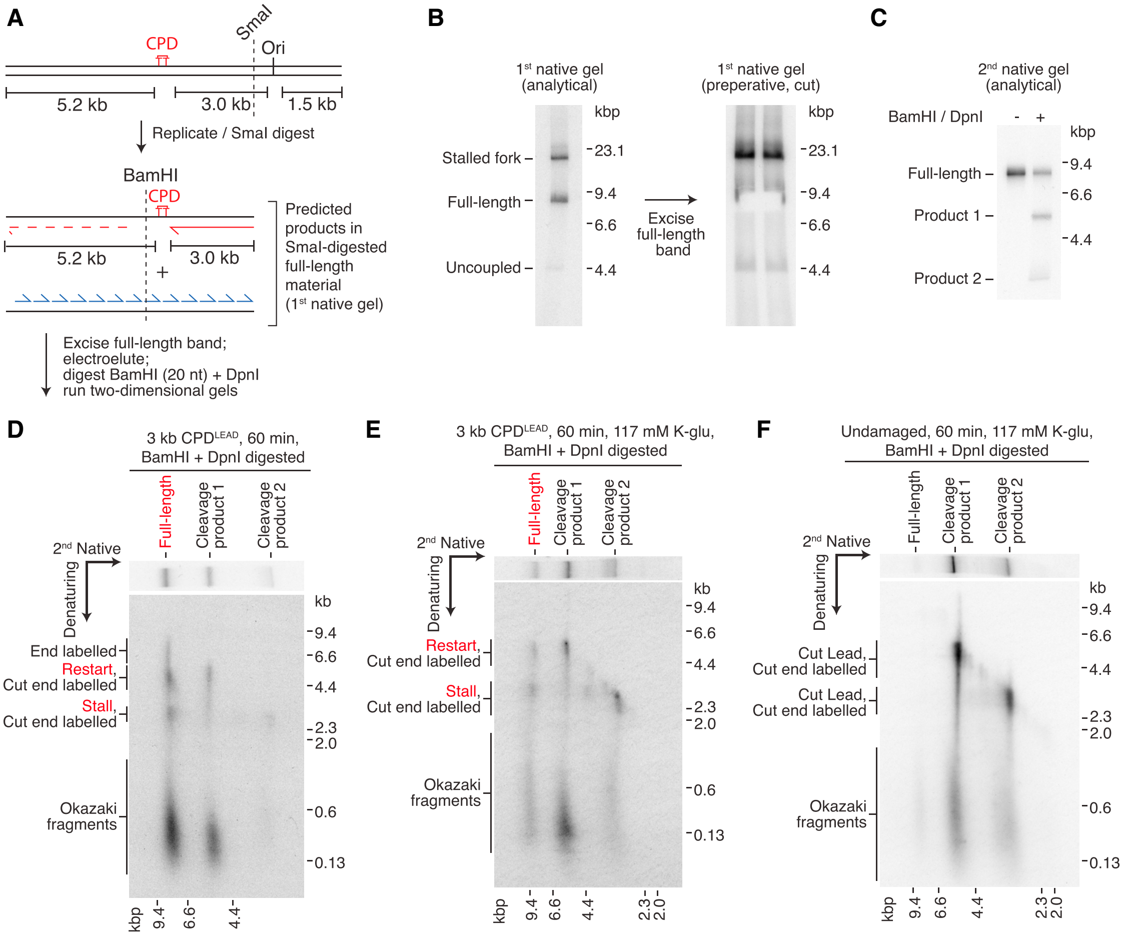

Figure 4. Evidence of Leading-Strand Re-priming by the Core Eukaryotic Replisome

(A) Schematic of the 3 kb CPD^{LEAD} template and the predicted replication products of leading-strand lesion bypass by re-priming followed by post-replicative SmaI digestion.

(B) SmaI-digested replication products (60 min) were run in a native gel (analytical, 2%, one lane; preparative, 98%, two lanes). Full-length products were electroeluted from excised preparative lane gel slices.

(C and D) Electroeluted DNA from (B) was digested with BamHI and DpnI and analyzed by native (C) and two-dimensional electrophoresis (BamHI + DpnI-digested sample) (D).

(E and F) Electroeluted full-length products from CPD^{LEAD} (E) and undamaged (F) templates in Figure S4C were digested with BamHI and DpnI and separated through two-dimensional gels.

In (D) and (E), products arising from leading-strand re-priming are shown in red.

restart products were efficiently synthesized upon addition of a 14-base oligonucleotide complementary to the leading-strand template immediately beyond CPD^{LEAD}, but not with a scrambled oligonucleotide (Figure 5D, denaturing, compare lanes 2 and 4). This was accompanied by an increase in full-length products and concomitant reduction in stalled forks (Figure 5D, native; compare lanes 2 and 4), indicating that leading-strand restart might accelerate replisome progression downstream of CPD^{LEAD}. Restart products were strictly dependent on the presence of CPD^{LEAD} (Figure S5E) and were synthesized equally efficiently in the presence or absence of Pol δ (Figure 5D, lanes 3 and 4), demonstrating that Pol δ was not required to elongate the re-priming oligonucleotide in these reactions. The extent of both stalled fork rescue and restart decreased as the oligonucleotide binding site was shifted further downstream of CPD^{LEAD} (Figures 5D and S5F), consistent with the detection of stalled forks located at between ~400 bp and ~1,000 bp beyond CPD^{LEAD} (Figures 5B and 5C). Hence, while CMG unwinding is not limiting for reinitiation close to CPD^{LEAD}, unwinding past the site of oligonucleotide binding is a prerequisite for restart. Time-course and pulse-chase experiments (Figures 5E–5G and S5G) confirmed that the re-priming oligonucleotide accelerated stalled fork resolution, revealing that re-establishing leading-strand synthesis led to faster replisome progression compared to uncoupled fork movement.

Leading- and Lagging-Strand Priming Are Mechanistically Distinct

Lagging-strand priming frequency is stimulated by increased Pol α concentrations (Yeeles et al., 2017) (Figure 2E) and

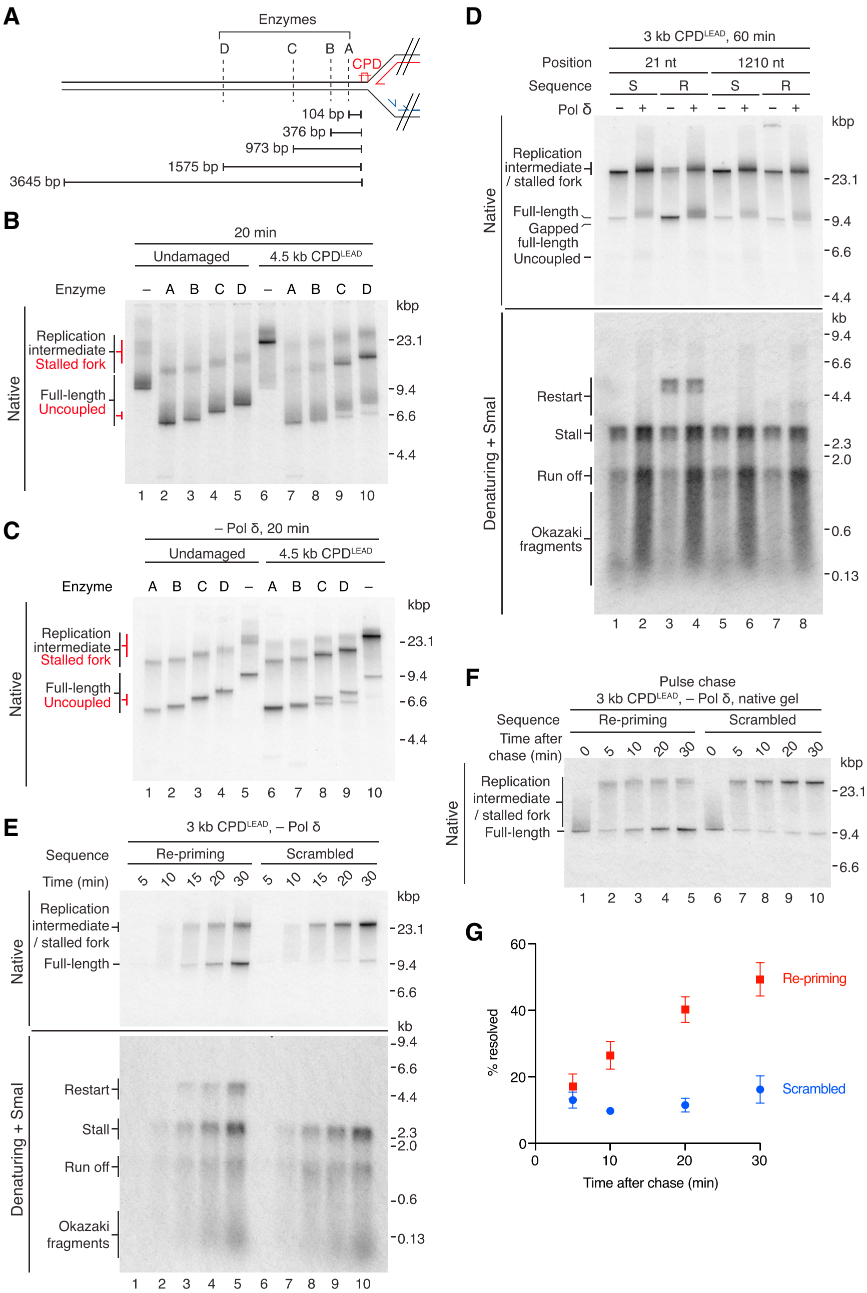

Figure 5. Priming Is the Main Limiting Step in Leading-Strand Restart

(A) Schematic of the downstream region of the AhdI-linearized 4.5 kb CPD^LEAD template and the positions of restriction sites used to truncate the template prior to replication. Distances are measured from the CPD to the nucleotide after which the first cut is made by the restriction enzyme.

(B and C) Replication reactions performed on the templates illustrated in (A) in the presence (B) and absence (C) of Pol δ. Products specific to the CPD^LEAD templates are annotated in red.

(D) Replication of the 3 kb CPD^LEAD template in the presence of re-priming (R) or scrambled (S) oligonucleotides. The distance from the CPD to the distal end of the re-priming oligonucleotide binding site is illustrated (position).

(E and F) Time course (E) and pulse-chase experiments (F) in the presence of a re-priming (21 nt position) or scrambled oligonucleotide. (F) The chase was added at 4 min 50 s.

(G) Quantitation of pulse-chase experiments as performed in (F). Error bars represent the SEM from three experiments.

template chromatinization (Kurat et al., 2017). To test whether leading-strand re-priming was modulated by these factors, we performed replication assays on CPD^LEAD templates with increased Pol α concentrations (Figure S6A) and following chromatinization (Figures S6B–S6D). However, no clear stimulation of leading-strand reinitiation or fork progression was observed.

Human Pol α priming on ssDNA templates is strongly inhibited by RPA (Collins and Kelly, 1991). Likewise, although yeast Pol α efficiently catalyzed primer synthesis and elongation on M13 ssDNA in the absence of RPA (Figure 6A, lane 1), nucleotide incorporation was greatly reduced by saturating levels of RPA (Figure 6A, lanes 4 and 5). DNA synthesis was resistant to saturating levels of RPA when a primer was annealed to the template (Figure 6A, compare lanes 5 and 10), demonstrating that Pol α priming, but not polymerase activity, was inhibited by RPA on the unprimed template. However, at subsaturating RPA levels, Pol α retained priming activity (Figure 6A, lanes 2 and 3). We therefore considered that leading-strand re-priming might be sensitive to RPA levels.

Because neither replication fork progression downstream of CPD^LEAD (Figures 3B, 3C, and S3A–S3D) nor oligonucleotide-dependent leading-strand restart (Figure 5D) was significantly altered by Pol δ, we investigated the influence of RPA on restart in its absence to facilitate detection of potentially rare events that might be obscured by Pol-δ-dependent template labeling and strand-displacement synthesis. Standard replication reactions were performed with 60 nM RPA. Replication at 20 and 200 nM RPA revealed less uncoupled products were synthesized at the lower RPA concentration (Figures S7A–S7D), which could indicate increased restart. However, prominent restart products were not detected in the denaturing gel, even after 120 min (Figure S7B, lane 5). We therefore analyzed replication at long time points after SmaI digestion in two-dimensional gels to better resolve nascent strands specifically associated with full-length products. Replication past CPD^LEAD in a reaction containing 10 nM RPA and 100 nM Pol α yielded full-length products (native) composed of stalled leading strands, Okazaki

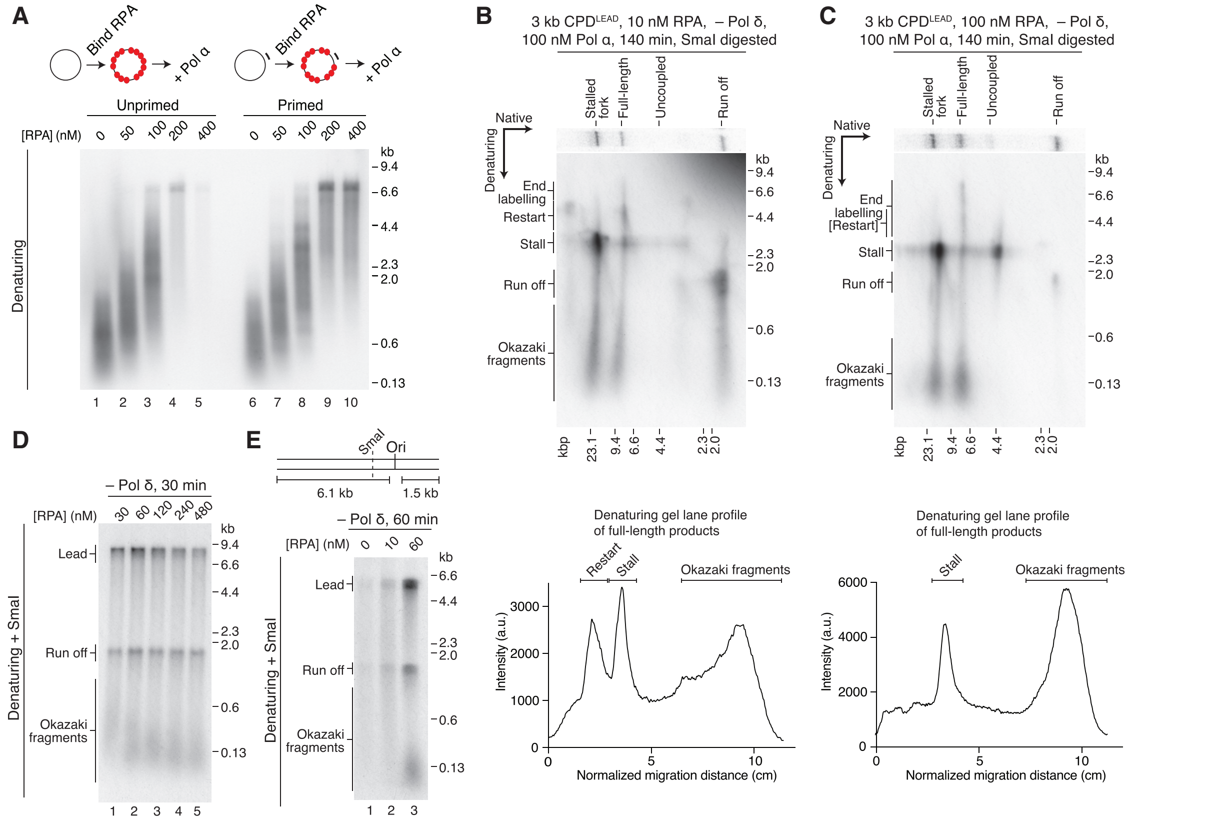

Figure 6. RPA Levels Differentially Affect Lagging- and Leading-Strand Priming

(A) Primase assay. RPA was pre-bound to unprimed (left) and primed (right) M13mp18 ssDNA for 10 min before addition of Pol α for 20 min. 120 nM RPA is saturating assuming a binding footprint of 30 nt.

(B and C) Two-dimensional gels of replication assays performed on the 3 kb CPD^{LEAD} template with 10 nM (B) or 100 nM (C) RPA. Lane profiles showing the constituents (denaturing) of the full-length products are shown below each gel.

(D) RPA titration on an AhdI-linearized undamaged template.

(E) RPA titration on a truncated undamaged template as illustrated.

fragments, and a distinct population migrating at the expected position for leading-strand restart (Figure 6B). Because restart products were directly detectable in a two-dimensional gel without prior enrichment, the data suggested that leading-strand re-priming was occurring more efficiently under these conditions. In support of this conclusion, at 100 nM RPA, there was little signal above background in the region of the gel where putative restart products should migrate (Figures 6B and 6C; compare lane profiles of full-length products). Similar results were obtained with 20 nM Pol α in the replication buffer (Figures S7E–S7G). Thus, although still relatively inefficient, leading-strand restart appeared to be influenced by RPA availability over a range of Pol α concentrations.

Uncoupled products were once again diminished at lower RPA concentrations (Figures 6B, 6C, and S7E–S7G). However, these reductions cannot solely result from increased restart, because they were not accompanied by sufficient increases in the proportion of stalled leading-strands comprising the full-length population. Consistent with this interpretation, a greater proportion of stalled leading strands were present in the stalled fork population when RPA was reduced, demonstrating that although restart was more prominent, fewer replisomes reached the end of the template. Hence, a combination of restricted uncoupled fork progression and increased leading-strand restart likely contributes to fewer uncoupled products being synthesized when RPA is less abundant.

In contrast to the more prominent restart products observed at lower RPA concentrations, Okazaki fragments were routinely longer on both damaged and undamaged templates (Figures 6B–6D and S7A–S7D) and almost undetectable when RPA was omitted (Figure 6E), indicating that lagging-strand priming is likely stimulated by RPA. Taken together, these results reveal that leading- and lagging-strand priming exhibit several unanticipated mechanistic differences, notably their sensitivity to modulation by nucleosomes, RPA levels, and free Pol α concentration.

Increased Leading-Strand Restart following RPA Depletion

Under conditions of replication stress, in which forks globally stall in a short window of time, uncoupled replisome progression can cause significant ssDNA exposure and exhaustion of free

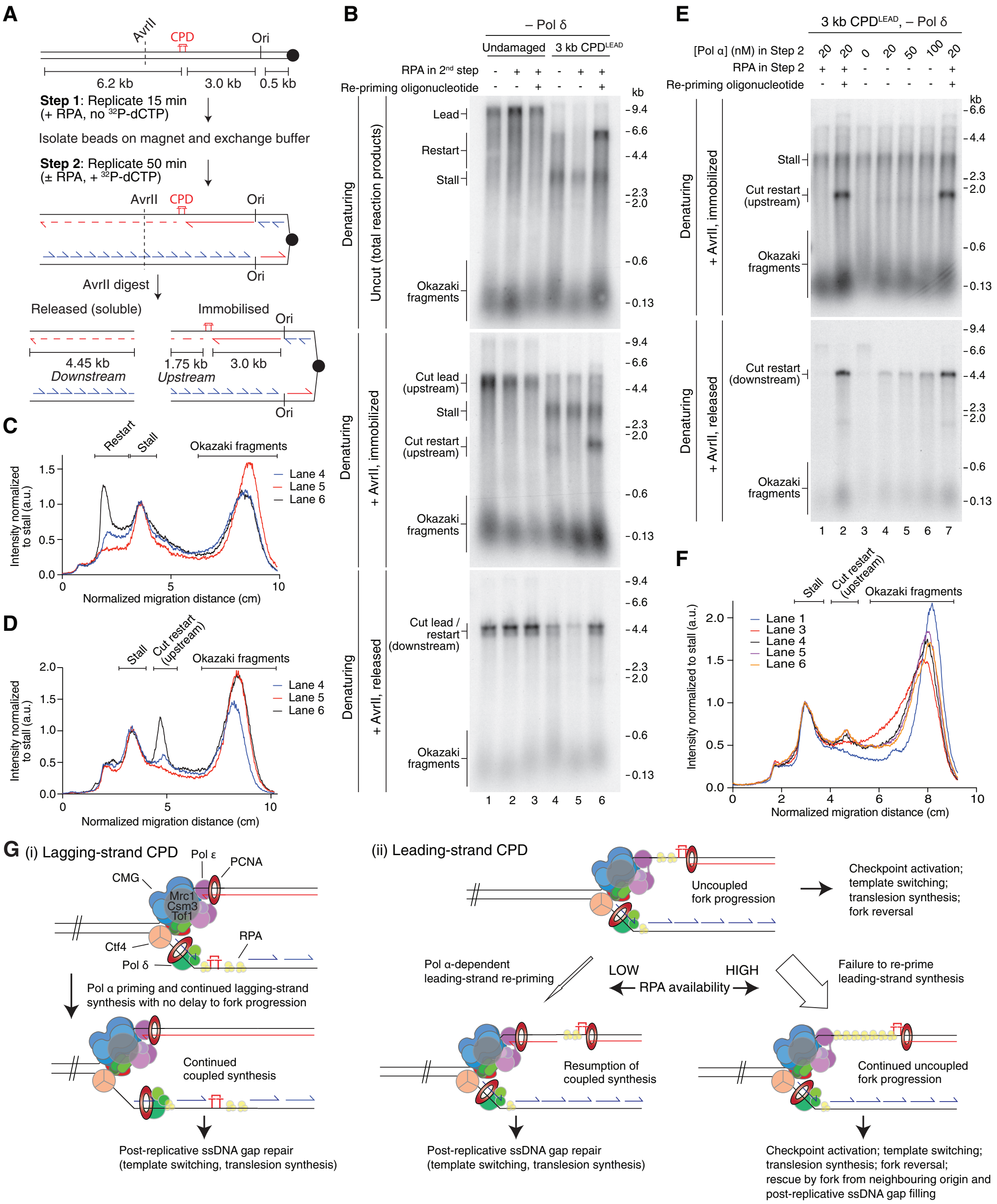

Figure 7. RPA Depletion Stimulates Pol α-Dependent Leading-Strand Restart

(A) Reaction scheme for bead-bound replication assays.

(B–D) Reactions performed as illustrated in (A) on undamaged and 3 kb CPD^{LEAD} templates (20 nM Pol α throughout). Products were separated through denaturing gels (B), and lane profiles for uncut (C) and immobilized AvrII-treated samples (D) are shown.

(E and F) Reaction performed as illustrated in (A) but with 5 nM Pol α in step 1 and varying concentrations of Pol α in step 2 as indicated. AvrII digested products were separated though denaturing gels (E), and lane profiles for the immobilized samples (F) are shown.

(G) Model of the initial response of the replisome to (i) lagging- and (ii) leading-strand CPDs.

RPA pools in mammalian cells (Toledo et al., 2013), and several lines of evidence suggest that RPA depletion also occurs in yeast (Toledo et al., 2017). To deplete RPA during replisome progression *in vitro*, we initiated replication on immobilized templates in the presence of RPA (Figure 7A, step 1), before exchanging the reaction buffer for one in which RPA was omitted (step 2). Note

that this approach will significantly reduce free RPA pools rather than eliminate them, as a small amount of reaction buffer is retained on the beads after step 1. For the first time, damage-dependent leading-strand reinitiation products were directly detectable in denaturing gels, but not when RPA was maintained in the reaction buffer (Figure 7B, compare lanes 1, 4, and 5, and Figure 7C). Moreover, restart products but not stalled leading strands were sensitive to a restriction enzyme (AvrII) mapping ~1.75 kb downstream of CPD^{LEAD} (Figure 7A); treatment with AvrII at the end of the reaction released a 4.45-kb downstream product (Figure 7B, bottom panel, lane 4), while the 1.75-kb upstream product remained immobilized (Figure 7B, middle panel, lane 4, and Figure 7D). Re-priming following RPA depletion occurred relatively close to CPD^{LEAD}, since the immobilized AvrII cleavage product was only slightly smaller than an oligonucleotide-dependent restart product (Figure 7B, middle panel; compare lanes 4 and 6; and Figure 7D, cut restart), which served as a positive control and marker of restart products in these experiments.

Finally, we exploited the bead-based assay to address whether leading-strand restart was dependent on Pol α, which was not possible in standard assays, since initiation is dependent on Pol α. Cut upstream restart products (immobilized) were only visible after AvrII digestion in reactions lacking RPA but containing Pol α in step 2 (Figure 7E, + AvrII, immobilized, and Figure 7F, cut restart). The same samples displayed a concomitant increase in cut downstream restart (Figure 7E, +AvrII, released). These results further support the conclusion that RPA availability influences leading-strand restart efficiency and confirm that Pol α is required for re-priming.

DISCUSSION

Our results describe the initial response of a eukaryotic replisome, assembled with purified proteins, to a CPD (Figure 7G), which displays the following key features: (1) a lagging-strand CPD is rapidly and efficiently bypassed, whereas a single leading-strand CPD significantly inhibits replisome progression; (2) template unwinding advances beyond the site of leading-strand damage; (3) the core eukaryotic replisome alone can re-establish leading-strand synthesis beyond a CPD by re-priming; (4) restart is inefficient because re-priming of the leading-strand template by Pol α is disfavored; and (5) the efficiency of leading-strand restart is modulated by RPA availability.

Bypass of a Lagging-Strand CPD

Efficient bypass of a lagging-strand CPD is consistent with replication past lagging-strand roadblocks in *Xenopus* extracts (Fu et al., 2011). The lack of a measurable delay during bypass supports the view that lagging-strand synthesis has little or no influence on CMG helicase progression. Inhibition of a single Okazaki fragment is in good agreement with daughter-strand gap size in yeast following UV exposure (Lopes et al., 2006). Such gaps are likely to be filled post-replicatively by DDT pathways.

Bypass of a Leading-Strand CPD by Re-priming

Following stalling of Pol ε at a CPD, template unwinding continues to advance downstream of the lesion, with lagging-strand synthesis continuing in at least a subset of replication forks. Further work is required to uncover the dynamics of template unwinding after polymerase stalling and the frequency with which lagging-strand synthesis continues. Based on the predicted footprint of CMG (20–40 nt) (Fu et al., 2011), ~60 bp need to be unwound to expose sufficient ssDNA for re-priming, which our data suggest happens at the majority of forks. If re-priming occurs, then we propose that normal fork progression is re-established only once Pol ε associated with CMG resumes leading-strand synthesis. Our data cannot distinguish whether the primer synthesized by Pol α is directly transferred to a CMG-Pol ε complex or whether a "free" polymerase is required to "catch up" to advancing CMG-Pol ε. If a free polymerase is required, then Pol ε can likely fulfill this role *in vitro*, as Pol δ was dispensable for restart, both with re-priming oligonucleotides and at reduced RPA levels. We speculate that Pol δ would normally perform this task when both polymerases are present, in a similar manner to its proposed role in establishing leading-strand synthesis (Yeeles et al., 2017). It is also unknown whether the stalled Pol ε remains associated with the translocating CMG and/or the 3′ end of the stalled leading strand. These will be interesting questions to address in the future.

Inefficient Re-priming Causes Prolonged Replication Fork Stalling and Uncoupling

Our work provides direct evidence for a causative link between polymerase stalling, specifically at leading-strand template DNA damage and delayed replisome progression. This had been assumed for many years, since fork stalling *in vivo* occurs following treatment with DNA damaging agents, but it had not been possible to exclude other indirect effects of genotoxins or the involvement of DNA repair complexes and the transcription machinery. Because fork progression can be rapidly and efficiently rescued by artificially mimicking leading-strand "re-priming" with an oligonucleotide, we propose that the underlying basis for prolonged fork stalling and continued uncoupled synthesis may simply be a failure to re-prime the leading-strand template. If correct, then observations of stalled, slow-moving, and uncoupled replication forks *in vivo* (Lopes et al., 2006; Tercero and Diffley, 2001) provide strong evidence that leading-strand re-priming downstream of damage is generally inefficient. Importantly, we now also show that the core replisome alone has the capacity to sustain uncoupled fork progression for many kilobases downstream of damage.

Different Mechanisms of Leading- and Lagging-Strand Priming

The rarity of leading-strand priming by Pol α downstream of damage may reflect a general inefficiency of leading-strand priming. This would have significant implications for the initiation of leading-strand synthesis at origins, supporting a model whereby leading strands initiate from extension of the first Okazaki fragments primed by forks moving in the opposite direction. This could explain the involvement of Pol δ early in leading-strand synthesis (Daigaku et al., 2015; Garbacz et al., 2018; Yeeles et al., 2017).

A need to prevent promiscuous leading-strand priming when ssDNA is exposed on the leading-strand template during

unperturbed fork progression may necessitate differential priming efficiency by the replisome. Indeed, a recent study found that ssDNA is frequently exposed on the leading strand by the *E. coli* replisome (Graham et al., 2017). It has also been suggested that periodic dissociation of the flexible Pol ε catalytic domain (Zhou et al., 2017) from the 3′ end of the leading strand enables PCNA loading for mismatch repair and nucleosome assembly (Georgescu et al., 2017).

A functional interaction between Pol α and CMG is required for repeated lagging-strand priming (Georgescu et al., 2015). If Pol α is recruited to CMG for lagging-strand priming via a direct protein-protein interaction, then the interface may not be compatible with promoting efficient leading-strand priming, because the unwound template strands are likely to be some distance apart (Georgescu et al., 2017). Pol α physically associates with replisome progression complexes via an interaction with Ctf4, which in turn binds GINS (Gambus et al., 2009). However, Ctf4 is dispensable for priming in reconstituted systems (Georgescu et al., 2015; Yeeles et al., 2015). Because Ctf4 was present in all our experiments we cannot exclude a role in leading-stand re-priming, although its location at the front of the replisome (Sun et al., 2015) would seem unfavorable. It will be interesting to discover how Pol α is recruited to replication forks for lagging- and leading-strand priming.

Implications for Pathway Choice in Rescuing Stalled Replication Forks

In striking contrast to *E. coli* (Yeeles and Marians, 2013; Yeeles and Marians, 2011), our results indicate that leading-strand re-priming by the replisome alone may not be a major initial response to DNA damage in budding yeast. These differences may reflect increased selective pressure to evolve a robust re-priming mechanism in bacteria, since the average replicon size is two orders of magnitude longer than in yeast. Indeed, given the relatively short inter-origin distance in *Saccharomyces cerevisiae* (~50 kb), isolated stalled forks should be rapidly rescued by forks from neighboring origins. We propose that this may be the principal mechanism by which stalled forks are rescued when DNA damage is infrequently encountered, which would generate daughter-strand gaps for processing by DDT pathways.

Our data do not exclude the possibility that additional factors and/or post-translational modifications (e.g., RPA phosphorylation or SUMOylation) (Maréchal and Zou, 2015) promote leading-strand re-priming *in vivo*. It is notable that some eukaryotes encode an additional primase, PrimPol (Bianchi et al., 2013; García-Gómez et al., 2013; Wan et al., 2013). PrimPol may have evolved to compensate for the inefficiency of Pol α in catalyzing leading-strand re-priming, consistent with the increased uncoupling observed downstream of a G-quadruplex in PrimPol-deficient cells, despite the presence of Pol α (Schiavone et al., 2016).

Our discovery that leading-strand re-priming is modulated by RPA availability *in vitro* raises the possibility that re-priming might fulfill a more significant role under conditions of catastrophic global fork arrest, which can have a dramatic effect on free RPA levels *in vivo* (Toledo et al., 2013, 2017). We note that ssDNA gaps behind replication forks in both daughter strands (Lopes et al., 2006) were detected following treatment of yeast cells with UV doses predicted to rapidly interfere with nascent-strand synthesis at most active replication forks. In addition, a number of other ssDNA-binding proteins, including RAD51 and RADX, have recently been implicated in controlling the dynamics of stalled fork reversal and restart in mammalian cells (Dungrawala et al., 2017; Zellweger et al., 2015). Complex dynamic interactions between ssDNA, RPA, and other ssDNA-binding proteins may be important in controlling the exact mechanisms of rescuing stalled forks in higher eukaryotes.

Stalled replication forks can also be restarted by DDT pathways. Translesion synthesis polymerases may target stalled leading strands to facilitate synthesis past the damage and the subsequent resumption of coupled fork progression. At least a subset of stalled forks in our experiments exhibited continued lagging-strand synthesis and would therefore be poised for rescue by template switching. A choice between these pathways may depend on the nature of the DNA damage. Such structures are also predicted to activate the DNA replication checkpoint, because they will present RPA-coated ssDNA (Zou and Elledge, 2003) and continued primer synthesis by Pol α is required for maximal checkpoint activation in *Xenopus* (Van et al., 2010). Moreover, our data imply that it is lagging-strand priming, rather than re-priming on the leading strand, that generates the free 5′ ends that stimulate checkpoint activation (MacDougall et al., 2007) under normal conditions.

The reconstitution of unidirectional collisions between a eukaryotic replisome and site-specific CPDs has provided insights into the earliest events in the process of DNA damage bypass. The system described herein also provides a platform for future reconstitution and characterization of pathways that respond to stalled forks, notably translesion synthesis, template switching, replication fork reversal, and the DNA replication checkpoint.

STAR★METHODS

Detailed methods are provided in the online version of this paper and include the following:

- KEY RESOURCES TABLE
- CONTACT FOR REAGENT AND RESOURCE SHARING
- EXPERIMENTAL MODEL AND SUBJECT DETAILS
- METHOD DETAILS
  - Protein purification
  - Replication templates
  - Standard replication assays
  - Chromatin experiments
  - Replication assays on immobilized templates
  - Primase assay
  - Gel imaging and presentation
- QUANTIFICATION AND STATISTICAL ANALYSIS

SUPPLEMENTAL INFORMATION

Supplemental Information includes seven figures and one table and can be found with this article online at https://doi.org/10.1016/j.molcel.2018.04.022.

ACKNOWLEDGMENTS

We thank J. Diffley for reagents, G. Coster for advice on ORC-binding sequences, and K. Marians, J. Sale, and members of the Yeeles laboratory for

critical reading of the manuscript. The Yeeles laboratory is funded by the Medical Research Council (MC_UP_1201/12). M.R.G.T. is supported by a Sir Henry Wellcome Postdoctoral Fellowship from the Wellcome Trust (110014/Z/15/Z) and St. John's College, Cambridge.

AUTHOR CONTRIBUTIONS

M.R.G.T. and J.T.P.Y. conceived the study, performed experiments, and wrote the manuscript.

DECLARATION OF INTERESTS

M.R.G.T.'s fellowship is administered by University of Dundee. J.T.P.Y. declares no competing interests.

Received: November 27, 2017
Revised: March 9, 2018
Accepted: April 26, 2018
Published: May 31, 2018

REFERENCES

Bianchi, J., Rudd, S.G., Jozwiakowski, S.K., Bailey, L.J., Soura, V., Taylor, E., Stevanovic, I., Green, A.J., Stracker, T.H., Lindsay, H.D., and Doherty, A.J. (2013). PrimPol bypasses UV photoproducts during eukaryotic chromosomal DNA replication. Mol. Cell *52*, 566–573.

Biswas, D., Yu, Y., Prall, M., Formosa, T., and Stillman, D.J. (2005). The yeast FACT complex has a role in transcriptional initiation. Mol. Cell. Biol. *25*, 5812–5822.

Collins, K.L., and Kelly, T.J. (1991). Effects of T antigen and replication protein A on the initiation of DNA synthesis by DNA polymerase alpha-primase. Mol. Cell. Biol. *11*, 2108–2115.

Coster, G., Frigola, J., Beuron, F., Morris, E.P., and Diffley, J.F. (2014). Origin licensing requires ATP binding and hydrolysis by the MCM replicative helicase. Mol. Cell *55*, 666–677.

Daigaku, Y., Davies, A.A., and Ulrich, H.D. (2010). Ubiquitin-dependent DNA damage bypass is separable from genome replication. Nature *465*, 951–955.

Daigaku, Y., Keszthelyi, A., Müller, C.A., Miyabe, I., Brooks, T., Retkute, R., Hubank, M., Nieduszyski, C.A., and Carr, A.M. (2015). A global profile of replicative polymerase usage. Nat. Struct. Mol. Biol. *22*, 192–198.

Devbhandari, S., Jiang, J., Kumar, C., Whitehouse, I., and Remus, D. (2017). Chromatin constrains the initiation and elongation of DNA replication. Mol. Cell *65*, 131–141.

Dungrawala, H., Bhat, K.P., Le Meur, R., Chazin, W.J., Ding, X., Sharan, S.K., Wessel, S.R., Sathe, A.A., Zhao, R., and Cortez, D. (2017). RADX promotes genome stability and modulates chemosensitivity by regulating RAD51 at replication forks. Mol. Cell *67*, 374–386.e5.

Frigola, J., Remus, D., Mehanna, A., and Diffley, J.F. (2013). ATPase-dependent quality control of DNA replication origin licensing. Nature *495*, 339–343.

Fu, Y.V., Yardimci, H., Long, D.T., Ho, T.V., Guainazzi, A., Bermudez, V.P., Hurwitz, J., van Oijen, A., Schärer, O.D., and Walter, J.C. (2011). Selective bypass of a lagging strand roadblock by the eukaryotic replicative DNA helicase. Cell *146*, 931–941.

Fumasoni, M., Zwicky, K., Vanoli, F., Lopes, M., and Branzei, D. (2015). Error-free DNA damage tolerance and sister chromatid proximity during DNA replication rely on the Polα/Primase/Ctf4 Complex. Mol. Cell *57*, 812–823.

Gambus, A., van Deursen, F., Polychronopoulos, D., Foltman, M., Jones, R.C., Edmondson, R.D., Calzada, A., and Labib, K. (2009). A key role for Ctf4 in coupling the MCM2-7 helicase to DNA polymerase alpha within the eukaryotic replisome. EMBO J. *28*, 2992–3004.

Garbacz, M.A., Lujan, S.A., Burkholder, A.B., Cox, P.B., Wu, Q., Zhou, Z.X., Haber, J.E., and Kunkel, T.A. (2018). Evidence that DNA polymerase δ contributes to initiating leading strand DNA replication in Saccharomyces cerevisiae. Nat. Commun. *9*, 858.

García-Gómez, S., Reyes, A., Martínez-Jiménez, M.I., Chocrón, E.S., Mourón, S., Terrados, G., Powell, C., Salido, E., Méndez, J., Holt, I.J., and Blanco, L. (2013). PrimPol, an archaic primase/polymerase operating in human cells. Mol. Cell *52*, 541–553.

Georgescu, R.E., Schauer, G.D., Yao, N.Y., Langston, L.D., Yurieva, O., Zhang, D., Finkelstein, J., and O'Donnell, M.E. (2015). Reconstitution of a eukaryotic replisome reveals suppression mechanisms that define leading/lagging strand operation. eLife *4*, e04988.

Georgescu, R., Yuan, Z., Bai, L., de Luna Almeida Santos, R., Sun, J., Zhang, D., Yurieva, O., Li, H., and O'Donnell, M.E. (2017). Structure of eukaryotic CMG helicase at a replication fork and implications to replisome architecture and origin initiation. Proc. Natl. Acad. Sci. USA *114*, E697–E706.

Graham, J.E., Marians, K.J., and Kowalczykowski, S.C. (2017). Independent and stochastic action of DNA polymerases in the replisome. Cell *169*, 1201–1213.e17.

Iyer, V.N., and Rupp, W.D. (1971). Usefulness of benzoylated naphthoylated DEAE-cellulose to distinguish and fractionate double-stranded DNA bearing different extents of single-stranded regions. Biochim. Biophys. Acta *228*, 117–126.

Karras, G.I., and Jentsch, S. (2010). The RAD6 DNA damage tolerance pathway operates uncoupled from the replication fork and is functional beyond S phase. Cell *141*, 255–267.

Kingston, I.J., Yung, J.S., and Singleton, M.R. (2011). Biophysical characterization of the centromere-specific nucleosome from budding yeast. J. Biol. Chem. *286*, 4021–4026.

Kurat, C.F., Yeeles, J.T.P., Patel, H., Early, A., and Diffley, J.F.X. (2017). Chromatin controls DNA replication origin selection, lagging-strand synthesis, and replication fork rates. Mol. Cell *65*, 117–130.

Lehmann, A.R. (1972). Postreplication repair of DNA in ultraviolet-irradiated mammalian cells. J. Mol. Biol. *66*, 319–337.

Lehmann, A.R., and Fuchs, R.P. (2006). Gaps and forks in DNA replication: rediscovering old models. DNA Repair (Amst.) *5*, 1495–1498.

Lopes, M., Foiani, M., and Sogo, J.M. (2006). Multiple mechanisms control chromosome integrity after replication fork uncoupling and restart at irreparable UV lesions. Mol. Cell *21*, 15–27.

MacDougall, C.A., Byun, T.S., Van, C., Yee, M.C., and Cimprich, K.A. (2007). The structural determinants of checkpoint activation. Genes Dev. *21*, 898–903.

Maréchal, A., and Zou, L. (2015). RPA-coated single-stranded DNA as a platform for post-translational modifications in the DNA damage response. Cell Res. *25*, 9–23.

McInerney, P., and O'Donnell, M. (2004). Functional uncoupling of twin polymerases: mechanism of polymerase dissociation from a lagging-strand block. J. Biol. Chem. *279*, 21543–21551.

Neelsen, K.J., and Lopes, M. (2015). Replication fork reversal in eukaryotes: from dead end to dynamic response. Nat. Rev. Mol. Cell Biol. *16*, 207–220.

On, K.F., Beuron, F., Frith, D., Snijders, A.P., Morris, E.P., and Diffley, J.F. (2014). Prereplicative complexes assembled in vitro support origin-dependent and independent DNA replication. EMBO J. *33*, 605–620.

Ruone, S., Rhoades, A.R., and Formosa, T. (2003). Multiple Nhp6 molecules are required to recruit Spt16-Pob3 to form yFACT complexes and to reorganize nucleosomes. J. Biol. Chem. *278*, 45288–45295.

Rupp, W.D., and Howard-Flanders, P. (1968). Discontinuities in the DNA synthesized in an excision-defective strain of Escherichia coli following ultraviolet irradiation. J. Mol. Biol. *31*, 291–304.

Schiavone, D., Jozwiakowski, S.K., Romanello, M., Guilbaud, G., Guilliam, T.A., Bailey, L.J., Sale, J.E., and Doherty, A.J. (2016). PrimPol is required for replicative tolerance of G quadruplexes in vertebrate cells. Mol. Cell *61*, 161–169.

Sun, J., Shi, Y., Georgescu, R.E., Yuan, Z., Chait, B.T., Li, H., and O'Donnell, M.E. (2015). The architecture of a eukaryotic replisome. Nat. Struct. Mol. Biol. *22*, 976–982.

Tercero, J.A., and Diffley, J.F. (2001). Regulation of DNA replication fork progression through damaged DNA by the Mec1/Rad53 checkpoint. Nature *412*, 553–557.

Toledo, L.I., Altmeyer, M., Rask, M.B., Lukas, C., Larsen, D.H., Povlsen, L.K., Bekker-Jensen, S., Mailand, N., Bartek, J., and Lukas, J. (2013). ATR prohibits replication catastrophe by preventing global exhaustion of RPA. Cell *155*, 1088–1103.

Toledo, L., Neelsen, K.J., and Lukas, J. (2017). Replication catastrophe: when a checkpoint fails because of exhaustion. Mol. Cell *66*, 735–749.

Van, C., Yan, S., Michael, W.M., Waga, S., and Cimprich, K.A. (2010). Continued primer synthesis at stalled replication forks contributes to checkpoint activation. J. Cell Biol. *189*, 233–246.

Wan, L., Lou, J., Xia, Y., Su, B., Liu, T., Cui, J., Sun, Y., Lou, H., and Huang, J. (2013). hPrimpol1/CCDC111 is a human DNA primase-polymerase required for the maintenance of genome integrity. EMBO Rep. *14*, 1104–1112.

Yeeles, J.T.P., and Marians, K.J. (2011). The Escherichia coli replisome is inherently DNA damage tolerant. Science *334*, 235–238.

Yeeles, J.T., and Marians, K.J. (2013). Dynamics of leading-strand lesion skipping by the replisome. Mol. Cell *52*, 855–865.

Yeeles, J.T., Poli, J., Marians, K.J., and Pasero, P. (2013). Rescuing stalled or damaged replication forks. Cold Spring Harb. Perspect. Biol. *5*, a012815.

Yeeles, J.T., Deegan, T.D., Janska, A., Early, A., and Diffley, J.F. (2015). Regulated eukaryotic DNA replication origin firing with purified proteins. Nature *519*, 431–435.

Yeeles, J.T.P., Janska, A., Early, A., and Diffley, J.F.X. (2017). How the eukaryotic replisome achieves rapid and efficient DNA replication. Mol. Cell *65*, 105–116.

Zellweger, R., Dalcher, D., Mutreja, K., Berti, M., Schmid, J.A., Herrador, R., Vindigni, A., and Lopes, M. (2015). Rad51-mediated replication fork reversal is a global response to genotoxic treatments in human cells. J. Cell Biol. *208*, 563–579.

Zeman, M.K., and Cimprich, K.A. (2014). Causes and consequences of replication stress. Nat. Cell Biol. *16*, 2–9.

Zhou, J.C., Janska, A., Goswami, P., Renault, L., Abid Ali, F., Kotecha, A., Diffley, J.F.X., and Costa, A. (2017). CMG-Pol epsilon dynamics suggests a mechanism for the establishment of leading-strand synthesis in the eukaryotic replisome. Proc. Natl. Acad. Sci. USA *114*, 4141–4146.

Zou, L., and Elledge, S.J. (2003). Sensing DNA damage through ATRIP recognition of RPA-ssDNA complexes. Science *300*, 1542–1548.

STAR★METHODS

KEY RESOURCES TABLE

REAGENT or RESOURCE	SOURCE	IDENTIFIER
Bacterial and Virus Strains		
Escherichia coli: Rosetta^{TM} 2(DE3) strain: F^{-} *ompT hsdS*$_{B}$(r_B^- m_B^-) *gal dcm* (DE3) pRARE2 (Cam^{R})	Novagen / Merck Millipore	Cat# 71400
Chemicals, Peptides, and Recombinant Proteins		
3X FLAG peptide	Sigma	Cat# F4799
Anti-FLAG M2 affinity gel	Sigma	Cat# A2220
Calmodulin-Sepharose 4B	GE Healthcare / Sigma	Cat# 17-0529-01
Calmodulin Affinity Resin	Agilent	Cat# 214303
Bio-Gel HT Hydroxyapatite	Bio-Rad	Cat# 1300150
Cdt1-Mcm2-7	Coster et al., 2014	N/A
ORC	Frigola et al., 2013	N/A
Cdc6	Frigola et al., 2013	N/A
DDK	On et al., 2014	N/A
Sld3/7	Yeeles et al., 2015	N/A
Cdc45	Yeeles et al., 2015	N/A
Dpb11	Yeeles et al., 2015	N/A
Sld2	Yeeles et al., 2015	N/A
GINS	Yeeles et al., 2015	N/A
Pol ε	Yeeles et al., 2015	N/A
S-CDK	Yeeles et al., 2015	N/A
Mcm10	Yeeles et al., 2015	N/A
Pol α	Yeeles et al., 2015	N/A
RPA	Devbhandari et al., 2017	N/A
Ctf4	Yeeles et al., 2015	N/A
Topo I	Yeeles et al., 2017	N/A
Mrc1	Yeeles et al., 2017	N/A
Csm3/Tof1	Yeeles et al., 2017	N/A
RFC	Yeeles et al., 2017	N/A
PCNA	Yeeles et al., 2017	N/A
Pol δ	Yeeles et al., 2017	N/A
Histones	Kurat et al., 2017	N/A
Nap1	Kurat et al., 2017	N/A
Isw1a	Kurat et al., 2017	N/A
FACT	Kurat et al., 2017	N/A
Nhp6	Kurat et al., 2017	N/A
Experimental Models: Organisms/Strains		
yAM33 (Cdt1-Mcm2-7 purification)	Coster et al., 2014	N/A
ySD-ORC (ORC purification)	Frigola et al., 2013	N/A
ySDK8 (DDK purification)	On et al., 2014	N/A
yTD6 (Sld3/7 purification)	Yeeles et al., 2015	N/A
yTD8 (Sld2 purification)	Yeeles et al., 2015	N/A
yJY13 (Cdc45 purification)	Yeeles et al., 2015	N/A
yJY26 (Dpb11 purification)	Yeeles et al., 2015	N/A
yAJ2 (Pol ε purification)	Yeeles et al., 2015	N/A

(*Continued on next page*)

Continued		
REAGENT or RESOURCE	SOURCE	IDENTIFIER
yAE37 (S-CDK purification)	Yeeles et al., 2015	N/A
yAE40 (Ctf4 purification)	Yeeles et al., 2015	N/A
yJY23 (Pol α purification)	Yeeles et al., 2015	N/A
yAE34 (Pol δ purification)	Yeeles et al., 2017	N/A
yAE41 (RFC purification)	Yeeles et al., 2017	N/A
yAE42 (Topo I purification)	Yeeles et al., 2017	N/A
yAE48 (Csm3/Tof1 purification)	Yeeles et al., 2017	N/A
yJY32 (Mrc1 purification)	Yeeles et al., 2017	N/A
yCFK1 (Isw1a purification)	Kurat et al., 2017	N/A
Oligonucleotides		
See Table S1 for all DNA oligonucleotides used in this study		
Recombinant DNA		
pBluescript II KS(–) Phagemid	Agilent Technologies	Cat# 212208-51
ZN3: replication template	This paper	N/A
ZN5SP1: replication template	This paper	N/A
M13mp18 Single-stranded DNA: primase assay	New England Biolabs	Cat# N4040S
λ DNA-HindIII Digest: molecular weight markers	New England Biolabs	Cat# N3012S
pJM126 (RPA purification)	Addgene	#49339
pAM3 (Cdc6 purification)	Frigola et al., 2013	N/A
vJY19 (PCNA purification)	Yeeles et al., 2017	N/A
pRJ1228-Nhp6 (Nhp6 purification)	Ruone et al., 2003	N/A
pCDFduet.H2A-H2B (Histones purification)	Kingston et al., 2011	N/A
pETduet.H3-H4 (Histones purification)	Kingston et al., 2011	N/A
pJFDJ5 (GINS purification)	Yeeles et al., 2015	N/A
pET28a-Mcm10 (Mcm10 purification)	Yeeles et al., 2015	N/A
pTF175 (FACT purification)	Biswas et al., 2005	N/A
pJW22 (FACT purification)	Biswas et al., 2005	N/A
pCFK1 (Nap1 purification)	Kurat et al., 2017	N/A
Software and Algorithms		
ImageJ	National Institute of Health	https://imagej.nih.gov/ij/

CONTACT FOR REAGENT AND RESOURCE SHARING

Further information and requests for resources and reagents should be directed to and will be fulfilled by the Lead Contact, Joseph Yeeles (jyeeles@mrc-lmb.cam.ac.uk).

EXPERIMENTAL MODEL AND SUBJECT DETAILS

Proteins were purified from *Saccharomyces cerevisiae* strains (genotype: *MAT**a** ade2-1 ura3-1 his3-11,15 trp1-1 leu2-3,112 can1-100 bar1::Hyg pep4::KanMX*) modified to overexpress proteins of interest as detailed in the Key Resources table by transforming with linearized plasmids using standard genetic procedures; or *Escherichia coli* Rosetta^{TM} 2(DE3) cells (Novagen) (genotype: F^{-} *ompT hsdS*$_B$(r_B^{-} m_B^{-}) *gal dcm* (DE3) pRARE2 (Cam^R)) transformed with plasmids for overexpression of proteins of interest as detailed in the Key Resources Table.

METHOD DETAILS

Protein purification

Proteins were purified as described previously (Yeeles et al., 2015, 2017; Kurat et al., 2017; Devbhandari et al., 2017). All yeast protein expression strains, and plasmids for protein purification from *Escherichia coli* are listed in the Key Resources Table. A brief description of the purification strategy for each protein is listed below.

ORC – ORC, carrying a CBP-TEV tag on the Orc1 subunit, was purified using affinity purification with Calmodulin Sepharose 4B (GE Healthcare), followed by gel filtration through a Superdex 200 column.
Cdc6 – GST-tagged Cdc6 was bound to Glutathione Sepharose 4B (GE Healthcare) and released by cleavage with GST-tagged 3C protease. The eluted protein was further purified using a Bio-Gel HT hydroxyapatite column (Bio-Rad).
Cdt1-Mcm2-7 – Cdt1-Mcm2-7 with a CBP-TEV tag on the N terminus of Mcm3 was purified using a Calmodulin Sepharose 4B column, followed by Superdex 200 (GE Healthcare) gel filtration.
DDK – DDK carrying a CBP tag on Dbf4 was bound to and eluted from Calmodulin Sepharose 4B resin. The eluted protein was dephosphorylated with lambda protein phosphatase and was then further purified by gel filtration (Superdex 200).
S-CDK – The S-CDK complex carrying a N-terminal CBP-TEV tag on Clb5 was bound to Calmodulin Affinity Resin (Agilent). Protein was eluted by cleavage with Tobacco Etch Virus protease and was further purified by gel filtration (Superdex 200).
Cdc45 – Cdc45 carrying a double internal FLAG tag was immunoprecipitated using Anti-FLAG M2 affinity gel (Sigma). The resulting eluate was further purified using a Bio-Gel HT hydroxyapatite column.
Sld3/7 – Sld3/7 carrying a C-terminal TCP tag on Sld3 was first bound to IgG Sepharose 6 Fast Flow resin (GE Healthcare) and the protein eluted by cleavage with Tobacco Etch Virus protease. The His-tagged protease was removed by passing the eluate over Ni-NTA resin (QIAGEN) and the flow through was further purified through a Superdex 200 gel filtration column.
Sld2 – 3xFLAG-tagged Sld2 (C-terminal tag) was precipitated from cell lysate with ammonium sulfate, resuspended in buffer, and then immunoprecipitated with Anti-FLAG M2 affinity gel. Protein was eluted with 3xFLAG peptide (Sigma) and the peptide was removed by binding and eluting the protein from a HiTrap SP HP column (GE Healthcare).
Dpb11 – Dpb11-3xFLAG was purified using Anti-FLAG M2 affinity gel followed by MonoS (GE Healthcare) chromatography.
Pol ε – Pol ε, tagged with a C-terminal CBP tag on Dpb4, was purified sequentially over Calmodulin Sepharose 4B, HiTrap Heparin HP (GE Healthcare) and Superdex 200 columns.
GINS – The GINS complex, modified with a 6xHis tag at the N terminus of Psf3, was bound to Ni-NTA and eluted with increasing imidazole concentration. Eluted protein was further purified over MonoQ (GE Healthcare) and Superdex 200 columns, followed a second Ni-NTA column.
Pol α – Pol alpha – primase, modified with an N-terminal CBP tag on Pri1 was purified sequentially over Calmodulin Sepharose 4B, MonoQ and Superdex 200 columns.
Mcm10 – 6xHis-Mcm10 was purified over Ni-NTA followed by two rounds of MonoS (GE Healthcare) chromatography.
Ctf4 – CBP-TEV-Ctf4 was purified by Calmodulin Sepharose 4B chromatography, followed by MonoQ and Superdex 200 gel filtration columns.
Mrc1 – Mrc1-2xFLAG was purified by FLAG immunoprecipitation followed by MonoQ chromatography.
Topo I – CBP-TEV-Topo I was bound to Calmodulin Sepharose 4B. Protein was eluted with Tobacco Etch Virus protease. His-tagged protease was removed by passing the eluate over a TALON column (Clontech) and the flow through was concentrated and separated through a Superdex 200 column.
Csm3/Tof1 – Csm3/Tof1 carrying a N-terminal CBP-TEV tag on Csm3 was bound to Calmodulin Sepharose 4B. Protein was eluted with Tobacco Etch Virus protease. His-tagged protease was removed by passing the eluate over a TALON column and the flow through was concentrated and separated through a Superdex 200 column.
RFC – The RFC complex containing an N-terminal CBP tag on Rfc3 was purified by sequential chromatography over Calmodulin Sepharose 4B, MonoS and Superdex 200 columns.
PCNA – Native PCNA was purified following overexpression in *Escherichia coli.* Nucleic acids were precipitated from the cell lysate with polymin P and then proteins were selectively precipitated with ammonium sulfate. Precipitated material was resuspended in buffer and then applied to HiTrap SP HP and HiTrap Heparin HP columns assembled in tandem. The flow through containing PCNA was further purified over HiTrap DEAE Fast Flow (GE Healthcare) and MonoQ columns.
Pol δ – Pol δ with a C-terminal TEV-CBP tag on Pol32 was purified over Calmodulin Sepharose 4B, HiTrap Heparin HP and Superdex 200 columns.
RPA – Native RPA was purified following overexpression in *Escherichia coli.* Protein was purified over HiTrap Blue HP (GE Healthcare), ssDNA cellulose (Sigma) and MonoQ columns.
Isw1a – Isw1a carrying a C-terminal 3xFLAG tag on Ioc3 was immunoprecipitated from cell lysate using Anti-FLAG M2 affinity gel. Protein was eluted with 3xFLAG peptide and was further purified by MonoQ chromatography.
Histones – Native histones were purified following overexpression in *Escherichia coli* by HiTrap Heparin HP and Superdex 200 chromatography.
Nap1 – GST-Nap1 was bound to Glutathione Sepharose 4B and eluted from the resin with GST-3C protease. Eluted protein was further purified on a MonoQ column.
FACT – His-tagged FACT was purified by TALON and MonoQ chromatography.
Nhp6 – Native Nhp6 was purified following overexpression in *Escherichia coli.* Proteins were precipitated from the cell lysate with trichloroacetic acid. The precipitated protein was resuspended in buffer and further purified over a HiTrap SP HP column.

Replication templates

Design and construction

The DNA replication template was produced by direct synthesis of a modified version of the genomic DNA surrounding the early-firing origin ARS306 and subsequent cloning and modification in pBluescript II KS(–). The complete sequence of the resulting two plasmids used for replication of damaged DNA introduced at two different sites is given below.

A region of genomic DNA on chromosome III spanning approximately 7.5 kb left and 0.2 kb right of ARS306 was chosen from the *Saccharomyces* Genome Database. Modifications were made to (1) introduce restriction enzyme sites to facilitate cloning of three different fragments of this region into pBluescript II KS(–) (KpnI, EagI, SalI, SacI); (2) introduce several unique diagnostic restriction enzyme sites in proximity to both sites of DNA damage cloning, used in a variety of different assays; (3) introduce two closely spaced restriction enzymes for cloning of cassettes containing BbvCI nicking endonuclease sites for the subsequent cloning of DNA damage at two different locations approximately 3 kb (PstI and BamHI) or 4.5 kb (SphI and SpeI) left of ARS306; (4) mutate any sequences outside of the origin with a strong ORC binding consensus, to facilitate tight origin specificity of replication initiation; (5) introduce other unique restriction enzyme sites for other purposes, including for asymmetric template linearization with respect the origin. The modified sequence was synthesized by Invitrogen GeneArt Gene Synthesis in three segments flanked by (1) KpnI and SalI; (2) SalI and EagI; (3) EagI and SacI. These segments were cloned using these restriction sites into the multiple cloning site of pBluescript II KS(–). The resulting plasmids were then digested with either PstI and BamHI, or SphI and SpeI, for introduction of cassettes for the subsequent cloning of DNA damage at two different positions.

The strategy for introducing site-specific DNA damage is based on nicking at two sites with the nicking endonuclease Nt.BbvCI to release a short oligonucleotide, which is competed away to generate gapped DNA, which can then be annealed and ligated to a synthetic oligonucleotide with or without a chemically synthesized cyclobutane pyramidine dimer (CPD). To achieve this, the following cassette for cloning DNA damage, comprising an AflII restriction enzyme site (underlined) flanked by two Nt.BbvCI sites (italicized; **^** indicates position of top strand nicking), was designed: *CC***^***TCAGC*A<u>CTTAAG</u>T*CC***^***TCAGC*. This sequence and its reverse complement were incorporated into oligonucleotides flanked by overhangs compatible with the combination of either PstI and BamHI (PB_top and PB_bottom, Key Resources Table), or SphI and SpeI (SS_top and SS_bottom, Key Resources Table), restriction enzymes. The appropriate pairs of oligonucleotides were synthesized (PAGE-purified; Integrated DNA Technologies), annealed, and cloned into the linearized vectors described above.

To generate the final starting material, the two plasmids with the cassettes for the introduction of DNA damage 3 kb or 4.5 kb left of ARS306 were digested with the blunt cutters NaeI (in the backbone of pBluescript II KS(–)) and ZraI (in the far left flank of the modified genomic DNA region) to excise approximately 1 kb DNA and the two blunt ends ligated to give a final plasmid size of approximately 9.7 kb. For the plasmid containing a cassette 4.5 kb from the origin, an additional two restriction enzyme sites, SacI and PsiI, were introduced approximately 1 kb and 1.6 kb respectively downstream of the cassette by site directed mutagenesis to facilitate characterization of the stalled fork. The final plasmids are hereafter referred to as ZN3 (plasmid with a cassette 3 kb from the origin) and ZN5SP1 (plasmid with a cassette 4.5 kb from the origin) and their sequences are given below:

ZN3 plasmid sequence

CTGACGCGCCCTGTAGCGGCGCATTAAGCGCGGCGGGTGTGGTGGTTACGCGCAGCGTGACCGCTACACTTGCCAGCGCCCTA
GCGCCCGCTCCTTTCGCTTTCTTCCCTTCCTTTCTCGCCACGTTCGCCGTCCTTCAATGAAACATCGTTGGCCACTAATTTGGCCA
GTGCAAAGTAGAACAAATCGGCAGCCTCCCAAGAAAGCTCCTTCTTACCCTTTGCCTCAGTCAGTTCTTCAGCTTCTTCCTTGATCT
TGGCATCTAACAATGCAGAGTCGTTGAATAGTCTTCTAGTATAAGATTCCTCTGGAGCGTCCTGTAGCCTTTGTTTTAGTAAAGATT
CTAGCCCCACCAAACCATGCTTGAATTCACCAAAGCAAGACATGGTCTCCAAGTGGCAAAATCCAACGTTTTCTTGTTCAACGATA
AACTTTAAGGCATCCGAATCACAGTCAGTAGAGATTTGTAAAAGCTTTTGGCCATTGCCAGAAGTTTCACCCTTGATCCAGATTTCA
TTCCTAGAACGAGAATAATAAACGCCACGACCCAATTCGATGGCCTTTGCTATAGATTTCTTCGAAGAATACACCAACCCTAGACA
ACGCTCATATTGGTCCACAACTAGGGTGGTATATAAACCGTCAGGACGGTCTGTACGTACTTCACCAAGCACTTCTTTGGTCAACA
TATCCTTGCTTAATTTCTTTATGGACACAATTTTATCTTGCGAGAATTTTTGTTTTACCATGAATTGATTGGAGAAAACACCGTTCTCT
TCCACAACAACACGCTCCTTTGGTACATTCAATTGTTCAACCAAGTGTTCGGCTGTTTTAGCATCTTGGCTTGCAATGAACAGAGAA
GAAACTCCGTTGTTCAAGAAGGCAATGATTTCATCATCGCTGAATTTACCACTTGGCAAGGACAAAGCCACCAATGGAACTTCTTC
CTCTTTGGAGAACTGGAGAATCTCTTCATTACTCAGGCTCGAGCCATCCAAAAGTACCTGACCAACAAGTGAAACGTATTCCTTCTT
ACTATTCCATGAGGCCAGATCATCAATTAACGGTAGAATCGGCAAAACCATTATTCAGAAAAAAAATTTTGTAAACTATTGTATTACT
ATTACACAGCGCAGTTGTGCTATGATATTAAAATGTATCCAGAACACACATCGGAGGTGAATATAACGTTCCATATCTATTATATACA
CAGTATACTACTGTTCATAGTCATATCCTTTTCTTACCTTCTATATCGAATGACTGATAATGCAACGTGAGTCACTGTGCATGGGTTT
AGCAATTATTAAACTAATTTACCGGAGTCACTATTAGAGTCAGTTCGACTGCCTAGAAGAACTGCTGGTTGTCAGGATTGTGATGGG
GGCATTCTGCTGTATTATGACCCATCGTATCGCAATGCTCACACCACTGTTGTCTTCCTGCCGTGGTATCGACTGGTGCAGGGGG
GTCGAAAATTGGCAACGATTCCACGGCTGTTTGTGCTTGAGCCTGTTCCAACTGTTTGAACCTTTCATTAGCCTCTTCAAGTTTTTT
CGTTAAGGATGCCACCTCTTCCGATGAGGAATCTTGTGGTTTTGTCAAAAATAGTTCCTTGCTCAAATTTTGGTATTCTTTACTGAGC
GAATCGTTATGCATTTTCAATTGTTCGCGTTCTTTAGCCCACTTTGTCTTGTGTAACTCAAATTGGTCTTCTATGTTGCGTAATTGTTC
CAGCTGTTTTTTCAGGAGTTCGACATCTTCGTTGGCACCAGTGGGTTGATTATGAGAAAGATTTCTCTCTTCGTTTTCTTTGATCTCT
TCGTGTAGTTGGCTTACGACAGCAAGTAGCTGTTCATTCTCAGCGTCAAAAAACTGCTTTTGTTTGGCTTGCTGTCTGCGTTCGAG
CAGACATTGTTGCTTGAGATGGTCTATCTCTTTCTCTCTTTCTTGTATTGTGGCTTCATACCTATCAAAAGTCGGTTGCACTTCTTCG
AGGACCATTCTTTGGTCATCGAGTAGCCTTTTGTAGTGTAGTTGTTTCCTTTGTAGCTTTTCGATGGTCAATTGGCGATCGCGTAATT

CAATTGTAACTTCGCTGCTATTGAGGTCATTCATGTGGCCATTGTCCGGTTTCCAATCGCTGGTGGTGTTGTGATTAGCCTTTCTGT
CTGATGACAGGATAGAGTCCACCTCCATTCTGTCTTCTCTGTTATCGTAACCAAATTCTTGCTGTTGATGGTGATCCGATGCCTCCT
GGTCCATCGACTGTTGATTACCGCTGTGCCGACTGGTGATCCGGAAACTTCTCATGGGTGTGGGGGATTTAGGATCATCCATGGG
AGAGAAGCGCTTAGTGAGCCTCACAATAGATCTGTTCACGGGTATTGATAGCGGTTCCATTGTCGTTCTTCTCGAGGTTTGCCATA
TCGGTCCGTTCTCGATCAATGATGCGACTTTTTGCAACTGAATAAATAGTCCACTTTGAGGATACTCCGTTTGAAAATACTTCTTCC
CCTAGGAATGATCCATCGTTCTTACCAATGTTGGCAAGTAAGTCTACACCAGCAAACATTCCACGCGTCGTGTCCACTGGACCCA
CGTATTTCAGTTGTCCGCGGCCGAAATTTGGGATTTGGTTTAAACATCCTATCTTTCTTTGATATCTATCCATGGTATATTAAGCGCA
TACGGCGCCAGCCACTAGTCAACGCCTTTTACCTTGTCCTTTGATGCATGCCTCGTCCAAACGTTTTTGGTGTCTTGGCCAATTGC
CCTTCTGAAAAATCTCACTGTCCGCAACTCATTAAAAGATACCCAAGCAAGCTACACGATAAAGAAAGGAGAAAGTTCATTACTGG
AACGTACATATAGCGATACAAACGTATAGCAAAGATCTGAAATGGATACGGATAAGTTAATCTCAGAGGCTGAGTCTCATTTTTCTC
AAGGAAACCATGCAGAAGCTGTTGCGAAGTTGACATCCGCAGCTCAGTCGAACCCCAATGACGAGCAAATGTCAACTATTGAATC
ATTAATTCAAAAAATCGCAGGATACGTCATGGACAACCGTAGTGGTGGTAGTGACGCCTCGCAAGATCGTGCTGCTGGTGGTGGT
TCATCTTTTATGAACACTTTAATGGCAGACTCTAAGGGTTCTTCCCAAACGCAACTAGGAAAACTAGCTTTGTTAGCCACAGTGATG
ACACACTCATCAAATAAAGGTTCTTCTAACAGAGGGTTTGACGTAGGGACTGTCATGTCAATGCTAAGTGGTTCTGGCGGCGGGA
GCCAAAGTATGGGTGCTTCCGGCCTGGCTGCCTTGGCTTCTCAATTCTTTAAGTCAGGTAACAATTCCCAAGGTCAGGGACAAGG
TCAAGGTCAAGGTCAAGGTCAAGGACAAGGTCAAGGTCAAGGTTCTTTTACTGCTTTGGCGTCTTTGGCTTCATCTTTCATGAATTC
CAACAACAATAATCAGCAAGGTCAAAATCAAAGCTCCGGTGGTTCCTCCTTTGGAGCACTGGCTTCTATGGCAAGCTCTTTTATGC
ATTCCAATAATAATCAGAACTCCAACAATAGTCAACAGGGCTATAACCAATCCTATCAAAACGGTAACCAAAATAGTCAAGGTTACA
ATAATCAACAGTACCAAGGTCGCGACGGTGGTTACCAACAACAACAGGGACAATCTGGTGGTGCTTTTTCCTCATTGGCCTCCAT
GGCTCAATCTTACTTAGGTGGTGGACAAACTCAATCCAACCAACAGCAATACAATCAACAAGGCCAAAACAACCAGCAGCAATAC
CAGCAACAAGGCCAAAACTATCAGCATCAACAACAGGGTCAGCAGCAGCAACAAGGCCACTCCAGTTCATTCTCAGCTTTGGCTT
CCATGGCAAGTTCCTACCTGGGCAATAACTCCAATTCAAATTCGAGTTATGTGTACACGCAACAGGCTAATGAGTATGGTAGACCG
CAACAGAATGGTCAACAGCAATCCAATGAGTACGGAAGACCGCAATACGGCGGAAACCAGAACTCCTAAGGACAGCACGAATCC
TTCAATTTTTCTGGCAACTTTTCTCAACAGAACAATAACGGCGCGCCGAACCGCTACTGAACGATGATTCAGTTCGCCTTCTATCC
TAAGTTTACGTATTTGCTAGCGCATATAACTTAGCGGGAAATTATTAATTGACCGGTAGGACAATTTTGTTGCACGTGATGCCTCAA
TCGTCTGCTTGCTTCCATAGTTAACATGAGGATCCGCAGTACCAACCTCAGCACTTAAGTCCTCAGCGCAGTACCAACTGCAGGAT
GCCCTTTTTGACGTATTGAATGGCATAATTGCACTGTCACTTTTCGCGCTGTCTCATTTTGGTGCGATGATGAAACTTTCATGAAAC
GTCTGTAATTTGAAACAAATAACGTAATTCTCGGGATTGGTTTTATTTAAATGACAATGTAAGAGTGGCTTTGTAAGGTATGTGTTGC
TCTTAAAATATTTGGATACGACATCCAAAATCTTTTTTCCTTTAAGAGCAGGATATAAGTCGACAAGTTTCTGAAAATCAAAATGGTA
GCAACAATAATGCAGACGACAACAACTGTGCTGACGACAGTCGCCGCAATGTCTACTACCTTAGCATCCCATTACATATCTTCGCA
AGCTAGTTCCTCGACGAGTGTAACAACAGTAACGACAATAGCGACATCAATACGCTCTACACCGTCTAATCTACTCTTTTCTAATGT
GGCGGCTCAGCCAAAATCATCTTCAGCAAGCACAATTGGGCTTTCAATCGGACTTCCCATCGGAATATTCTGTTTCGGATTACTTA
TCCTTTTGTGTTATTTCTACCTTAAAAGGAATTCGGTGTCCATTTCAAATCCACCCATGTCAGCTACGATTCCAAGGGAAGAGGAAT
ATTGTCGCCGCACTAATTGGTTCTCACGGTTATTTTGGCAGAGTAAGTGTGAGGATCAGAATTCATATTCTAATCGTGATATTGAGA
AGTATAACGACACCCAGTGGACCTCGGGTGATAACAAGTCTTCAAAAATACAGTACAAAATTTCCAAACCCATAATACCGCAGCAT
ATACTGACACCTAAGAAAACGGTGAAGAACCCATATGCTTGGTCTGGTAAAAACATTTCGTTAGACCCCAAAGTGAACGAAATGGA
GGAAGAGAAAGTTGTGGATGCATTCCTGTATACTAAACCACCGAATATTGTCCATATTGAATCCAGCATCCCCTCGTATAATGATTT
ACCTTCTCAAAAAACGGTGTCCTCAAAGAAAACTGCGTTAAAAACGAGTGAGAAATGGAGTTACGAATCTCCACTATCTCGATGGT
TCTTGAGGGGTTCTACATACTTTAAGGATTATGGCTTATCAAAGACCTCTTTAAAGACCCCAACTGGGGCTCCACAACTGAAGCAA
ATGAAAATGCTCTCCCGGATAAGTAAGGGTTACTTCAATGAGTCAGATATAATGCCTGACGAACGATCGCCCATCTTGGAGTATAA
TAACACGCCTCTGGATGCAAATGACAGTGTGAATAACTTGGGTAATACCACGCCAGATTCACAAATCACATCTTATCGCAACAATAA
CATCGATCTAATCACGGCAAGACCCCATTCAGTGATATACGGTACTACTGCACAACAAACTTTGGAAACCAACTTCAATGATCATC
ATGACTGCAATAAAAGCACTGAGAAACACGAGTTGATAATACCCACCCCATCAAAACCACTAAAGAAAAGGATATAAAGAAGACAA
AGTAAAATGTATCAGCATTTACAACATTTGTCACGTTCTAAACCATTGCCGCTTACTCCAAACTCCAAATATAATGGGGAGGCTTGC
GTCCAATTAGGGAAGACATATACAGTTATTCAGGATTACGAGCCTAGATTGACAGACGAAATAAGAATCTCGCTGGGTGAAAAAGT
TAAAATTCTGGCCACTCATACCGATGGATGGTGTCTGGTAGAAAAGTGTAATACACAAAAGGGTTCTATTCACGTCAGTGTTGACG
ATAAAAGATACCTCAATGAAGATAGAGGCATTGTGCCTGGTGACTGTCTCCAAGAATACGACTGATGAAAATAATATTGACGTTCG
CATTTAATCTATACCTATAATTCTGTACTTATATACTGTTCCTTAATTGAAGATTTCAACATCGTTTTTGATGTAGGTCTTTTCACCTGG
AGGTGCGGCTGGGCTACCGAAGACTAATTGAGCTTGTACGGTCCAAGACTCAGGGATTTTGCTTGGCAAAGCAGCTTTTATGTAA
CCATTGTAGTGTTGTAGGTGACCACCCAGGCCCATTGCCTCCAAGGCAACCCACGAGTTGATTTGAGCGGCACCAGAGGTATGG
TCCGCGAAACTAGGGAATGCAGCTGCGTACGCTGGGAAGTCAGCCTTTAGCTTTTCAGTTACCTTGTCGTCGGTGAAGAAGATTA
CAGAACCAAAGGCCTCATCCCTTGCTGAAGCAGGCCTCTTTTGACCGGCAGGGCTTTCTATAGCCTTAGTCACTTCGTCCCAAAC
TTTTTTGTGAGTTTCACCAGTCAAGATAACAGCGCGATTTGGCTGGGAGTTGAAAGCGGTGGGTGTGGCTCGAATGATGGTTTGGA
CGACGGATTGGATGTCGTTGATAGTAATTTCACCAGGTAACTCCGGTTTCAAAGCGTAAATAGTACGACGAGCAGTTAAAGTTTTCA
AATAAGTTGCAACAGCAGACATGATATTGGATTGCCGGAATGGCGATATGTTGATCCCGGATACTTCAGTCTACGAAAAAAGTACA
AATTATGTAGTCAGTTCCTTCAGTATGGTGTCCTTATATACTGTAGTTTGGACAAGGTGCAAATGCCAAGACCCTAGCCCGAAAAGC
TCGAGGCACCCCAGGATCTTCCCCTTTACGTAATTTTCACGTAAAACGCCACAGTCCGATTTTTCTCGAATAATCATTAGTAAAAGC
GGTATACTGGATTATTGTACGATAACAAGGTAGAGCTTTATTACTAAGCTAAGACGTTCTTACATCAATAGTGCTGTTCGTTATTGAT

GTTAGGAGAAGGAGCGGGTCTGGTGAATAGTGTAAGCAGTGTTTCTGAACTTTTTCTTCGTCTAAGTCCTTGTAATGTAAGGTAAG
AATGCAAGCATCTTGTTTGTAACCCGGGTGTACGTTGACGTTAGTAAGTCACAAACCCAAGCTTAACTTCTTCGTGAGGAAGGAAA
GTGTTGTCTCCTACTTTTTTCAAATTTTCGAATTGTATTTATATTTATTTAGTACTTCTTGAGTTTACATATCCTTCGTAAAAATGCAAC
TTTTGTCGAAAAACACTTCCAAAAAAAAATAATAATGAATTTATGAAGCATACTAACGAGCGAGCACATCGCTGACCTATCATTACTT
CATGAGATAAATTAAGATCTCCTCATATGCGAATTTCCTGTTCAGTGATAAACGTTGATTACGTTATTGATAAAAGTCTTTTCTTCTGG
CAAGGGGTACCCAGCTTTTGTTCCCTTTAGTGAGGGTTAATTGCGCGCTTGGCGTAATCATGGTCATAGCTGTTTCCTGTGTGAAA
TTGTTATCCGCTCACAATTCCACACAACATACGAGCCGGAAGCATAAAGTGTAAAGCCTGGGGTGCCTAATGAGTGAGCTAACTCA
CATTAATTGCGTTGCGCTCACTGCCCGCTTTCCAGTCGGGAAACCTGTCGTGCCAGCTGCATTAATGAATCGGCCAACGCGCGG
GGAGAGGCGGTTTGCGTATTGGGCGCTCTTCCGCTTCCTCGCTCACTGACTCGCTGCGCTCGGTCGTTCGGCTGCGGCGAGCG
GTATCAGCTCACTCAAAGGCGGTAATACGGTTATCCACAGAATCAGGGGATAACGCAGGAAAGAACATGTGAGCAAAAGGCCAG
CAAAAGGCCAGGAACCGTAAAAAGGCCGCGTTGCTGGCGTTTTTCCATAGGCTCCGCCCCCCTGACGAGCATCACAAAAATCGA
CGCTCAAGTCAGAGGTGGCGAAACCCGACAGGACTATAAAGATACCAGGCGTTTCCCCCTGGAAGCTCCCTCGTGCGCTCTCCT
GTTCCGACCCTGCCGCTTACCGGATACCTGTCCGCCTTTCTCCCTTCGGGAAGCGTGGCGCTTTCTCATAGCTCACGCTGTAGGT
ATCTCAGTTCGGTGTAGGTCGTTCGCTCCAAGCTGGGCTGTGTGCACGAACCCCCCGTTCAGCCCGACCGCTGCGCCTTATCCG
GTAACTATCGTCTTGAGTCCAACCCGGTAAGACACGACTTATCGCCACTGGCAGCAGCCACTGGTAACAGGATTAGCAGAGCGA
GGTATGTAGGCGGTGCTACAGAGTTCTTGAAGTGGTGGCCTAACTACGGCTACACTAGAAGGACAGTATTTGGTATCTGCGCTCT
GCTGAAGCCAGTTACCTTCGGAAAAAGAGTTGGTAGCTCTTGATCCGGCAAACAAACCACCGCTGGTAGCGGTGGTTTTTTTGTTT
GCAAGCAGCAGATTACGCGCAGAAAAAAAGGATCTCAAGAAGATCCTTTGATCTTTTCTACGGGGTCTGACGCTCAGTGGAACGA
AAACTCACGTTAAGGGATTTTGGTCATGAGATTATCAAAAAGGATCTTCACCTAGATCCTTTTAAATTAAAAATGAAGTTTTAAATCAA
TCTAAAGTATATATGAGTAAACTTGGTCTGACAGTTACCAATGCTTAATCAGTGAGGCACCTATCTCAGCGATCTGTCTATTTCGTTC
ATCCATAGTTGCCTGACTCCCCGTCGTGTAGATAACTACGATACGGGAGGGCTTACCATCTGGCCCCAGTGCTGCAATGATACCG
CGAGACCCACGCTCACCGGCTCCAGATTTATCAGCAATAAACCAGCCAGCCGGAAGGGCCGAGCGCAGAAGTGGTCCTGCAAC
TTTATCCGCCTCCATCCAGTCTATTAATTGTTGCCGGGAAGCTAGAGTAAGTAGTTCGCCAGTTAATAGTTTGCGCAACGTTGTTGC
CATTGCTACAGGCATCGTGGTGTCACGCTCGTCGTTTGGTATGGCTTCATTCAGCTCCGGTTCCCAACGATCAAGGCGAGTTACA
TGATCCCCCATGTTGTGCAAAAAAGCGGTTAGCTCCTTCGGTCCTCCGATCGTTGTCAGAAGTAAGTTGGCCGCAGTGTTATCACT
CATGGTTATGGCAGCACTGCATAATTCTCTTACTGTCATGCCATCCGTAAGATGCTTTTCTGTGACTGGTGAGTACTCAACCAAGTC
ATTCTGAGAATAGTGTATGCGGCGACCGAGTTGCTCTTGCCCGGCGTCAATACGGGATAATACCGCGCCACATAGCAGAACTTTA
AAAGTGCTCATCATTGGAAAACGTTCTTCGGGGCGAAAACTCTCAAGGATCTTACCGCTGTTGAGATCCAGTTCGATGTAACCCAC
TCGTGCACCCAACTGATCTTCAGCATCTTTTACTTTCACCAGCGTTTCTGGGTGAGCAAAAACAGGAAGGCAAAATGCCGCAAAAA
AGGGAATAAGGGCGACACGGAAATGTTGAATACTCATACTCTTCCTTTTTCAATATTATTGAAGCATTTATCAGGGTTATTGTCTCAT
GAGCGGATACATATTTGAATGTATTTAGAAAAATAAACAAATAGGGGTTCCGCGCACATTTCCCCGAAAAGTGCCAC

***ZN5SP1* plasmid sequence**

CTGACGCGCCCTGTAGCGGCGCATTAAGCGCGGCGGGTGTGGTGGTTACGCGCAGCGTGACCGCTACACTTGCCAGCGCCCTA
GCGCCCGCTCCTTTCGCTTTCTTCCCTTCCTTTCTCGCCACGTTCGCCGTCCTTCAATGAAACATCGTTGGCCACTAATTTGGCCA
GTGCAAAGTAGAACAAATCGGCAGCCTCCCAAGAAAGCTCCTTCTTACCCTTTGCCTCAGTCAGTTCTTCAGCTTCTTCCTTGATC
TTGGCATCTAACAATGCAGAGTCGTTGAATAGTCTTCTAGTATAAGATTCCTCTGGAGCGTCCTGTAGCCTTTGTTTTAGTAAAGATT
CTAGCCCCACCAAACCATGCTTGAATTCACCAAAGCAAGACATGGTCTCCAAGTGGCAAAATCCAACGTTTTCTTGTTCAACGATA
AACTTTAAGGCATCCGAATCACAGTCAGTAGAGATTTGTAAAAGCTTTTGGCCATTGCCAGAAGTTTCACCCTTGATCCAGATTTCA
TTCCTAGAACGAGAATAATAAACGCCACGACCCAATTCGATGGCCTTTGCTATAGATTTCTTCGAAGAATACACCAACCCTAGACA
ACGCTCATATTGGTCCACAACTAGGGTGGTATATAAACCGTCAGGACGGTCTGTACGTACTTCACCAAGCACTTCTTTGGTCAACA
TATCCTTGCTTAATTTCTTTATGGACACAATTTTATCTTGCGAGAATTTTTGTTTTACCATGAATTGATTGGAGAAAACACCGTTCTCT
TCCACAACAACACGCTCCTTTGGTACATTCAATTGTTCAACCAAGTGTTCGGCTGTTTTAGCATCTTGGCTTGCAATGAACAGAGAA
GAAACTCCGTTGTTCAAGAAGGCAATGATTTCATCATCGCTGAATTTACCACTTGGCAAGGACAAAGCCACCAATGGAACTTCTTC
CTCTTTGGAGAACTGGAGAATCTCTTCATTACTCAGGCTCGAGCCATCCAAAAGTACCTGACCAACAAGTGAAACGTATTCCTTCT
TACTATTCCATGAGGCCAGATCATCAATTAACGGTAGAATCGGCAAAACCATTATTCAGAAAAAAAATTTTGTAAACTATTGTATTAC
TATTACACAGCGCAGTTGTGCTATGATATTATAATGTATCCAGAACACACATCGGAGGTGAATATAACGTTCCATATCTATTATATAC
ACAGTATACTACTGTTCATAGTCATATCCTTTTCTTACCTTCTATATCGAATGACTGATAATGCAACGTGAGTCACTGTGCATGGGTT
TAGCAATTATTAAACTAATTTACCGGAGTCACTATTAGAGTCAGTTCGACTGCCTAGAAGAACTGCTGGTTGTCAGGATTGTGATGG
GGGCATTCTGCTGTATTATGACCCATCGTATCGCAATGCTCACACCACTGTTGTCTTCCTGCCGTGGTATCGACTGGTGCAGGGG
GGTCGAAAATTGGCAACGATTCCACGGCTGTTTGTGCTTGAGCCTGTTCCAACTGTTTGAACCTTTCATTAGCCTCTTCAAGTTTTT
TCGTTAAGGATGCCACCTCTTCCGATGAGGAATCTTGTGGTTTTGTCAAAAATAGTTCCTTGCTCAAATTTTGGTATTCTTTACTGAG
CGAATCGTTATGCATTTTCAATTGTTCGCGTTCTTTAGCCCACTTTGTCTTGTGTAACTCAAATTGGTCTTCTATGTTGCGTAATTGTT
CCAGCTGTTTTTTCAGGAGCTCGACATCTTCGTTGGCACCAGTGGGTTGATTATGAGAAAGATTTCTCTCTTCGTTTTCTTTGATCTC
TTCGTGTAGTTGGCTTACGACAGCAAGTAGCTGTTCATTCTCAGCGTCAAAAAACTGCTTTTGTTTGGCTTGCTGTCTGCGTTCGAG
CAGACATTGTTGCTTGAGATGGTCTATCTCTTTCTCTCTTTCTTGTATTGTGGCTTCATACCTATCAAAAGTCGGTTGCACTTCTTCG
AGGACCATTCTTTGGTCATCGAGTAGCCTTTTGTAGTGTAGTTGTTTCCTTTGTAGCTTTTCGATGGTCAATTGGCGATCGCGTAATT
CAATTGTAACTTCGCTGCTATTGAGGTCATTCATGTGGCCATTGTCCGGTTTCCAATCGCTGGTGGTGTTGTGATTAGCCTTTCTGT
CTGATGACAGGATAGAGTCCACCTCCATTCTGTCTTCTCTGTTATCGTAACCAAATTCTTGCTGTTGATGGTGATCCGATGCCTCCT

GGTCCATCGACTGTTGATTACCGCTGTGCCGACTGGTGATCCGGAAACTTCTCATGGGTGTGGGGGATTTAGGATCATCCATGGG
AGAGAAGCGCTTAGTGAGCCTCACAATAGATCTGTTCACGGGTATTGATAGCGGTTCCATTGTCGTTCTTCTCGAGGTTTGCCATA
TCGGTCCGTTCTCGATCAATGATGCGACTTTTTGCAACTGAATAAATAGTCCACTTTGAGGATACTCCGTTTGAAAATACTTCTTCC
CCTAGGAATGATCCATCGTTCTTACCAATGTTGGCAAGTAAGTCTACACCAGCAAACATTCCACGCGTCGTGTCCACTGGACCCA
CGTATTTCAGTTGTCCGCGGCCGAAATTTGGGATTTGGTTTAAACATCCTATCTTTCTTTGATATCTATCCATGGTATATTAAGCGCA
TACGGCGCCAGCCACTAGTGCAGTACCAACCTCAGCACTTAAGTCCTCAGCGCAGTACCAAGCATGCCTCGTCCAAACGTTTTTG
GTGTCTTGGCCAATTGCCCTTCTGAAAAATCTCACTGTCCGCAACTCATTAAAAGATACCCAAGCAAGCTACACGATAAAGAAAGG
AGAAAGTTCATTACTGGAACGTACATATAGCGATACAAACGTATAGCAAAGATCTGAAATGGATACGGATAAGTTAATCTCAGAGGC
TGAGTCTCATTTTTCTCAAGGAAACCATGCAGAAGCTGTTGCGAAGTTGACATCCGCAGCTCAGTCGAACCCCAATGACGAGCAA
ATGTCAACTATTGAATCATTAATTCAAAAAATCGCAGGATACGTCATGGACAACCGTAGTGGTGGTAGTGACGCCTCGCAAGATCG
TGCTGCTGGTGGTGGTTCATCTTTTATGAACACTTTAATGGCAGACTCTAAGGGTTCTTCCCAAACGCAACTAGGAAAACTAGCTTT
GTTAGCCACAGTGATGACACACTCATCAAATAAAGGTTCTTCTAACAGAGGGTTTGACGTAGGGACTGTCATGTCAATGCTAAGTG
GTTCTGGCGGCGGGAGCCAAAGTATGGGTGCTTCCGGCCTGGCTGCCTTGGCTTCTCAATTCTTTAAGTCAGGTAACAATTCCCA
AGGTCAGGGACAAGGTCAAGGTCAAGGTCAAGGTCAAGGACAAGGTCAAGGTCAAGGTTCTTTTACTGCTTTGGCGTCTTTGGCT
TCATCTTTCATGAATTCCAACAACAATAATCAGCAAGGTCAAAATCAAAGCTCCGGTGGTTCCTCCTTTGGAGCACTGGCTTCTATG
GCAAGCTCTTTTATGCATTCCAATAATAATCAGAACTCCAACAATAGTCAACAGGGCTATAACCAATCCTATCAAAACGGTAACCAA
AATAGTCAAGGTTACAATAATCAACAGTACCAAGGTCGCGACGGTGGTTACCAACAACAACAGGGACAATCTGGTGGTGCTTTTTC
CTCATTGGCCTCCATGGCTCAATCTTACTTAGGTGGTGGACAAACTCAATCCAACCAACAGCAATACAATCAACAAGGCCAAAACA
ACCAGCAGCAATACCAGCAACAAGGCCAAAACTATCAGCATCAACAACAGGGTCAGCAGCAGCAACAAGGCCACTCCAGTTCATT
CTCAGCTTTGGCTTCCATGGCAAGTTCCTACCTGGGCAATAACTCCAATTCAAATTCGAGTTATGTGTACACGCAACAGGCTAATG
AGTATGGTAGACCGCAACAGAATGGTCAACAGCAATCCAATGAGTACGGAAGACCGCAATACGGCGGAAACCAGAACTCCTAAG
GACAGCACGAATCCTTCAATTTTTCTGGCAACTTTTCTCAACAGAACAATAACGGCGCGCCGAACCGCTACTGAACGATGATTCAG
TTCGCCTTCTATCCTAAGTTTACGTATTTGCTAGCGCATATAACTTAGCGGGAAATTATTAATTGACCGGTAGGACAATTTTGTTGCA
CGTGATGCCTCAATCGTCTGCTTGCTTCCATAGTTAACATGAGGATCCTTTCAAAACAGAGTTGTATCTCTGCAGGATGCCCTTTTT
GACGTATTGAATGGCATAATTGCACTGTCACTTTTCGCGCTGTCTCATTTTGGTGCGATGATGAAACTTTCATGAAACGTCTGTAATT
TGAAACAAATAACGTAATTCTCGGGATTGGTTTTATTTAAATGACAATGTAAGAGTGGCTTTGTAAGGTATGTGTTGCTCTTAAAATAT
TTGGATACGACATCCAAAATCTTTTTTCCTTTAAGAGCAGGATATAAGTCGACAAGTTTCTGAAAATCAAAATGGTAGCAACAATAAT
GCAGACGACAACAACTGTGCTGACGACAGTCGCCGCAATGTCTACTACCTTAGCATCCCATTACATATCTTCGCAAGCTAGTTCCT
CGACGAGTGTAACAACAGTAACGACAATAGCGACATCAATACGCTCTACACCGTCTAATCTACTCTTTTCTAATGTGGCGGCTCAG
CCAAAATCATCTTCAGCAAGCACAATTGGGCTTTCAATCGGACTTCCCATCGGAATATTCTGTTTCGGATTACTTATCCTTTTGTGTT
ATTTCTACCTTAAAAGGAATTCGGTGTCCATTTCAAATCCACCCATGTCAGCTACGATTCCAAGGGAAGAGGAATATTGTCGCCGC
ACTAATTGGTTCTCACGGTTATTTTGGCAGAGTAAGTGTGAGGATCAGAATTCATATTCTAATCGTGATATTGAGAAGTATAACGACA
CCCAGTGGACCTCGGGTGATAACAAGTCTTCAAAAATACAGTACAAAATTTCCAAACCCATAATACCGCAGCATATACTGACACCT
AAGAAAACGGTGAAGAACCCATATGCTTGGTCTGGTAAAAACATTTCGTTAGACCCCAAAGTGAACGAAATGGAGGAAGAGAAAG
TTGTGGATGCATTCCTGTATACTAAACCACCGAATATTGTCCATATTGAATCCAGCATCCCCTCGTATAATGATTTACCTTCTCAAAA
AACGGTGTCCTCAAAGAAAACTGCGTTAAAAACGAGTGAGAAATGGAGTTACGAATCTCCACTATCTCGATGGTTCTTGAGGGGTT
CTACATACTTTAAGGATTATGGCTTATCAAAGACCTCTTTAAAGACCCCAACTGGGGCTCCACAACTGAAGCAAATGAAAATGCTCT
CCCGGATAAGTAAGGGTTACTTCAATGAGTCAGATATAATGCCTGACGAACGATCGCCCATCTTGGAGTATAATAACACGCCTCTG
GATGCAAATGACAGTGTGAATAACTTGGGTAATACCACGCCAGATTCACAAATCACATCTTATCGCAACAATAACATCGATCTAATC
ACGGCAAGACCCCATTCAGTGATATACGGTACTACTGCACAACAAACTTTGGAAACCAACTTCAATGATCATCATGACTGCAATAA
AAGCACTGAGAAACACGAGTTGATAATACCCACCCCATCAAAACCACTAAAGAAAAGGATATAAAGAAGACAAAGTAAAATGTATC
AGCATTTACAACATTTGTCACGTTCTAAACCATTGCCGCTTACTCCAAACTCCAAATATAATGGGGAGGCTTGCGTCCAATTAGGG
AAGACATATACAGTTATTCAGGATTACGAGCCTAGATTGACAGACGAAATAAGAATCTCGCTGGGTGAAAAAGTTAAAATTCTGGCC
ACTCATACCGATGGATGGTGTCTGGTAGAAAAGTGTAATACACAAAAGGGTTCTATTCACGTCAGTGTTGACGATAAAAGATACCT
CAATGAAGATAGAGGCATTGTGCCTGGTGACTGTCTCCAAGAATACGACTGATGAAAATAATATTGACGTTCGCATTTAATCTATAC
CTATAATTCTGTACTTATATACTGTTCCTTAATTGAAGATTTCAACATCGTTTTTGATGTAGGTCTTTTCACCTGGAGGTGCGGCTGG
GCTACCGAAGACTAATTGAGCTTGTACGGTCCAAGACTCAGGGATTTTGCTTGGCAAAGCAGCTTTTATGTAACCATTGTAGTGTT
GTAGGTGACCACCCAGGCCCATTGCCTCCAAGGCAACCCACGAGTTGATTTGAGCGGCACCAGAGGTATGGTCCGCGAAACTA
GGGAATGCAGCTGCGTACGCTGGGAAGTCAGCCTTTAGCTTTTCAGTTACCTTGTCGTCGGTGAAGAAGATTACAGAACCAAAGG
CCTCATCCCTTGCTGAAGCAGGCCTCTTTTGACCGGCAGGGCTTTCTATAGCCTTAGTCACTTCGTCCCAAACTTTTTTGTGAGTTT
CACCAGTCAAGATAACAGCGCGATTTGGCTGGGAGTTGAAAGCGGTGGGTGTGGCTCGAATGATGGTTTGGACGACGGATTGGA
TGTCGTTGATAGTAATTTCACCAGGTAACTCCGGTTTCAAAGCGTAAATAGTACGACGAGCAGTTAAAGTTTTCAAATAAGTTGCAA
CAGCAGACATGATATTGGATTGCCGGAATGGCGATATGTTGATCCCGGATACTTCAGTCTACGAAAAAAGTACAAATTATGTAGTC
AGTTCCTTCAGTATGGTGTCCTTATATACTGTAGTTTGGACAAGGTGCAAATGCCAAGACCCTAGCCCGAAAAGCTCGAGGCACC
CCAGGATCTTCCCCTTTACGTAATTTTCACGTAAAACGCCACAGTCCGATTTTTCTCGAATAATCATTAGTAAAAGCGGTATACTGG
ATTATTGTACGATAACAAGGTAGAGCTTTATTACTAAGCTAAGACGTTCTTACATCAATAGTGCTGTTCGTTATTGATGTTAGGAGAA
GGAGCGGGTCTGGTGAATAGTGTAAGCAGTGTTTCTGAACTTTTTCTTCGTCTAAGTCCTTGTAATGTAAGGTAAGAATGCAAGCAT
CTTGTTTGTAACCCGGGTGTACGTTGACGTTAGTAAGTCACAAACCCAAGCTTAACTTCTTCGTGAGGAAGGAAAGTGTTGTCTCC

TACTTTTTCAAATTTTCGAATTGTATTTATATTTATTTAGTACTTCTTGAGTTTACATATCCTTCGTAAAAATGCAACTTTTGTCGAAAA
ACACTTCCAAAAAAAAATAATAATGAATTTATGAAGCATACTAACGAGCGAGCACATCGCTGACCTATCATTACTTCATGAGATAAAT
TAAGATCTCCTCATATGCGAATTTCCTGTTCAGTGATAAACGTTGATTACGTTATTGATAAAAGTCTTTTCTTCTGGCAAGGGGTACC
CAGCTTTTGTTCCCTTTAGTGAGGGTTAATTGCGCGCTTGGCGTAATCATGGTCATAGCTGTTTCCTGTGTGAAATTGTTATCCGCT
CACAATTCCACACAACATACGAGCCGGAAGCATAAAGTGTAAAGCCTGGGGTGCCTAATGAGTGAGCTAACTCACATTAATTGCGT
TGCGCTCACTGCCCGCTTTCCAGTCGGGAAACCTGTCGTGCCAGCTGCATTAATGAATCGGCCAACGCGCGGGGAGAGGCGGT
TTGCGTATTGGGCGCTCTTCCGCTTCCTCGCTCACTGACTCGCTGCGCTCGGTCGTTCGGCTGCGGCGAGCGGTATCAGCTCAC
TCAAAGGCGGTAATACGGTTATCCACAGAATCAGGGGATAACGCAGGAAAGAACATGTGAGCAAAAGGCCAGCAAAAGGCCAGG
AACCGTAAAAAGGCCGCGTTGCTGGCGTTTTTCCATAGGCTCCGCCCCCCTGACGAGCATCACAAAAATCGACGCTCAAGTCAG
AGGTGGCGAAACCCGACAGGACTATAAAGATACCAGGCGTTTCCCCCTGGAAGCTCCCTCGTGCGCTCTCCTGTTCCGACCCTG
CCGCTTACCGGATACCTGTCCGCCTTTCTCCCTTCGGGAAGCGTGGCGCTTTCTCATAGCTCACGCTGTAGGTATCTCAGTTCGG
TGTAGGTCGTTCGCTCCAAGCTGGGCTGTGTGCACGAACCCCCCGTTCAGCCCGACCGCTGCGCCTTATCCGGTAACTATCGTC
TTGAGTCCAACCCGGTAAGACACGACTTATCGCCACTGGCAGCAGCCACTGGTAACAGGATTAGCAGAGCGAGGTATGTAGGCG
GTGCTACAGAGTTCTTGAAGTGGTGGCCTAACTACGGCTACACTAGAAGGACAGTATTTGGTATCTGCGCTCTGCTGAAGCCAGTT
ACCTTCGGAAAAAGAGTTGGTAGCTCTTGATCCGGCAAACAAACCACCGCTGGTAGCGGTGGTTTTTTTGTTTGCAAGCAGCAGAT
TACGCGCAGAAAAAAAGGATCTCAAGAAGATCCTTTGATCTTTTCTACGGGGTCTGACGCTCAGTGGAACGAAAACTCACGTTAAG
GGATTTTGGTCATGAGATTATCAAAAAGGATCTTCACCTAGATCCTTTTAAATTAAAAATGAAGTTTTAAATCAATCTAAAGTATATAT
GAGTAAACTTGGTCTGACAGTTACCAATGCTTAATCAGTGAGGCACCTATCTCAGCGATCTGTCTATTTCGTTCATCCATAGTTGCC
TGACTCCCCGTCGTGTAGATAACTACGATACGGGAGGGCTTACCATCTGGCCCCAGTGCTGCAATGATACCGCGAGACCCACGC
TCACCGGCTCCAGATTTATCAGCAATAAACCAGCCAGCCGGAAGGGCCGAGCGCAGAAGTGGTCCTGCAACTTTATCCGCCTCC
ATCCAGTCTATTAATTGTTGCCGGGAAGCTAGAGTAAGTAGTTCGCCAGTTAATAGTTTGCGCAACGTTGTTGCCATTGCTACAGG
CATCGTGGTGTCACGCTCGTCGTTTGGTATGGCTTCATTCAGCTCCGGTTCCCAACGATCAAGGCGAGTTACATGATCCCCCATG
TTGTGCAAAAAAGCGGTTAGCTCCTTCGGTCCTCCGATCGTTGTCAGAAGTAAGTTGGCCGCAGTGTTATCACTCATGGTTATGGC
AGCACTGCATAATTCTCTTACTGTCATGCCATCCGTAAGATGCTTTTCTGTGACTGGTGAGTACTCAACCAAGTCATTCTGAGAATA
GTGTATGCGGCGACCGAGTTGCTCTTGCCCGGCGTCAATACGGGATAATACCGCGCCACATAGCAGAACTTTAAAAGTGCTCATC
ATTGGAAAACGTTCTTCGGGGCGAAAACTCTCAAGGATCTTACCGCTGTTGAGATCCAGTTCGATGTAACCCACTCGTGCACCCAA
CTGATCTTCAGCATCTTTTACTTTCACCAGCGTTTCTGGGTGAGCAAAAACAGGAAGGCAAAATGCCGCAAAAAAGGGAATAAGGG
CGACACGGAAATGTTGAATACTCATACTCTTCCTTTTTCAATATTATTGAAGCATTTATCAGGGTTATTGTCTCATGAGCGGATACAT
ATTTGAATGTATTTAGAAAAATAAACAAATAGGGGTTCCGCGCACATTTCCCCGAAAAGTGCCAC

Introduction of site-specific DNA damage

Oligonucleotides for undamaged DNA (AflII_undamaged; PAGE-purified; Integrated DNA Technologies) or DNA containing an abasic site (AflII_idSp; PAGE-purified; Integrated DNA Technologies) or CPD (AflII_CPD; HPLC-purified; TriLink Biotechnologies) were synthesized and purified (sequences: Key Resources Table). All oligonucleotides were stored in 10 mM Tris-HCl (pH 8.0), 1 mM EDTA at –20°C.

To introduce these oligonucleotides into the template, 4 × 160 μg of the relevant plasmid were digested each in a total volume of 400 μL (400 ng/μl DNA concentration) with 21 μL Nt.BbvCI (210 U; New England Biolabs R0632) at 37°C for 2 h. A further 5 μL Nt.BbvCI (50 U) were then added per tube and digested for a further 1 h. Complete nicking to open circular DNA from covalently closed DNA was confirmed by agarose gel electrophoresis in the presence of ethidium bromide (Figure S1B, lanes 1 and 2). Reactions were stopped by adding EDTA to 50 mM and stored at –20°C overnight. The DNA was thawed and competitor oligonucleotide (AflII_competitor; desalt purification; Integrated DNA Technologies) (sequence: Key Resources Table), containing a region complementary to the nicked fragment, added to 1000-fold molar excess over plasmid concentration (27 μL from a 1 mM stock per tube). The mix was incubated at 50°C for 20 min, then transferred to 37°C and SDS added to 0.1%. After 5 min, 1/100 volumes of proteinase K (New England Biolabs P8107S) was added and incubated at 37°C for a further 15 min. All tubes were pooled and the gapped plasmid purified from oligonucleotides by loading onto a Sepharose 4B (Sigma-Aldrich 4B200) gel filtration matrix equilibrated in 5 mM Tris-HCl (pH 8.0), 0.1 mM EDTA in a siliconized 1 m x 1 cm econo-column (Biorad 7371091). The column was run under gravity flow and after 14 mL void volume, 2-3 mL fractions collected. The gapped plasmid fractions were pooled (approximately 12 mL), split into 0.5 mL fractions and frozen overnight. Fractions were concentrated approximately 10-fold in a vacuum concentrator (ScanVac ScanSpeed 40 Centrifuge). The DNA was pooled and the concentration measured. This typically yielded 380-580 μg DNA (60%–90% input material).

100-150 μg gapped DNA were collected per oligonucleotide ligation. AflII_undamaged or AflII_idSp oligonucleotides were added at 10-fold molar excess, or AflII_CPD oligonucleotide was added at 20-fold molar excess. The mix was incubated at 50°C for 15 min then allowed to cool slowly to room temperature (Figure S1B, lanes 3, 5, 7). Ligation was performed at 300-500 ng/μl plasmid concentration in 1X T4 DNA ligase buffer (New England Biolabs B0202S), 0.5 mM ATP, 2 mM $Mg(OAc)_2$ with 110 U T4 DNA ligase (New England Biolabs M0202M) per μg plasmid at 16°C overnight in the dark (Figure S1B, lanes 4, 6, 8). The following day, SDS (to 0.1%) and proteinase K (1/100 volumes) were added and incubated at 37°C for 20 min. The DNA was extracted with phenol:chloroform:isoamyl alcohol 25:24:1 saturated with TE (Sigma-Aldrich P2069) and the aqueous phase dialysed against 2 L TE for 2 h. The sample volume was made up to 500 μL with TE and was mixed with 100 μL ~1% ethidium bromide (Sigma-Aldrich 46067) and 0.6 g caesium chloride (CsCl) and loaded onto a CsCl gradient in TE (density 1 g CsCl / 1 mL TE) in OptiSeal polypropylene

centrifuge tubes (13 × 48 mm; 4.9 mL) (Beckman Coulter 362185). The tubes were spun in an NVT 90 rotor (Beckman Coulter) at 75,000 rpm for 3 h then 65,000 rpm for 2 h using an Optima LE-80K Ultracentrifuge (Beckman Coulter). The lower band (ligated material) was collected and extracted with five changes of CsCl-saturated TE/isopropanol in the dark to remove ethidium bromide. The DNA was dialyzed against three changes of 2 L TE over 24 h total in a D-Tube Dialyzer Midi, MWCO 6-8 kDa (Merck 71507) at 4°C in the dark to remove all traces of CsCl (Figure S1B, lanes 9, 11, 13). The DNA was collected, frozen in dry ice and concentrated 5-10 fold in a vacuum concentrator, before precipitating with 0.3 M NaCl + 2.8 volumes ice cold 100% ethanol in dry ice. The pellet was harvested, washed with room temperature 70% ethanol, harvested, air-dried and resuspended in 50 μL TE. This protocol typically yielded DNA at 500-700 ng/μl from 100 μg gapped plasmid ligation (25%–35%). Digests with APE 1 (New England Biolabs M0282S) revealed complete and specific cleavage of plasmids with an abasic site oligonucleotide (AflII_idSp) integrated, validating the efficiency of this method for introducing DNA damage (Figure S1B, lanes 10, 12, 14).

Linearized templates for standard assays

Most experiments on undamaged and damaged DNA were performed on templates prepared by Nt.BbvCI nicking then annealing and ligation of the appropriate oligonucleotide following by CsCl gradient purification as described above, with the following exceptions. Undamaged templates in Figures 1B (“maxi” lanes), 2B, 2E, 5B, 5C, 6D, 6E, S1E, S2B, S3A, S3B, S5C, and S5D were from maxi prep DNA without CsCl gradient purification. Undamaged templates in Figures 1F and 3E were from maxi prep DNA followed by CsCl gradient purification. Replication of undamaged templates prepared in these ways gave comparable results (Figure 1B).

All replication assays were performed on linearized templates. Most assays were performed on templates linearized with a single enzyme, AhdI (for studying a leading strand template CPD and undamaged controls) or AleI (for studying a lagging strand template CPD and undamaged controls), with the following exceptions. In Figure 1B, lanes 5-8, a BamHI-linearized undamaged template was used to demonstrate origin specificity. In the following figures, templates were linearized with both AhdI and a second restriction enzyme (listed below) to reduce the length of leftward moving replicon: Figure 6E: PsiI; Figures 5B, 5C, S5C, and S5D (brackets: run off position): EagI (A) (104 nt), AfeI (B) (376 nt), SacI (C) (973 nt) or PsiI (D) (1575 nt). The templates for Figures 5B, 5C, S5C, and S5D are shown in Figure S5B for both damaged and undamaged forms, and the replicated part containing the origin and the unreplicated fragment are indicated.

Template linearization was performed in all cases by mixing 8-10 μg plasmid DNA in 50 μL final reaction volume containing 1X CutSmart Buffer (New England Biolabs B7204), 2.5 μL each restriction enzyme (all New England Biolabs) and incubating at 37°C for 3 h. The DNA was extracted with phenol:chloroform:isoamyl alcohol 25:24:1 saturated with TE (Sigma-Aldrich P2069) and the aqueous phase collected and DNA precipitated with 0.3 M NaCl + 2.8 volumes ice cold 100% ethanol in dry ice. The pellet was harvested, washed with room temperature 70% ethanol, harvested, air-dried and resuspended in 15-20 μL TE for use in replication assays.

Linearized templates immobilized on beads

Undamaged (maxi prep) and CPD-containing plasmid DNA were linearized with SapI restriction enzyme (New England Biolabs R0569), which cuts approximately 0.5 kb right of ARS306. Biotinylated oligonucleotides (SapI_Btn_1 and SapI_Btn_2; PAGE-purified; Sigma-Aldrich) (sequences: Key Resources Table) for coupling to M-280 Steptavidin Dynabeads (ThermoFisher Scientific 11205D) were designed to anneal to the SapI sticky ends on the end of the template closest to the origin. These oligonucleotides were mixed (10 μM each) in 0.2 M NaCl, 25 mM EDTA and annealed (85°C for 5 min, then cooled slowly to room temperature). SapI-linearized plasmid (100 ng/μl, 15 nM) and 40-fold molar excess ligated oligonucleotides (600 nM) were mixed in 1X T4 DNA ligase buffer (New England Biolabs B0202S), 0.5 mM ATP, 5 mM $Mg(OAc)_2$ with 233 U T4 DNA ligase (New England Biolabs M0202M) per μg plasmid at 16°C overnight in the dark. The following day, SDS (to 0.1%) and proteinase K (1/100 volumes) were added and incubated at 37°C for 20 min. The DNA was extracted with phenol:chloroform:isoamyl alcohol 25:24:1 saturated with TE (Sigma-Aldrich P2069) and the aqueous phase collected. Ligated template DNA was isolated from oligonucleotides by passing the aqueous phase over Sephacryl S-400 High Resolution matrix (GE Healthcare 17-0609-10) columns (prepared by applying 800 μL 50% slurry to 0.8 mL Pierce Centrifuge Columns (Thermo Scientific 89868) and washing 2 × 250 μL TE by centrifuging at 700 *g* for 1 min). 35-40 μL was applied per column and collected by centrifuging at 700 *g* for 2 min. DNA was pooled, frozen, lyophilized in a vacuum concentrator and resuspended to 250-500 ng/μl in TE.

To immobilize the DNA, 160 μL Dynabeads slurry stock were washed three times with 100 μL buffer A (10 mM Tris-HCl (pH 7.5), 2 M NaCl, 1 mM EDTA) then mixed with 4 μg DNA + buffer A to a total volume of 80 μl, and incubated at 25°C for 30 min, shaking at 500 rpm. The supernatant was discarded and the beads washed with 2 × 100 μL buffer B (10 mM HEPES-KOH (pH 7.6), 1 M KCl, 1 mM EDTA), 2 × 100 μL buffer C (10 mM HEPES-KOH (pH 7.6), 1 mM EDTA), resuspended in 75 μL buffer C and stored at 4°C in the dark.

Molecular weight markers

Molecular weight standards were prepared by first dephosphorylating 12.5 μg (25 μl) λ DNA-HindIII Digest (New England Biolabs) with 10 U Antarctic Phosphatase (New England Biolabs M0289S) in total volume 30 μL at 37°C for 1 h. The DNA was then purified using a QIAquick PCR Purification Kit (QIAGEN) and eluted in 30 μL water. 1 μg of this DNA was labeled with γ-[^{32}P]-ATP using 10 U T4 Polynucleotide Kinase (New England Biolabs M0201S) in total volume 20 μL at 37°C for 1 h. The DNA was extracted with phenol:chloroform:isoamyl alcohol 25:24:1 saturated with TE (10 mM Tris-HCl (pH 8.0), 1 mM EDTA) (Sigma-Aldrich P2069) and the buffer exchanged and unincorporated nucleotides were removed from the aqueous phase with illustra MicroSpin G-50 columns (GE Healthcare). These markers were run as size standards in all gels and cropped from the final image for presentation.

Standard replication assays

MCM loading and phosphorylation was performed by incubating 5 nM linearized DNA template, 5 mM ATP, 75 nM Cdt1/Mcm2-7, 45 nM Cdc6, 20 nM ORC, 50 nM DDK in 25 mM HEPES-KOH (pH 7.6), 100 mM potassium glutamate, 0.01% NP-40-S, 1 mM DTT, 10 mM Mg(OAc)$_2$, 0.1 mg/ml BSA, 40 mM KCl at 24°C for 10 min. S-CDK was then added to 120 nM and incubation continued for a further 5 min. An appropriate volume of the mixture was collected such that it would be diluted 6-fold in the final reaction mix. This was added to a mixture of pre-equilibrated buffer to give a final replication reaction buffer of 29.2 mM HEPES-KOH (pH 7.6), 217 mM potassium glutamate (except where indicated), 0.0117% NP-40-S, 1.17 mM DTT, 11.7 mM Mg(OAc)$_2$, 0.117 mg/ml BSA, 6.7 mM KCl, 3 mM ATP, 400 μM CTP, GTP, UTP, 30 μM dATP, dCTP, dGTP, dTTP, 33 nM α-[^{32}P]-dCTP, 12.5 nM Cdt1/Mcm2-7, 7.5 nM Cdc6, 3.3 nM ORC, 8.3 nM DDK, 20 nM S-CDK. In reactions omitting Pol δ, 117 mM potassium glutamate was used in all experiments (Yeeles et al., 2017), except in Figures S3D and 5D (217 mM). Replication was initiated by adding a cocktail of proteins to give final concentrations (unless otherwise stated in figures) of 30 nM Dpb11, 210 nM GINS, 40 nM Cdc45, 20 nM Pol ε, 5 nM Mcm10, 20 nM Ctf4, 60 nM RPA, 20 nM Csm3/Tof1, 20 nM Mrc1, 20 nM RFC, 20 nM PCNA, 10 nM TopoI, 20 nM Pol α, 5 nM Pol δ, 25 nM Sld3/7, 50 nM Sld2, and the mix was transferred to 30°C. Where necessary, protein stocks were diluted in 25 mM HEPES-KOH (pH 7.6), 300 mM potassium acetate, 10% glycerol, 0.02% NP-40-S, 0.5 mM EDTA, 1 mM DTT. We estimate the additional contribution from protein storage buffers to the final reaction buffer conditions to be ~15 mM chloride and ~25 mM acetate/glutamate, and corresponding sodium/potassium counterions. For protein titration experiments, the protein in question was added to the desired concentration immediately before addition of the protein cocktail. For pulse-chase experiments, unlabeled deoxyribonucleotide concentrations were adjusted to 30 μM dATP, dTTP, dGTP and 2.5 μM dCTP in the pulse phase. The chase was then performed as follows: Figures 1F, 5F and S5G: at 2 min 50 s (Figure 1F) or 4 min 50 s (Figures 5F and S5G) from the start, nucleotide concentrations were adjusted to 400 μM dATP, dCTP, dGTP, dTTP; Figures 3E and 3F: at 14 min 50 s from the start, nucleotide concentrations were adjusted to 100 μM dATP, dCTP, dGTP, dTTP. For most experiments, reactions were quenched at the desired time point by addition of EDTA to 25-30 mM. Oligonucleotides (sequences: Key Resources Table) were added to 60 nM (molecules) where indicated.

Post-reaction sample processing

For reactions not requiring post-quenching restriction enzyme digests or native gel analysis, unincorporated nucleotides were removed from the quenched reaction mix with illustra MicroSpin G-50 columns (GE Healthcare), which also exchanged the buffer to TE. Samples were supplemented with 20 mM EDTA, and 1/10 volume alkaline loading dye (0.5 M NaOH, 10% sucrose, xylene cyanol in water) added. For all other reactions, SDS (to 0.1%) and proteinase K (1/100 volumes) were added and incubated at 37°C for 20 min. The DNA was extracted with phenol:chloroform:isoamyl alcohol 25:24:1 saturated with TE (10 mM Tris-HCl (pH 8.0), 1 mM EDTA) (Sigma-Aldrich P2069) and the buffer exchanged and unincorporated nucleotides were removed from the aqueous phase with illustra MicroSpin G-50 columns (GE Healthcare). Except where indicated, samples for native gels were not digested with restriction enzymes, and mixed with native loading dye (20 mM Tris-HCl (pH 8.0), 50 mM EDTA, 10% Ficoll 400, 2% N-Lauroylsarcosine sodium salt solution) without additional processing. For restriction enzyme digests for the products of a standard 5-10 μL replication assay sample, 0.5-1 μL enzyme (all New England Biolabs) was added in 1X CutSmart (New England Biolabs B7204). Enzyme (2-4 μl) and buffer volumes were scaled up for larger samples for 2D gels. Digests were performed at 37°C for 30 min, except SmaI which was incubated at 25°C. Digests were quenched with EDTA, samples split where appropriate, and added to native or alkaline loading dye.

Denatured samples were analyzed in denaturing 0.6% agarose gels run at 24 V overnight in 30 mM NaOH, 2 mM EDTA. Native samples were analyzed in vertical 1% agarose gels run at 21-24 V overnight in modified 1X TAE (50 mM Tris-HCl (pH 7.9), 40 mM NaOAC, 1 mM EDTA (pH 8.0)), except for Figures S3A and S3B, which were 0.8% agarose. For standard two-dimensional gels, the sample was split, and a small analytical sample (2%–10%) loaded in one lane and the majority (90%–98%) loaded in another lane on the same gel. The latter lane was excised from the vertical native gel with a razor blade and laid horizontally along the top of a second denaturing gel and run at 27 V overnight. For two-dimensional gels with two native gel steps, a gel slice containing the preparative SmaI-digested full-length material was excised from the first native gel, its position indicted by an SmaI-/AhdI-linearized plasmid stained with ethidium bromide in a neighboring lane. A small analytical sample (2%–10%) was run in the same gel. The excised gel slice was broken up and put in a D-Tube Dialyzer Mini, MWCO 6-8 kDa (Merck 71504) and the DNA electroeluted with two changes of 70 μL modified 0.5X TAE (50 V, 45 min per elution). The eluted DNA was frozen in dry ice, concentrated approximately 4-fold in a vacuum concentrator and buffer exchanged by passing over an illustra MicroSpin G-50 column (GE Healthcare). 1X CutSmart and 1 μL BamHI (±1 μL PmlI, 1 μL AgeI-HF) were added and incubated at 37°C for 1 h. When DpnI was also included, 1 μL of a 1:25 dilution was added after this time and incubated for a further 5 min. Reactions were quenched with EDTA, treated with SDS and proteinase K and subsequently analyzed by the standard two-dimensional gel protocol described above.

Chromatin experiments

Template chromatinization

Chromatin was assembled on 3 nM (20 ng/μl) AhdI- or AleI-linearized DNA in 40 μL total reaction volume. Nap1 (3 μM), histone octamers (370 nM) and ISW1 (30 nM) were pre-incubated in chromatin assembly buffer (25 mM HEPES-KOH (pH 7.6), 10 mM Mg(OAc)$_2$,

100 mM KOAc, 0.1% NP-40-S, 5% glycerol, 0.1 mg/ml BSA) on ice for 10 min. ATP (3 mM), creatine phosphate (40 mM) and creatine phosphate kinase (140 μg/ml) were added and chromatin assembly initiated by addition of DNA and transferring the reactions to 30°C for 1 h.

Chromatin replication

To prepare chromatin for replication assays, chromatin was exchanged to replication assay buffer by applying 40 μL chromatin assembly reaction to Sephacryl S-400 High Resolution matrix (GE Healthcare 17-0609-10) columns (prepared by applying 800 μL 50% slurry to 0.8 mL Pierce Centrifuge Columns (Thermo Scientific 89868) and washing with 3 × 250 μL 25 mM HEPES-KOH (pH 7.6), 100 mM potassium glutamate, 0.01% NP-40-S, 1 mM DTT, 10 mM $Mg(OAc)_2$, 40 mM KCl) and collecting by centrifuging at 700 *g* for 2 min. For helicase loading and phosphorylation, a volume of chromatin corresponding to the total reaction volume less the contribution from ATP, BSA, Cdt1/Mcm2-7, Cdc6, ORC and DDK was used. Chromatin replication was otherwise performed as for naked DNA templates except where noted in the figures and with the following exceptions: Sld2 was used at 30 nM; Pol δ was used at 10 nM in Figure S1G; reactions were always performed at 117 mM potassium glutamate, since chromatin limits strand displacement by Pol δ (Devbhandari et al., 2017); all reactions also contained 40 nM FACT, 400 nM Nhp6A.

MNase assay

After chromatin assembly $CaCl_2$ was added to 5 mM. 20 μL was collected and 1 μL 20-fold diluted MNase (100 U) (New England Biolabs M0247, diluted in 1X MNase buffer, New England Biolabs B0247) added and the mixture incubated at 37°C for 5 min. The reaction was stopped with 20 mM EGTA. 5 volumes of buffer PB (from QIAquick PCR purification kit, QIAGEN) were added and the mixture applied to a QIAquick spin column, washed with buffer PE and eluted in 35 μL water. Products were analyzed by 1.5% agarose gel electrophoresis.

Replication assays on immobilized templates

Helicase loading and phosphorylation was performed by incubating equal volumes of DNA-bound Dynabead slurry in buffer C (prepared as described above) with a mixture containing reaction buffer components, proteins and ATP to give a final reaction mix as used for helicase loading and phosphorylation on a standard soluble template, but without KCl. After treatment with S-CDK as described above, an appropriate volume of the mixture was collected such that it would be diluted 5-fold in the final reaction mix. Replication was initiated as described above but in the absence of α-[^{32}P]-dCTP. In Figure 7E, the concentration of Pol α in the first phase was 5 nM. After incubating at 30°C for 15 min with gentle vortexing of the beads every 5 min, tubes were split into three equal volumes (3 × 15 μl) and the beads collected on a magnet. The reaction buffer was removed and replaced with an equal volume of the following reaction mix (or one supplemented with 60 nM RPA ± 60 nM re-priming oligonucleotide): 25 mM HEPES-KOH (pH 7.6), 100 mM potassium glutamate, 0.01% NP-40-S, 1 mM DTT, 10 mM $Mg(OAc)_2$, 0.1 mg/ml BSA, 3 mM ATP, 400 μM CTP, GTP, UTP, 30 μM dATP, dCTP, dGTP, dTTP, 66 nM α-[^{32}P]-dCTP, 20 nM Pol ε, 5 nM Mcm10, 20 nM Ctf4, 20 nM Csm3/Tof1, 20 nM Mrc1, 20 nM RFC, 20 nM PCNA, 10 nM TopoI, 20 nM Pol α. In Figure 7E, the concentration of Pol α in the second phase was varied as indicated. The beads were resuspended in the new reaction mix and replication continued at 30°C for 50 min further. After this time, in Figure 7B, each reaction was split into 2 × 7.5 μl: one was untreated and the other incubated with 1 μL AvrII (New England Biolabs) at 30°C for 10 min. Reactions were quenched with 25 mM EDTA, NaCl added to 1 M and the beads resuspended. Tubes were incubated at 25°C for 5 min before beads were collected on the magnet. For the tubes treated with AvrII, the supernatant was collected and passed over illustra MicroSpin G-50 columns (GE Healthcare), supplemented with 20 mM EDTA, and 1/10 volume alkaline loading dye (0.5 M NaOH, 10% sucrose, xylene cyanol in water) added. The beads fractions (±AvrII) were washed with 2 × 100 μL 1 M NaCl, 25 mM HEPES-KOH (pH 7.6), then resuspended in 10 μL 50 mM EDTA + 5 μL alkaline loading dye and incubated at 37°C for 1 h. In Figure 7E, the entire reaction was digested with AvrII and processed in the same way. Products were analyzed in denaturing 0.6% agarose gels as described above.

Primase assay

Primed template was prepared by annealing oligonucleotide JY180 (500 nM) (sequence: Key Resources Table) to M13mp18 ssDNA (50 nM) (New England Biolabs) in 10 mM Tris-Cl pH 7.6, 5 mM EDTA and 100 mM NaCl. The sample was heated to 75°C and slowly cooled to room temperature. Unannealed oligonucleotide was removed with an S400 column (GE Healthcare). Assays (10 μl) were performed at 30°C in a buffer containing 25 mM HEPES-KOH (pH 7.6), 100 mM potassium glutamate, 0.01% NP-40-S, 1 mM DTT, 10 mM $Mg(OAc)_2$, 0.1 mg/ml BSA, 3 mM ATP, 400 μM CTP, GTP, UTP, 30 μM dATP, dCTP, dGTP, dTTP, 33 nM α-[^{32}P]-dCTP, 20 nM Pol α and RPA (0-400 nM). RPA was first pre-bound to the template in the absence of Pol α for 10 min. Reactions were initiated by addition of Pol α and were incubated for 20 min. Samples were processed and run through alkaline agarose gels as described for soluble replication reactions.

Gel imaging and presentation

Denaturing gels were fixed with two changes of cold 5% trichloroacetic acid and dried onto chromatography paper (Whatman). Native gels were dried directly onto chromatography paper. Most gels were autoradiographed with Amersham Hyperfilm MP (GE Healthcare) for presentation. For quantification, gels were exposed on BAS-IP MS Storage Phosphor Screens (GE Healthcare) and screens were developed on a Typhoon phosphorimager (GE Healthcare).

QUANTIFICATION AND STATISTICAL ANALYSIS

Replication product quantification was performed in ImageJ (National Institutes of Health) after converting the .gel files to 16-Bit Tiff files using the Linearize GelData command. Lane profiles shown in Figures 6B, 6C, 7C, 7D, 7F, S7E, S7F, and S7G were generated on linearized files in ImageJ, and normalized to the intensity of the stall product in Figures 7C, 7D, and 7F. For quantification (Figure 1G) of maximum leading strand product length from denaturing gels (Figure 1F), lane profiles were extracted in ImageJ and the gel position at which the smoothed (10 neighbors) first derivative of each lane became > 1000 determined in GraphPad Prism. The corresponding position was converted to molecular weight in kb by comparison with molecular weight standards. Data of molecular weight as a function of time for four time points (3, 4, 5, 6 min) were fit to linear regressions in GraphPad Prism and the slope of the regression used to calculate replication rates.

In all cases, graphs of quantified data show the mean and errors (SEM) from n (stated in the figure legends) independent experimental repeats.

Molecular Cell, Volume *70*

Supplemental Information

The Initial Response of a Eukaryotic Replisome to DNA Damage

Martin R.G. Taylor and Joseph T.P. Yeeles

Figure S1

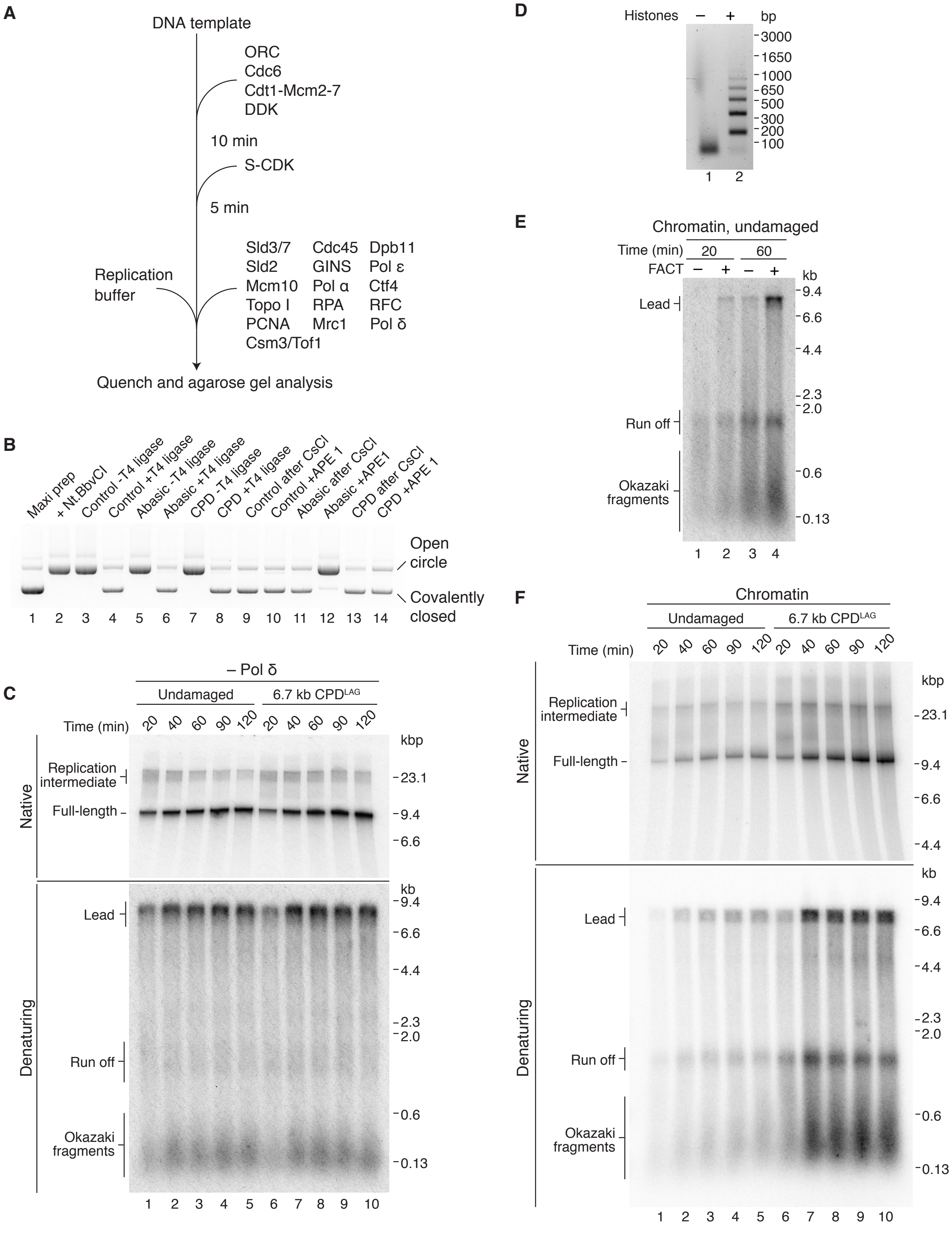
A
DNA template
ORC
Cdc6
Cdt1-Mcm2-7
DDK
10 min
S-CDK
5 min
Replication buffer
Sld3/7 Cdc45 Dpb11
Sld2 GINS Pol ε
Mcm10 Pol α Ctf4
Topo I RPA RFC
PCNA Mrc1 Pol δ
Csm3/Tof1
Quench and agarose gel analysis
B
Maxi prep
+ Nt.BbvCI
Control -T4 ligase
Control +T4 ligase
Abasic -T4 ligase
Abasic +T4 ligase
CPD -T4 ligase
CPD +T4 ligase
Control after CsCl
Control +APE 1
Abasic after CsCl
Abasic +APE1
CPD after CsCl
CPD +APE 1
Open circle
Covalently closed
1 2 3 4 5 6 7 8 9 10 11 12 13 14
C
– Pol δ
Undamaged
6.7 kb CPDLAG
Time (min) 20 40 60 90 120 20 40 60 90 120
Native
Replication intermediate
Full-length
kbp
23.1
9.4
6.6
Denaturing
Lead
Run off
Okazaki fragments
kb
9.4
6.6
4.4
2.3
2.0
0.6
0.13
1 2 3 4 5 6 7 8 9 10
D
Histones – +
bp
3000
1650
1000
650
500
300
200
100
1 2
E
Chromatin, undamaged
Time (min) 20 60
FACT – + – +
Lead
Run off
Okazaki fragments
kb
9.4
6.6
4.4
2.3
2.0
0.6
0.13
1 2 3 4
F
Chromatin
Undamaged
6.7 kb CPDLAG
Time (min) 20 40 60 90 120 20 40 60 90 120
Native
Replication intermediate
Full-length
kbp
23.1
9.4
6.6
4.4
Denaturing
Lead
Run off
Okazaki fragments
kb
9.4
6.6
4.4
2.3
2.0
0.6
0.13
1 2 3 4 5 6 7 8 9 10

Figure S2

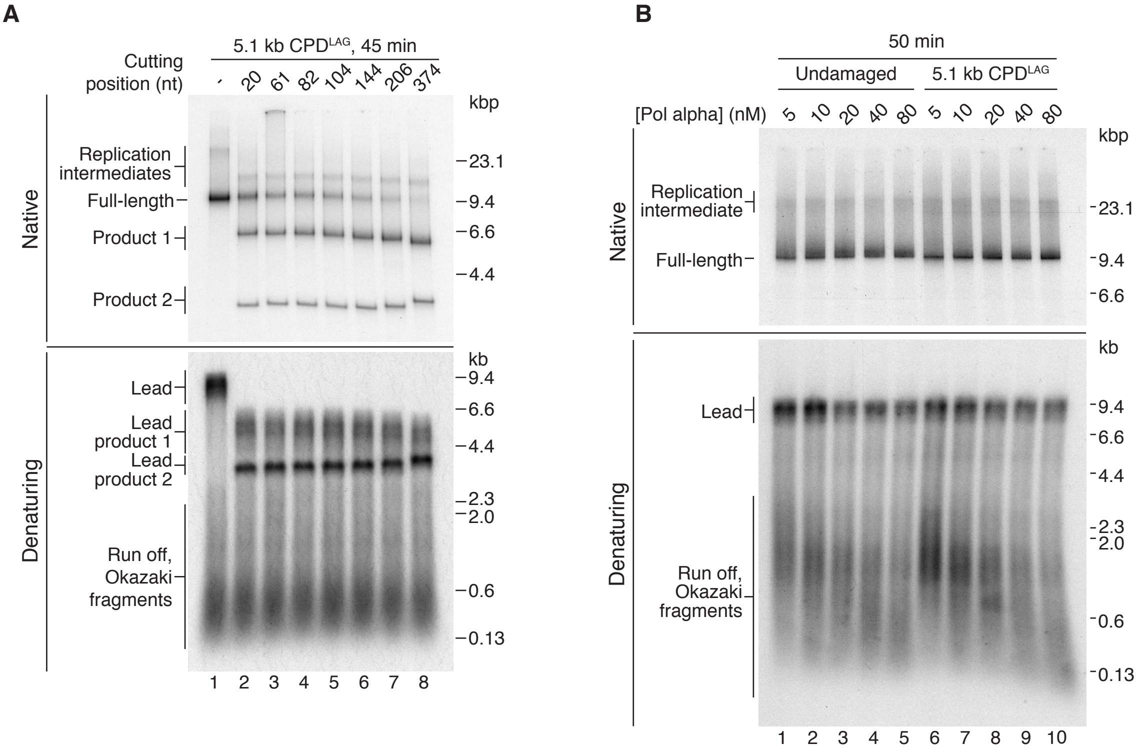

A
5.1 kb CPD^LAG, 45 min
Cutting position (nt)
-
20
61
82
104
144
206
374
kbp
Native
Replication intermediates
Full-length
Product 1
Product 2
23.1
9.4
6.6
4.4
kb
Denaturing
Lead
Lead product 1
Lead product 2
Run off, Okazaki fragments
9.4
6.6
4.4
2.3
2.0
0.6
0.13
1 2 3 4 5 6 7 8
B
50 min
Undamaged
5.1 kb CPD^LAG
[Pol alpha] (nM)
5 10 20 40 80 5 10 20 40 80
kbp
Native
Replication intermediate
Full-length
23.1
9.4
6.6
kb
Denaturing
Lead
Run off, Okazaki fragments
9.4
6.6
4.4
2.3
2.0
0.6
0.13
1 2 3 4 5 6 7 8 9 10

Figure S3

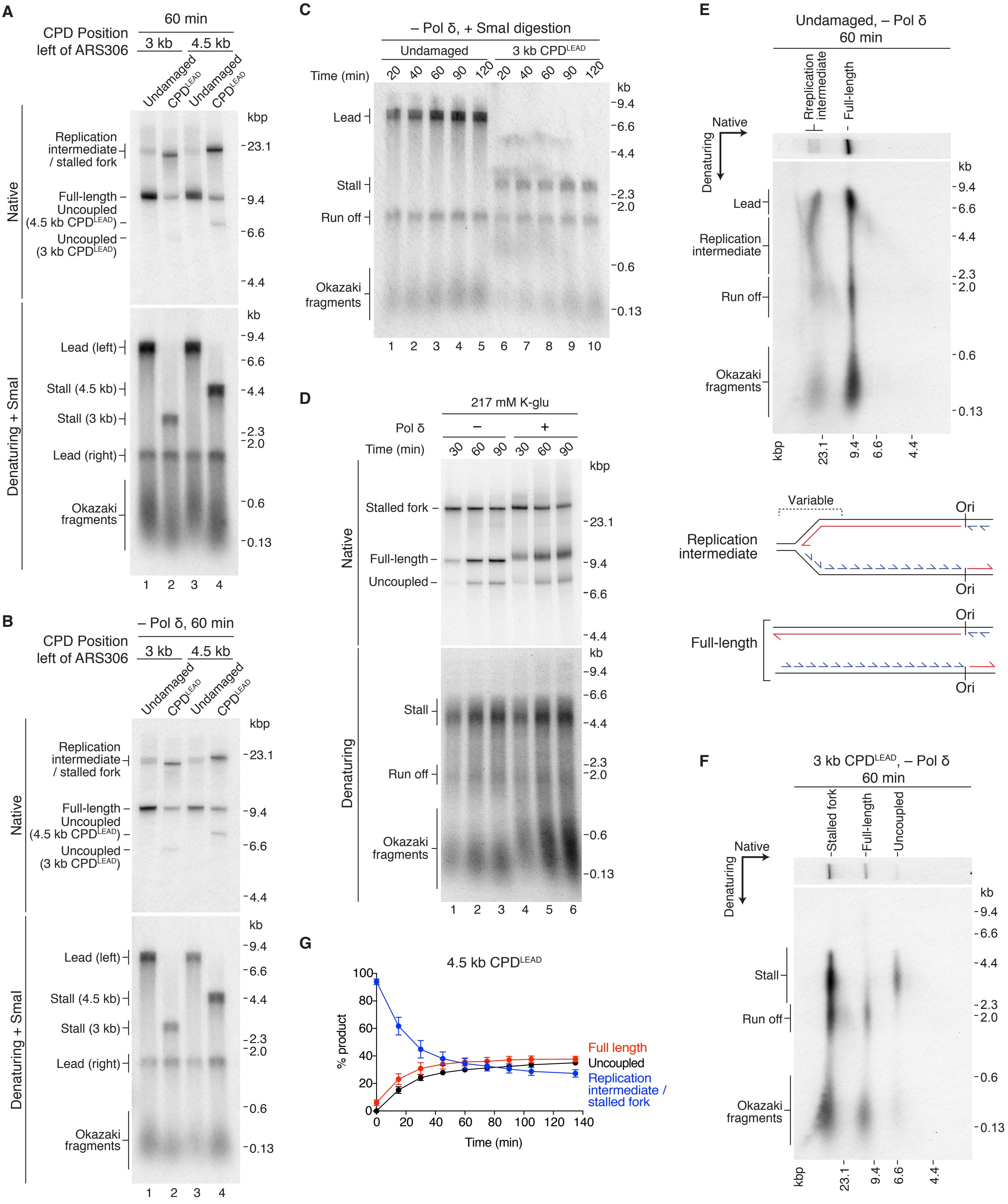
A
60 min
CPD Position left of ARS306
3 kb
4.5 kb
Undamaged
CPDLEAD
Native
Replication intermediate / stalled fork
Full-length
Uncoupled (4.5 kb CPDLEAD)
Uncoupled (3 kb CPDLEAD)
Denaturing + SmaI
Lead (left)
Stall (4.5 kb)
Stall (3 kb)
Lead (right)
Okazaki fragments
kbp
23.1
9.4
6.6
4.4
kb
2.3
2.0
0.6
0.13
B
– Pol δ, 60 min
C
– Pol δ, + SmaI digestion
Undamaged
3 kb CPDLEAD
Time (min)
Lead
Stall
Run off
D
217 mM K-glu
Pol δ
Stalled fork
Full-length
Uncoupled
Denaturing
E
Undamaged, – Pol δ
60 min
Replication intermediate
Full-length
Variable
Ori
F
3 kb CPDLEAD, – Pol δ
Stalled fork
G
4.5 kb CPDLEAD
% product
Time (min)
Full length
Uncoupled
Replication intermediate / stalled fork

Figure S4

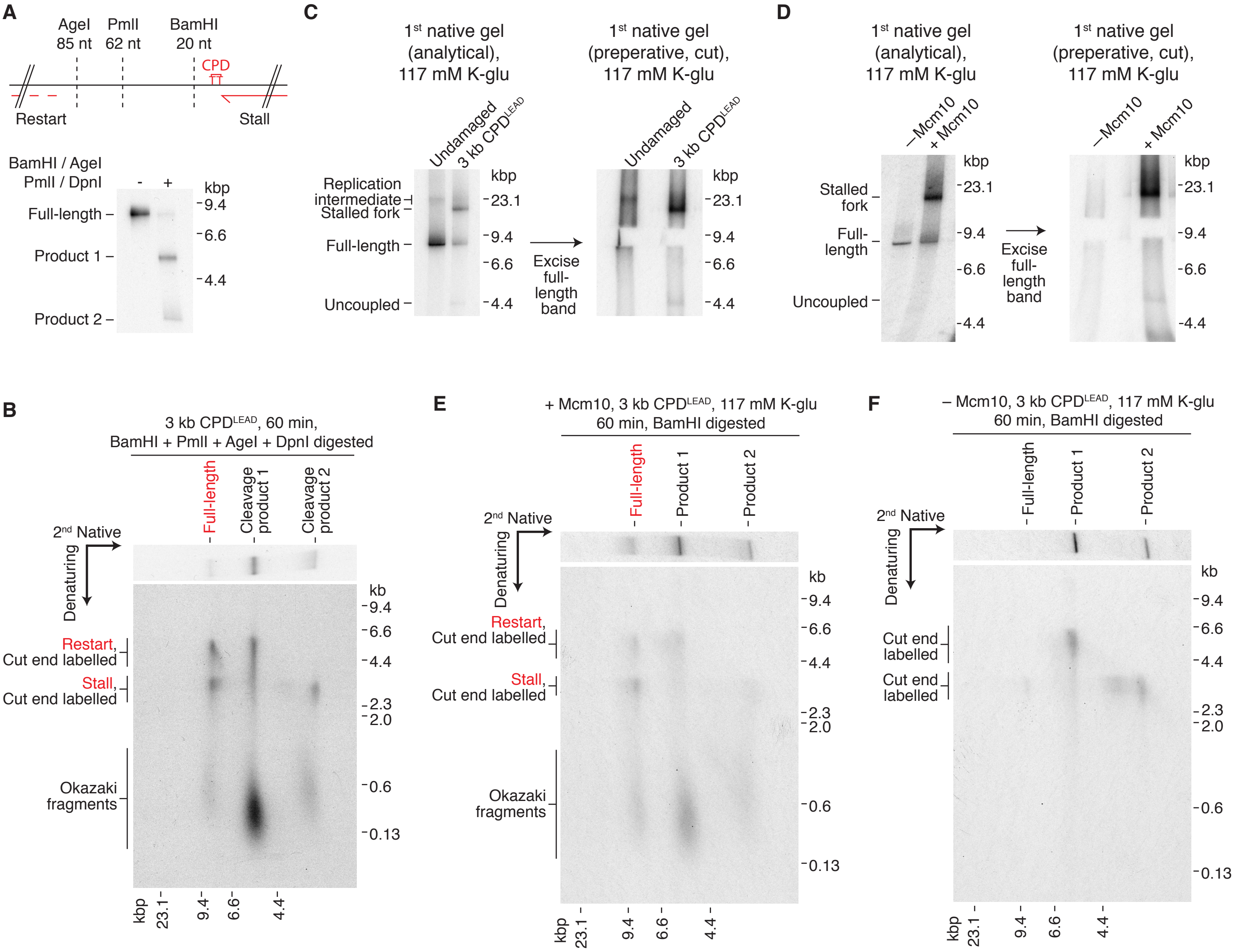
A
AgeI 85 nt
PmlI 62 nt
BamHI 20 nt
CPD
Restart
Stall
BamHI / AgeI
PmlI / DpnI
- +
kbp
Full-length
Product 1
Product 2
9.4
6.6
4.4
C
1st native gel (analytical), 117 mM K-glu
1st native gel (preperative, cut), 117 mM K-glu
Undamaged
3 kb CPD LEAD
Replication intermediate
Stalled fork
Full-length
Uncoupled
Excise full-length band
23.1
9.4
6.6
4.4
D
1st native gel (analytical), 117 mM K-glu
1st native gel (preperative, cut), 117 mM K-glu
– Mcm10
+ Mcm10
Stalled fork
Full-length
Uncoupled
Excise full-length band
B
3 kb CPD LEAD, 60 min, BamHI + PmlI + AgeI + DpnI digested
Full-length
Cleavage product 1
Cleavage product 2
2nd Native
Denaturing
Restart, Cut end labelled
Stall, Cut end labelled
Okazaki fragments
kb
2.3
2.0
0.6
0.13
E
+ Mcm10, 3 kb CPD LEAD, 117 mM K-glu 60 min, BamHI digested
Product 1
Product 2
F
– Mcm10, 3 kb CPD LEAD, 117 mM K-glu 60 min, BamHI digested
Cut end labelled

Figure S5

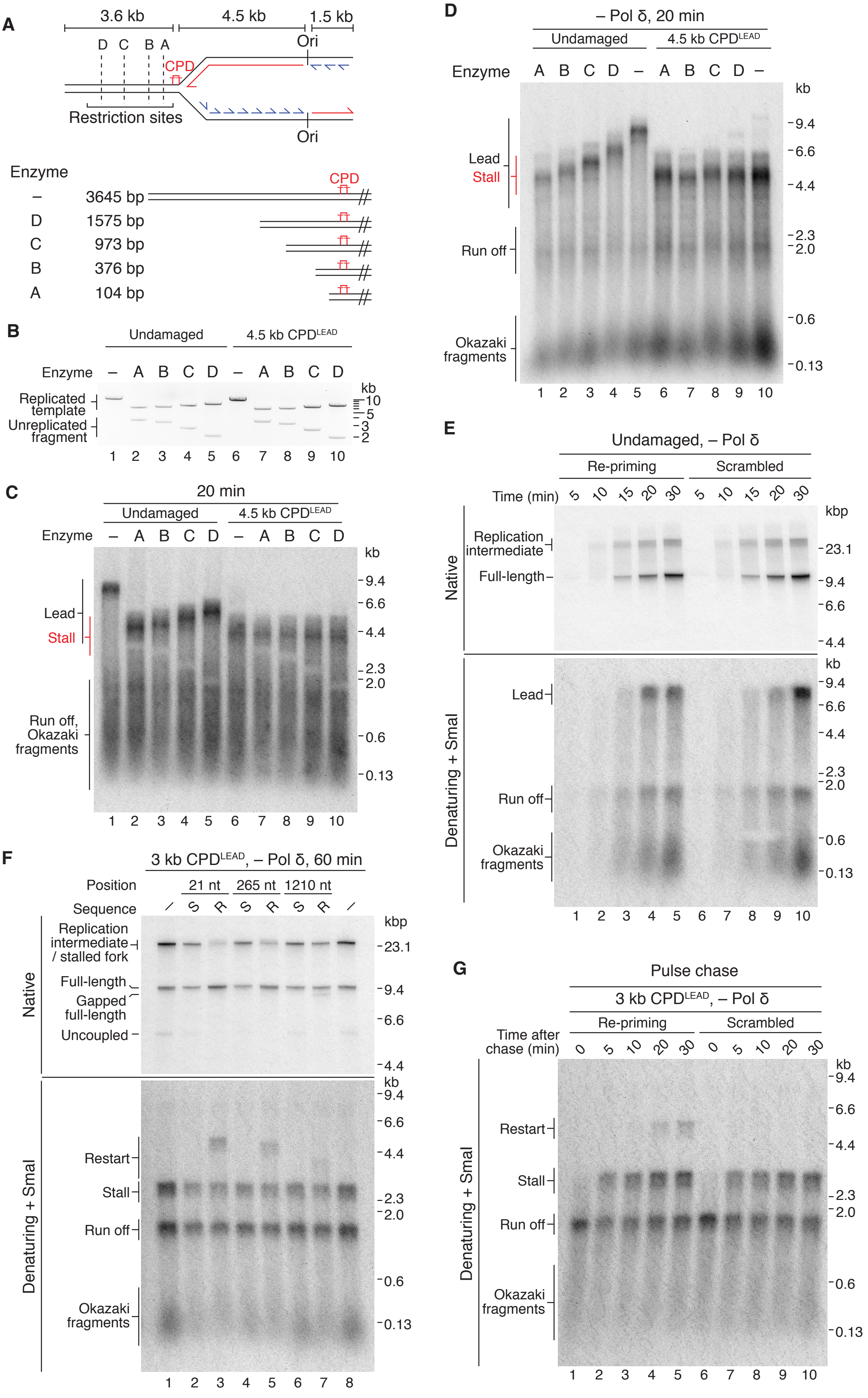

A
3.6 kb
4.5 kb
1.5 kb
D C B A
Ori
CPD
Restriction sites
Ori
Enzyme
– 3645 bp
D 1575 bp
C 973 bp
B 376 bp
A 104 bp
CPD
B
Undamaged
4.5 kb CPDLEAD
Enzyme – A B C D – A B C D
kb
Replicated template
Unreplicated fragment
10
5
3
2
1 2 3 4 5 6 7 8 9 10
C
20 min
Undamaged
4.5 kb CPDLEAD
Enzyme – A B C D – A B C D
kb
Lead
Stall
Run off, Okazaki fragments
9.4
6.6
4.4
2.3
2.0
0.6
0.13
1 2 3 4 5 6 7 8 9 10
D
– Pol δ, 20 min
Undamaged
4.5 kb CPDLEAD
Enzyme A B C D – A B C D –
kb
Lead
Stall
Run off
Okazaki fragments
9.4
6.6
4.4
2.3
2.0
0.6
0.13
1 2 3 4 5 6 7 8 9 10
E
Undamaged, – Pol δ
Re-priming
Scrambled
Time (min) 5 10 15 20 30 5 10 15 20 30
kbp
Native
Replication intermediate
Full-length
23.1
9.4
6.6
4.4
kb
Denaturing + SmaI
Lead
Run off
Okazaki fragments
9.4
6.6
4.4
2.3
2.0
0.6
0.13
1 2 3 4 5 6 7 8 9 10
F
3 kb CPDLEAD, – Pol δ, 60 min
Position
21 nt
265 nt
1210 nt
Sequence
kbp
Native
Replication intermediate / stalled fork
Full-length
Gapped full-length
Uncoupled
23.1
9.4
6.6
4.4
kb
Denaturing + SmaI
Restart
Stall
Run off
Okazaki fragments
9.4
6.6
4.4
2.3
2.0
0.6
0.13
1 2 3 4 5 6 7 8
G
Pulse chase
3 kb CPDLEAD, – Pol δ
Re-priming
Scrambled
Time after chase (min) 0 5 10 20 30 0 5 10 20 30
kb
Denaturing + SmaI
Restart
Stall
Run off
Okazaki fragments
9.4
6.6
4.4
2.3
2.0
0.6
0.13
1 2 3 4 5 6 7 8 9 10

Figure S6

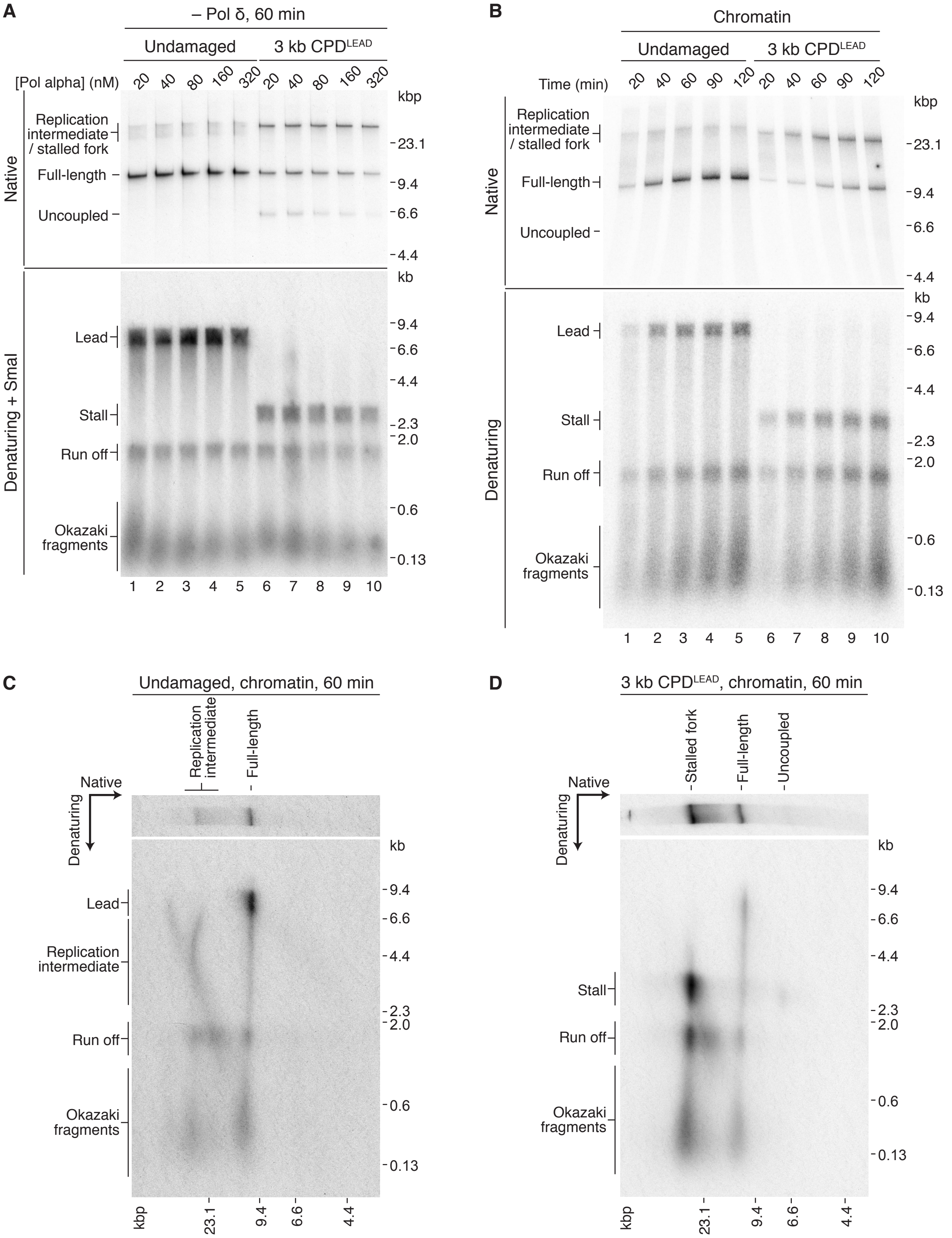

A
– Pol δ, 60 min
Undamaged
3 kb CPD^LEAD
[Pol alpha] (nM) 20 40 80 160 320 20 40 80 160 320
Native
Replication intermediate / stalled fork
Full-length
Uncoupled
kbp
23.1
9.4
6.6
4.4
Denaturing + SmaI
Lead
Stall
Run off
Okazaki fragments
kb
9.4
6.6
4.4
2.3
2.0
0.6
0.13
1 2 3 4 5 6 7 8 9 10
B
Chromatin
Undamaged
3 kb CPD^LEAD
Time (min) 20 40 60 90 120 20 40 60 90 120
Native
Replication intermediate / stalled fork
Full-length
Uncoupled
kbp
23.1
9.4
6.6
4.4
Denaturing
Lead
Stall
Run off
Okazaki fragments
kb
9.4
6.6
4.4
2.3
2.0
0.6
0.13
1 2 3 4 5 6 7 8 9 10
C
Undamaged, chromatin, 60 min
Replication intermediate
Full-length
Native
Denaturing
Lead
Replication intermediate
Run off
Okazaki fragments
kb
9.4
6.6
4.4
2.3
2.0
0.6
0.13
kbp 23.1 9.4 6.6 4.4
D
3 kb CPD^LEAD, chromatin, 60 min
Stalled fork
Full-length
Uncoupled
Native
Denaturing
Stall
Run off
Okazaki fragments
kb
9.4
6.6
4.4
2.3
2.0
0.6
0.13
kbp 23.1 9.4 6.6 4.4

Figure S7

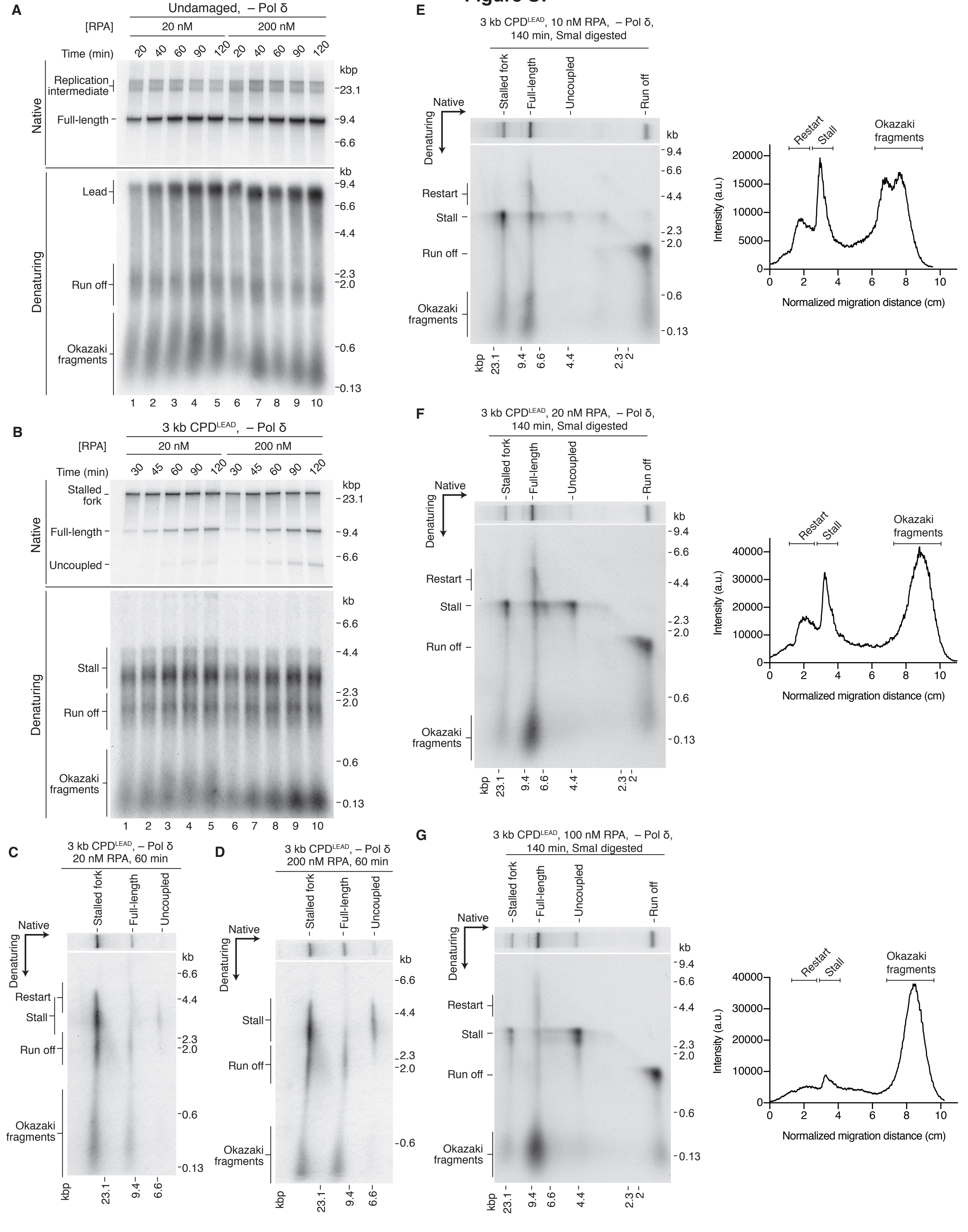

A
Undamaged, – Pol δ
[RPA]
20 nM
200 nM
Time (min)
20
40
60
90
120
kbp
Replication intermediate
23.1
Native
Full-length
9.4
6.6
kb
Lead
Denaturing
4.4
Run off
2.3
2.0
Okazaki fragments
0.6
0.13
1 2 3 4 5 6 7 8 9 10
B
3 kb CPD^LEAD, – Pol δ
30
45
Stalled fork
Uncoupled
Stall
C
3 kb CPD^LEAD, – Pol δ
20 nM RPA, 60 min
D
3 kb CPD^LEAD, – Pol δ
200 nM RPA, 60 min
Restart
E
3 kb CPD^LEAD, 10 nM RPA, – Pol δ,
140 min, SmaI digested
F
3 kb CPD^LEAD, 20 nM RPA, – Pol δ,
140 min, SmaI digested
G
3 kb CPD^LEAD, 100 nM RPA, – Pol δ,
140 min, SmaI digested
Intensity (a.u.)
Normalized migration distance (cm)

Figure S1. Related to Figure 1.

(A) Reaction scheme for standard replication reactions.

(B) Analysis of the different steps of replication template preparation. DNA products were separated through a 1% agarose gel in the presence of ethidium bromide. Lane 1: maxi prep starting material; lane 2: after nicking by Nt.BbvCI restriction endonuclease; lanes 3-8: after annealing undamaged, abasic, or CPD oligonucleotides before (lanes 3, 5, 7) and after (lanes 4, 6, 8) ligation by T4 DNA ligase; lanes 9, 11, 13: undamaged, abasic or CPD plasmids after caesium chloride gradient purification; lanes 10, 12, 14: undamaged, abasic or CPD plasmids after digestion with APE 1. BbvCI nicking and oligonucleotide ligation was performed at a site 3 kb left of ARS306 (Ori).

(C) Replication of undamaged and 6.7 kb CPD^{LAG} templates in the absence of Pol δ. In this and all subsequent experiments entirely lacking Pol δ the replication buffer contained 117 mM potassium glutamate. This potassium glutamate concentration was selected because: it supports efficient leading- and lagging-strand synthesis at rates that are comparable to those measured *in vivo* (Yeeles et al., 2017); it was not necessary to use a higher ionic strength buffer to suppress excessive Pol δ-dependent strand-displacement synthesis; we reasoned that for subsequent experiments with CPD^{LEAD} templates (Figures 3-7), the higher ionic strength (217 mM potassium glutamate) required to suppress Pol δ may not have been optimal for leading-strand re-priming.

(D) Micrococcal nuclease digest of a chromatinized undamaged template. Products were separated through a 1.5% native agarose gel and were visualized by ethidium bromide staining.

(E) FACT-dependent replication of a chromatinized undamaged template in the presence of 10 nM Pol δ.

(F) Replication of chromatinized undamaged and 6.7 kb CPD^{LAG} templates.

Figure S2. Related to Figure 2.

(A) Standard replication reaction on the 5.1 kb CPD^{LAG} template. Products were digested post-replicatively with enzymes mapping at the indicated positions from the CPD.

(B) Undigested replication reaction products for the experiment in Figure 2E.

Figure S3. Related to Figure 3.

(A and B) Replication reactions on CPD^{LEAD} templates performed in the presence (A) and absence (B) of Pol δ.

(C) Denaturing gel analysis of the replication reaction in Figure 3C following SmaI digestion.

(D) Replication reaction on the 4.5 kb CPD^{LEAD} template at 217 mM potassium glutamate showing that Pol δ does not influence uncoupled fork progression downstream of CPD^{LEAD}.

(E and F) Two-dimensional gel analysis of experiments performed on undamaged (E) and 3 kb CPD^{LEAD} (F) templates in the absence of Pol δ. Diagrams of the replication products from the undamaged template are illustrated in (E).
(G) Quantitation of pulse chase experiments on 4.5 kb CPD^{LEAD} templates as in Figure 3H, but with the respective contributions of full length and uncoupled products plotted individually (rather than summed as resolved products). Error bars represent the SEM from 4 experiments.

Figure S4. Related to Figure 4.
(A) Top: schematic of the predicted replication product of leading-strand lesion bypass by re-priming >85 nt from the CPD (3 kb CPD^{LEAD} template). The location of the BamHI, PmlI and AgeI restriction sites are illustrated. Bottom: electroeluted DNA from Figure 4B was digested with BamHI, PmlI, AgeI and DpnI and analyzed by native electrophoresis.
(B) Electroeluted DNA from Figure 4B was digested with BamHI, PmlI, AgeI and DpnI and analyzed by two-dimensional electrophoresis.
(C) Replication products from 60 min reactions containing 117 mM potassium glutamate on undamaged or 3 kb CPD^{LEAD} templates were quenched, deproteinized and digested with SmaI. Samples were then split and 3% of each was run in a native gel and processed as for standard reactions (analytical). The remaining 97% were run in separate lanes of the same native gel and the full-length bands were excised and electroeluted (preparative).
(D) Replication products from 60 min reactions containing 117 mM potassium glutamate in the presence or absence of the essential firing factor Mcm10 on 3 kb CPD^{LEAD} templates were quenched, deproteinized and digested with SmaI. The samples were then split and 2% of each was run in a native gel and processed as for standard reactions (analytical). The remaining 98% were run in separate lanes of the same gel and the full-length bands were excised and electroeluted (preparative).
(E and F) Electroeluted DNA from (D) was digested with BamHI and analyzed by two-dimensional electrophoresis. (D and F) Products generated in the absence of Mcm10 are likely due to replisome-independent template labelling catalyzed by the DNA polymerases in the reaction (Pol α, Pol δ and Pol ε).
(B and E) Full-length products (native gel) arising from leading-strand re-priming, together with their constituent stall and restart products (denaturing gel) are shown in red.

Figure S5. Related to Figure 5.
(A) Schematic illustrating the position of restriction sites for enzymes used to truncate the AhdI-linearized 4.5 kb CPD^{LEAD} templates (top), and the products of restriction enzyme digestion (bottom).
(B) Ethidium bromide stained 1% native agarose gel of digested AhdI-linearized 4.5 kb CPD^{LEAD} templates.

(C and D) Denaturing gel analysis of the experiments in Figures 5B and C respectively. Stall products from the CPD^{LEAD} templates are annotated in red.
(E) Replication reaction of an undamaged template in the presence of the 21 nt re-priming oligonucleotide or its scrambled equivalent.
(F) Comparison of replication on the 3 kb CPD^{LEAD} template with re-priming (R) oligonucleotides or scrambled equivalents (S) that anneal at different distances downstream of CPD^{LEAD} (– : no oligonucleotide). 'Gapped full-length' products are observed with the 1210 nt re-priming oligonucleotide. These products migrate in a distinct position below full-length products due to the size of the ssDNA region between the CPD and re-priming oligonucleotide binding site.
(G) Denaturing gel analysis of the pulse chase experiment in Figure 5F.

Figure S6. Related to Figure 6.
(A) Effect of Pol α concentration on replication of undamaged and 3 kb CPD^{LEAD} templates.
(B) Replication reactions on chromatinized undamaged and 3 kb CPD^{LEAD} templates.
(C and D) Two-dimensional gel analysis of experiments performed on chromatinized undamaged (C) and 3 kb CPD^{LEAD} (D) templates.

Figure S7. Related to Figure 6.
(A and B) Replication reactions performed on undamaged (A) and 3 kb CPD^{LEAD} templates (B) at 20 nM and 200 nM RPA.
(C and D) Two-dimensional gel analysis of experiments performed at 20 nM RPA (C) and 200 nM RPA (D) on the 3 kb CPD^{LEAD} template. Replication products were not digested.
(E-G) Two-dimensional gel analysis of experiments performed with 20 nM Pol α at 10 nM (E), 20 nM (F) and 100 nM (G) RPA on the 3 kb CPD^{LEAD} template. Replication products were digested with SmaI. Lane profiles of the denaturing gel showing the constituents of the full-length products (native) are shown next to each gel.

Table S1. Oligonucleotides used in this study. Related to the STAR Methods (Key Resources Table).

**Oligonucleotides**		
For cloning PstI/BamHI cassette (top): PB_Top: GATCCGCAGTACCAACCTCAGCACTTAAGTCCTCAGCGCAGTACCAACTGCA	This paper	N/A
For cloning PstI/BamHI cassette (bottom): PB_Bottom: GTTGGTACTGCGCTGAGGACTTAAGTGCTGAGGTTGGTACTGCG	This paper	N/A
For cloning SphI/SpeI cassette (top): SS_Top: CTAGTGCAGTACCAACCTCAGCACTTAAGTCCTCAGCGCAGTACCAAGCATG	This paper	N/A
For cloning SphI/SpeI cassette (bottom): SS_Bottom: CTTGGTACTGCGCTGAGGACTTAAGTGCTGAGGTTGGTACTGCA	This paper	N/A
AflII_undamaged: 5'-phos-TCAGCACTTAAGTCC	This paper	N/A
AflII_idSp: 5'-phos-TCAGCACT-/idSp/-AAGTCC	This paper	N/A
AflII_CPD: 5'-phos-TCAGCAC-/CPD/-AAGTCC	This paper	N/A
AflII_competitor: GTTGGTACTGCGCTGAGGACTTAAGTGCTGAGGTTGGTACTGCG	This paper	N/A
SapI_Btn_1: 5'-phos-GCTATGTGGTAGGAAGTGAG-biotin-TEG-3'	This paper	N/A
SapI_Btn_2: 5'-biotin-CTCACTTCCTACCACAT-3'	This paper	N/A
For artificial restart assays: 21 nt re-priming: GGTTGGTACTGCGG	This paper	N/A
For artificial restart assays: 21 nt scrambled: GGTGATCGTTCGGG	This paper	N/A
For artificial restart assays: 265 nt re-priming: CTGGTTTCCGCCGT	This paper	N/A
For artificial restart assays: 265 nt scrambled: GTTCGCCTTGCCGT	This paper	N/A
For artificial restart assays: 1210 nt re-priming: GGCGTCACTACCAC	This paper	N/A
For artificial restart assays: 1210 nt scrambled: GCCCAGTACTGCAC	This paper	N/A
JY180: primase assay: GAATAATGGAAGGGTTAGAACCTACCAT	This paper	N/A
